# The zebrafish transcriptome during early development

**DOI:** 10.1186/1471-213X-11-30

**Published:** 2011-05-24

**Authors:** Liselotte Vesterlund, Hong Jiao, Per Unneberg, Outi Hovatta, Juha Kere

**Affiliations:** 1 Department of Biosciences and Nutrition, and Science for Life Laboratory, Karolinska Institutet, Stockholm, Sweden; 2 Clinical Research Centre, Karolinska University Hospital, Huddinge, Sweden; 3 Department of Clinical Science, Intervention and Technology, Karolinska Institutet, Karolinska University Hospital, Huddinge, Sweden; 4 Department of Medical Genetics, Hartman Institute, University of Helsinki, and Folkhälsan Institute of Genetics, Helsinki, Finland

## Abstract

**Background:**

The transition from fertilized egg to embryo is accompanied by a multitude of changes in gene expression, and the transcriptional events that underlie these processes have not yet been fully characterized. In this study RNA-Seq is used to compare the transcription profiles of four early developmental stages in zebrafish (*Danio rerio*) on a global scale.

**Results:**

An average of 79 M total reads were detected from the different stages. Out of the total number of reads 65% - 73% reads were successfully mapped and 36% - 44% out of those were uniquely mapped. The total number of detected unique gene transcripts was 11187, of which 10096 were present at 1-cell stage. The largest number of common transcripts was observed between 1-cell stage and 16-cell stage. An enrichment of gene transcripts with molecular functions of DNA binding, protein folding and processing as well as metal ion binding was observed with progression of development. The sequence data (accession number ERP000635) is available at the European Nucleotide Archive.

**Conclusion:**

Clustering of expression profiles shows that a majority of the detected gene transcripts are present at steady levels, and thus a minority of the gene transcripts clusters as increasing or decreasing in expression over the four investigated developmental stages. The three earliest developmental stages were similar when comparing highly expressed genes, whereas the 50% epiboly stage differed from the other three stages in the identity of highly expressed genes, number of uniquely expressed genes and enrichment of GO molecular functions. Taken together, these observations indicate a major transition in gene regulation and transcriptional activity taking place between the 512-cell and 50% epiboly stages, in accordance with previous studies.

## Background

Zebrafish (*Danio rerio*) is used as a model system in many different scientific fields due to its rapid development in combination with a relatively short generation time and ease of genetic manipulation [1-6]. However, the most prominent application of zebrafish has probably been within developmental biology. This is due to the ease with which the embryos are obtained, in addition to the transparency of the developing zebrafish embryo, which greatly aids observation of developmental processes.

DNA sequencing has increased tremendously in throughput with the advent of next-generation sequencing (NGS) [7]. Briefly, the technology generates millions of short DNA sequence reads from a sample. The technology has recently been applied to transcriptome profiling [8], in which RNA from a sample is converted into cDNA, fragmented, and sequenced. Denoted RNA-Seq, it offers several advantages as compared to previous profiling applications, such as microarrays or quantitative RT-PCR. Most importantly, RNA-Seq does not rely on predefined probes, and consequently allows for discovery of new transcript variants and for distinction between closely homologous genes [9]. Moreover, alternatively spliced transcripts [10] and non-conding RNAs [11] can be characterized and monitored. In addition, by sustained sequencing, there is virtually no limit in sensitivity, which enables the detection of rare transcripts that may be undetectable in microarray analysis [12].

A more complete characterization of the zebrafish genome, in combination with additional knowledge of the zebrafish transcriptome, would enable access to the full potential of this powerful vertebrate model system. Previous studies have investigated parts of the zebrafish transcriptome during development and in adult tissues [13-20]. In addition, there has been a recent addition of several RNA-Seq tracks of zebrafish early embryos to Ensembl's Zv9. However, the present study is to our knowledge the first study to utilize the new technology of RNA-Seq to compare the transcriptome during early stages of zebrafish development and thereby increasing the known number of developmentally regulated transcripts.

Four early embryonic stages (1-, 16-, 512-cell stage and 50% epiboly) were chosen in order to investigate and compare the transcriptome during early zebrafish development. The newly fertilized egg is in the zygote period until the first cleavage occurs, about 40 minutes after fertilization [21]. At the 1-cell stage the genome is silent and the transcriptome consists by definition of maternal transcripts. The 16-cell stage occurs at 1.5 hours post-fertilization (hpf) and during this time some of the blastomeres are still interconnected. At the 512-cell stage (2.75 hpf) the mid-blastula transition (MBT) begins, the embryo genome is activated and the cell cycles lengthen gradually [22]. In zebrafish development gastrulation starts at the 50%-epiboly stage (5.25 hpf) when the blastoderm margin has moved to 50% of the distance between the animal and vegetal pole [21]. By comparing the 1-cell stage, 16-cell stage, 512-cell stage and 50% epiboly stage gene expression profiles we provide a framework for future investigations of early developmental processes. The aim of this study was to compare the transcriptional profile of four early developmental stages in zebrafish using RNA-Seq, and in addition use these gene expression profiles to identify novel candidate genes with possible key roles during early development. Furthermore, the detection of a number of developmentally interesting gene transcripts in the present study is discussed in relation to previously published observations.

## Results

### Quantifying developmentally expressed transcripts using RNA-Seq

RNA-Seq was used in order to investigate the early zebrafish transcriptome. The RNA-Seq resulted in approximately 73 M total reads from the 1-cell stage, 85 M total reads from the 16-cell stage, 78 M total reads from the 512-cell stage and 79 M total reads from the 50% epiboly stage (Table 1). Out of the total number of reads for each stage 68%, 70%, 73% and 65% respectively, were successfully mapped. From these mapped reads the percentage of uniquely mapped reads for the four stages were 44%, 40%, 38% and 36%, respectively. The uniquely mapped reads had higher quality (median QV > 15 over all positions) compared to multiply mapped reads (median QV < 15 for most positions). Therefore, overall read quality does in part explain low unique mapping rate. However, the fraction of uniquely mapped reads is similar to that previously reported [23]. The sequence data (accession number ERP000635) is available at the European Nucleotide Archive http://www.ebi.ac.uk/ena/.

**Table 1:** RNA-Seq results.

Reads	1-cell stage	16-cell stage	512-cell stage	50% epiboly
Total reads	73527619	85135130	78375125	78598071

Reads mapped	49957154	59543747	57301086	51285866

Reads filtered	967970	1922778	1366280	1105434

Reads unique	22172092	23629460	21623211	18645505

Numbers of total reads obtained during RNA-Seq of the studied developmental stages, numbers of reads mapped to the exon model, numbers of reads being filtered and the numbers of uniquely mapped reads used for further analysis of gene expression.

**Figure 1:**
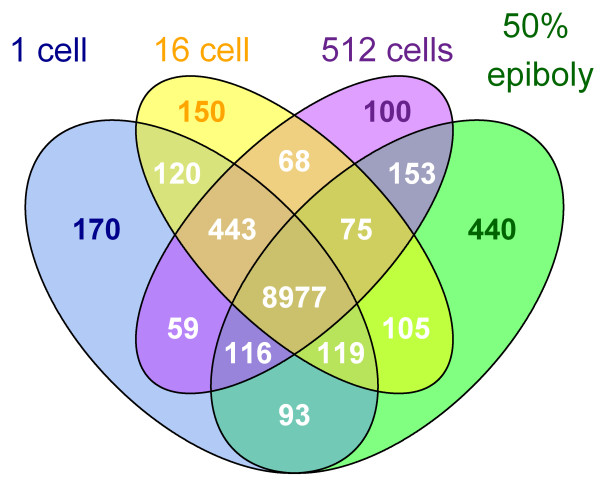
**Venn diagram showing the developmental distribution of the genes detected**. Genes were grouped based on their expression at single or multiple stages. Expressions were evaluated based on the number of reads per gene model. Genes with 0 or 1 read were defined as not expressed. In the four studied developmental stages 2423 transcripts were defined as not expressed.

Reads were assigned to transcripts based on their overlap with the 13610 reference gene models defined in the UCSC RefSeq Genes track. An *ad hoc *cutoff for detectable expression was set at >= 2 reads per transcript. Using this cutoff, 11187 gene transcripts could be detected in the RNA-Seq data set, leaving 2423 transcripts undetected (Figure 1). These 2423 non-detected gene transcripts were enriched in GO molecular functions seemingly unrelated to early development, such as olfactory receptor activity and photoreceptor activity among others (Additional file 1).

In order to investigate the dynamics of the zebrafish early transcriptome the number of detected transcripts shared between the studied developmental stages was determined (Additional file 2). The shared transcripts were 9658 between the 1-cell stage and 16- cell stage, and this number decreased with developmental progression (Figure 1). Thus the 1-cell and 50% epiboly stages shared 9304 transcripts. These observed differences in shared transcripts between the different developmental stages may reflect the transcriptional similarity between stages pre-MBT, and the increased transcriptional heterogeneity of the cellular population post-MBT.

The number for transcripts shared only between two developmental stages was lower, with the 1-cell stage sharing 120 transcripts with the 16-cell stage, 59 transcripts with the 512-cell stage and 93 transcripts with 50% epiboly stage (Figure 1). In turn, 50% epiboly stage shared 153 transcripts with 512-cell stage and 105 transcripts with the 16-cell stage. The number of shared transcripts between the 16-cell stage and the 512-cell stage was 68. These results indicate that transcripts present at the 1-cell stage are depleted during the progression of development, only to reappear in the 50% epiboly stage after the MBT has taken place.

A majority of the gene transcripts shared between the pre-MBT developmental stages coded for membrane-associated proteins, such as members of the solute carrier (Slc) gene superfamily, whereas gene transcripts shared between 512-cell stage and 50% epiboly were involved in transcription regulation and patterning (Additional file 2). This group of shared gene transcripts contains members of the fork head box (Fox) transcription factor family and fibroblast growth factors (Fgfs), which are known to be involved in regulation of developmental processes. The appearance of these transcripts coincides with the cell faith determination and differentiation that takes place during zebrafish gastrulation.

### Gene expression profiling reveals a static expression profile for a majority of genes

**Figure 2:**
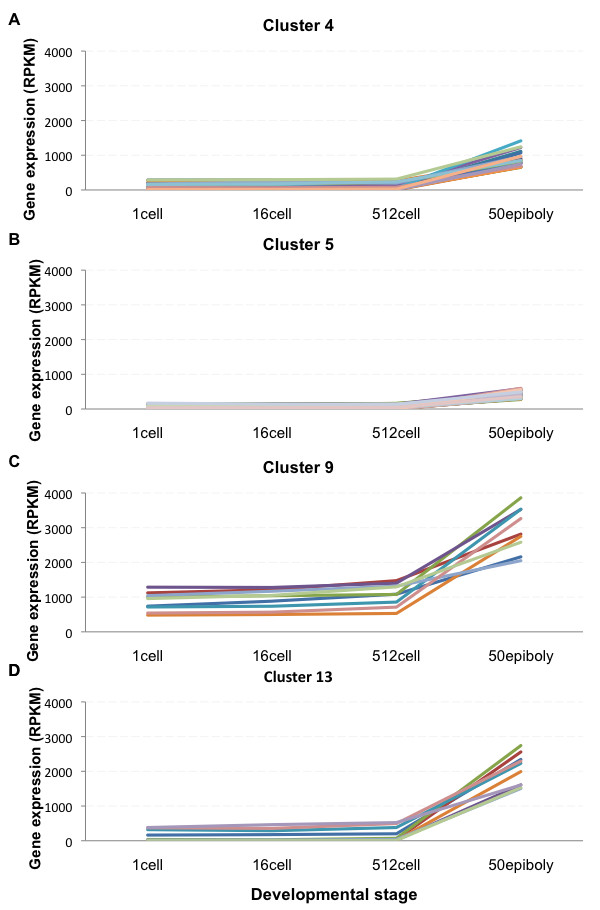
**Gene clustering based on increasing gene expression profiles across the four developmental stages**. Four clusters with an increase in gene expression levels from 1-cell stage to 50% epiboly. A) Cluster 4 contains transcripts with initial low abundance. B) Cluster 5 transcripts (*cldn e*, *slc16a1 *and *slc25a22 *among others) are low in abundance and display a modest increase during development. C) Cluster 9 transcripts (*cirbp*, *ef1a*, *h3f3a*, *hspa8*, *khdrbs1a *and *bactin2 *among others) are relatively abundant at 1-cell stage to increase to RPKM levels above 1000 in 50% epiboly. D) Transcripts in cluster 13 (*apoeb*, *krt18*, *krt8*, *krt4 *and *zgc:85717 *among others) are moderately expressed at early stages and increase to high RPKM levels in 50% epiboly.

**Figure 3:**
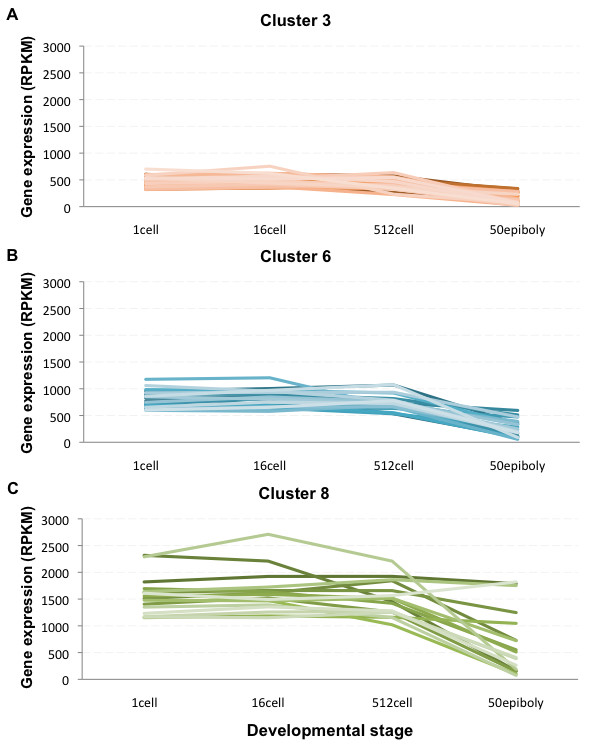
**Clustering based on decreasing gene expression profiles across the four developmental stages**. Three clusters display a decrease in transcript abundance during development. A) Cluster 3 transcripts (*dnmt1*, *plk1*, *plk3*, *slc16a3 *and *slc25a25 *among others) are moderately expressed in early development and decrease to relatively low levels in 50% epiboly. B) Transcripts in cluster 6 are detected at relatively high RPKM levels during early development. C) Cluster 8 transcripts (*ccna1, cldn d, ctssb.1, fth1, gapdh, gmnn, mid1ip1, mt2 *and *zgc:110304 *among others) are highly abundant in 1-cell to 512-cell stage.

To classify the dynamics of the early zebrafish transcriptome on a global scale we performed gene expression profile clustering. The 11187 gene transcripts detected in the RNA-Seq were categorized into 20 different clusters on account of their expression pattern during development (Additional file 3). Four clusters including 93 genes showed visually increasing expression profile patterns with progressing development (Figure 2A-D and Additional file 4) and three clusters containing a total of 84 genes showed decreasing expression profiling patterns (Figure 3A-C and Additional file 4). With 177 gene transcripts detected as developmentally regulated when comparing the transcriptomes of the four developmental stages a majority of gene transcripts thus cluster as present at a steady level. Furthermore, these steady level transcripts are present at different RPKM (reads per kilobase of exon model per million mapped reads) expression levels in the four studied developmental stages.

### GO term enrichment among the differentially expressed genes

In order to determine if there was an enrichment of gene transcripts with related functions in the four studied developmental stages the GO term enrichment of the 11187 detected gene transcripts were investigated. Using GO term finder [24] to search for molecular function enrichment within the subset of genes from the gene expression profiling clusters with an increase in expression during development (Figure 2A-D and Additional file 4), a significant increase in transcripts encoding nucleic acid binding and DNA binding were found (Additional file 5A). This may reflect the developmental transitions taking place during MBT, when the transcription of the embryo genome is activated.

In the data set containing the genes clustering as decreasing during development (Figure 3A-C and Additional file 4), one GO term molecular function was significantly enriched: polo kinase kinase activity (Additional file 5B). This activity is associated with the cell cycle [25] and oocyte maturation [26].

### Variation in high RPKM-level transcript populations during development

The total number of different gene transcripts detected was in the same range for all four developmental stages. A total of 10096 gene transcripts were found in the 1-cell stage as compared to 10056 and 9990 gene transcripts found in the 16-cell stage and 512-cell stage respectively (Figure 1). After MBT, at 50% epiboly stage, the corresponding number of genes found to be expressed was 10077. To investigate whether or not the same transcripts were present a high levels throughout the four studied developmental stages the transcripts with the highest RPKM values in each stage were compared. When ranking the transcripts detected after highest RPKM values, eight out of the ten transcripts found in the 1-, 16- and 512-cell stages were identical (Table 2). However, the ten most abundant transcripts in 50% epiboly stage were not represented in the 1- and 16-cell stage, and only one transcript (*hsp8*) was found among the top ten 512-cell stage genes. Within this group of highly expressed genes were claudins (*cldn g *and *cldn d*), cyclins (*ccna*) and metal-binding proteins (*fth1*, *mt2*) among others. Thus from this observation it appears that the developmental stages up until MBT have a relative high level expression of several identical gene transcripts. Furthermore, with the subsequent activation of the embryo genome a change in the identity of highly expressed genes can be observed.

**Table 2:** The ten most abundant transcripts in each developmental stage.

Rank	1-cell stage		16-cell stage		512-cell stage		50% epiboly	
**1**	cldng	NM_180965	cldnd	NM_180964	cldnd	NM_180964	khdrbs1a	NM_130925

**2**	cldnd	NM_180964	cldng	NM_180965	bactin1	NM_131031	ef1a	NM_131263

**3**	bactin1	NM_131031	bactin1	NM_131031	rrm2	NM_131450	hnrnpa0l	NM_001114881

**4**	ctssb.1	NM_001024409	rrm2	NM_131450	ccna1	NM_212818	h3f3a	NM_212996

**5**	rrm2	NM_131450	zgc:110304	NM_001017593	zgc:110304	NM_001017593	cirbp	NM_001040321

**6**	zgc:110304	NM_001017593	ctssb.1	NM_001024409	fth1	NM_131585	hspa8	NM_001110403

**7**	mt2	NM_001131053	ccna1	NM_212818	gmnn	NM_200086	zgc:85717	NM_213223

**8**	fth1	NM_131585	gapdh	NM_001115114	gapdh	NM_001115114	ctsl1a	NM_212584

**9**	gapdh	NM_001115114	mid1ip1	NM_213439	hspa8	NM_001110403	cirbp	NM_200017

**10**	ccna1	NM_212818	fth1	NM_131585	cldng	NM_180965	apoeb	NM_131098

Pre-MBT and MBT developmental stages (1-, 16- and 512-cell stage) were shown to have similar composition of high abundance transcripts, in contrast to the post-MBT stage (50% epiboly). The RefSeq accession numbers for each detected transcript is given in the table.

### Enrichment of GO molecular functions in gene transcripts detected at high levels

**Figure 4:**
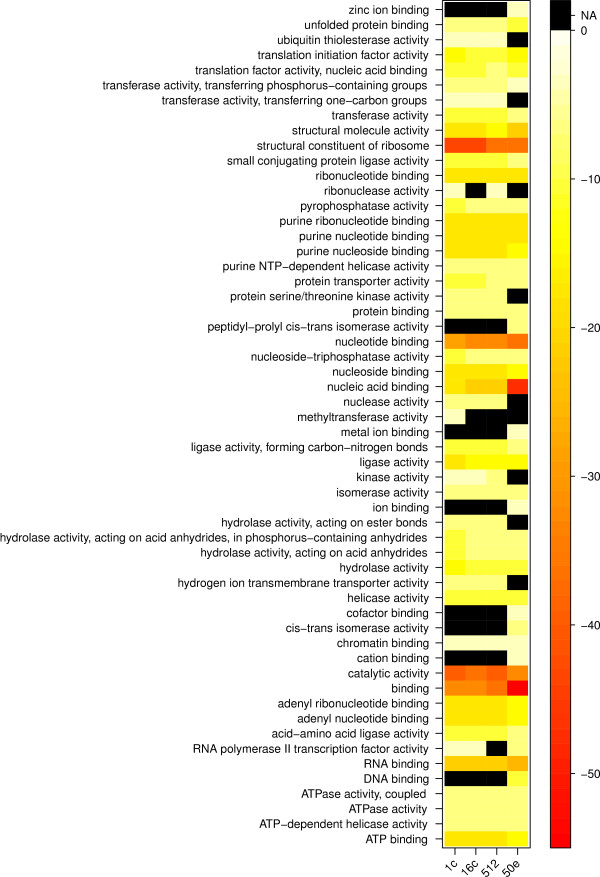
**Heat-map plot of GO function enrichment**. The heat-map plot is based on the 4000 genes with the highest RPKM values in each developmental stage studied. The colors correspond to enrichment significance log values, with black denoting absent category at a given stage.

In order to compare the possible functions of the transcripts identified from the RNA-Seq analysis of the different developmental stages, the 4000 genes with the highest RPKM values for each stage were selected and analyzed for function using GO term finder [24]. Significant enrichment of several GO molecular functions was found in this subset of relatively highly expressed genes (Figure 4).

The earliest three stages showed a high degree of similarity in enriched functions, whereas the 50% epiboly differed from the earlier developmental stages. For example, protein serine/threonine kinase activity was significantly enriched only in the first three developmental stages (Figure 4). Within this group of transcripts the polo-like kinases *plk1*, *plk3 *and *plk4 *can be found. Another molecular function enriched in these three developmental stages was nuclease activity. In this group is the endoribonuclease *dicer1*, which codes for an enzyme involved in miRNA synthesis. Mutating Dicer1 blocks the formation of mature miRNAs and leads to abnormal gastrulation morphology [27]. The enrichment of *dicer1 *in the three pre-MBT developmental stages may reflect Dicer1 involvement in the peak of miRNA levels observed at the onset of MBT with subsequent degradation/silencing of maternal RNAs [28]. Interestingly, methyltransferase activity was the only significantly enriched GO molecular function found in the 1-cell stage, whereas molecular functions significantly enriched exclusively in the 50% epiboly stage included transcription, protein folding/processing and ion binding (Figure 4). Taken together these observations indicate that there is a shift in the molecular functions of the expressed gene transcripts taking place during the MBT, from the early stages enrichment of nuclease activity before MBT, reflecting the developmental importance of miRNA and RNA processing, towards the enrichment of transcription functions in the 50% epiboly stage related to MBT and the onset of transcription.

### A number of transcripts are detected at only one specific developmental stage

Gene transcripts detected at only one developmental stage were investigated for enrichment of GO annotations in order to determine if the developmental transition in gene transcript molecular function enrichment observed for highly expressed gene transcripts also could be detected in stage specific gene transcripts.

The total number of genes detected at one specific developmental stage varied between the stages, with the largest number of detected stage-specific gene transcripts found in the 50% epiboly stage (n = 440), followed by the 1-cell (n = 170), 16-cell (n = 150) and the 512-cell stage (n = 100), respectively (Figure 1). The 170 genes detected in the 1-cell stage displayed no significant enrichment of GO molecular functions. However, when investigating the enrichment of GO term cellular components and biological processes there was significant enrichment of extracellular region components and multicellular organismal process, developmental process, multicellular organismal development, thyroid gland development and anatomical structure development (Additional file 6A).

Interestingly, among the gene transcripts detected at the 16-cell stage (n = 150) and the 512-cell stage (n = 100), there was a significant enrichment of the GO molecular functions associated with transcription (Additional file 6B-C), whereas GO biological process terms and cellular component terms showed no significant enrichment. Similar GO molecular terms were found significantly enriched among the 440 gene transcripts specifically expressed in the 50% epiboly stage (Additional file 6D). Only the GO cellular component cell junction was significantly enriched in this subset and there was no enrichment of GO biological process terms. These observations correlate with the observed change in enriched GO molecular functions for the highly expressed subset of gene transcripts, displaying enrichment in transcription-related gene transcripts with progressing development. However, the number of stage-specific gene transcripts detected was small and the gene transcripts were found present at relatively low RPKM levels making it difficult to assess their biological relevance.

### Validation of gene transcripts detected during zebrafish early development

**Figure 5:**
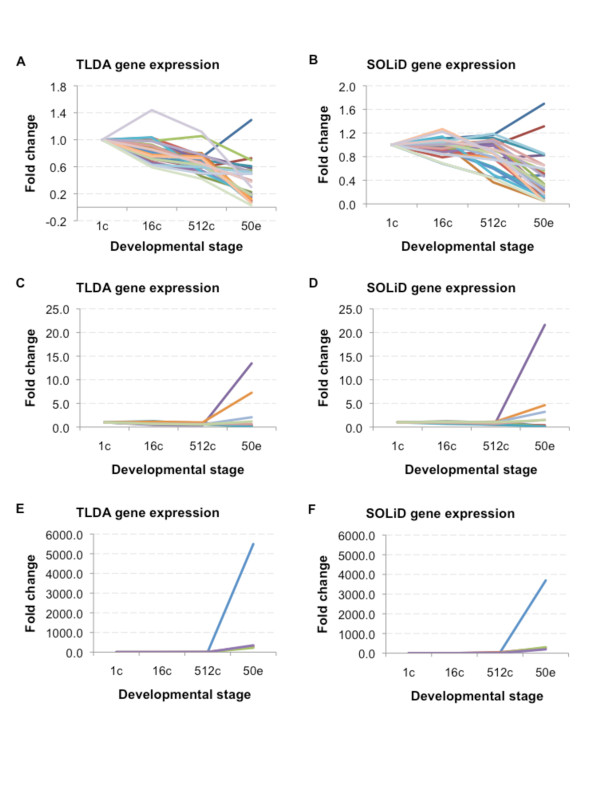
**Comparison of RNA-Seq and TLDA gene expression data**. The expression profile for a subset of genes was validated through comparison between RNA-Seq gene expression data and expression data obtained using TLDA. The gene expression profiles of three different subsets of genes (AB, CD and EF) are given in fold change as detected by TLDA (A, C and E) and RNA-Seq (B, D and F). The AB set contains genes *tdrd7*, *slc2a15*, *slc7a6os*, *slc9a8*, *slc25a1*, *slc25a25*, *slc25a46*, *slc26a11*, *slc35f2*, *slc39a9*, *slc48a1b *among others; the CD set contains *cldn d*, *foxA2 *and *pou5f1 *among others; the EF set contains *cldn e*, *klf4*, *slc25a22 *among others.

**Figure 6:**
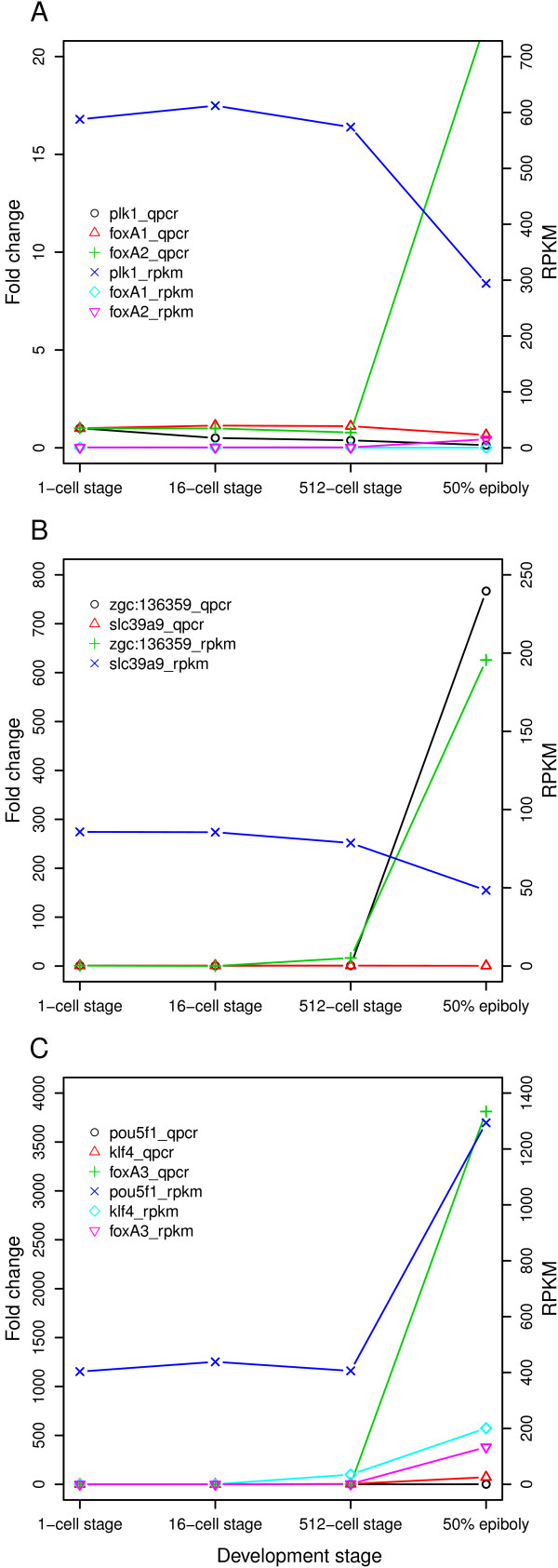
**Determination of specific gene expression profiles using semi-quantitative RT-PCR**. Semi-quantitative RT-PCR was used to investigate specific gene expression profiles during early zebrafish development and correlated to the expression profile obtained using RNA-Seq. Left Y axis indicate fold change, while right Y axis indicate RPKM levels. The corresponding method used are given in the graph legend. A) Gene expression profile for *plk1*, *foxA1 *and *foxA2*. B) Gene expression profile for *zgc:136359 *and *slc39a9*. C) Gene expression profile for *pou5f1*, *klf4 *and *foxA3*.

Several genes were found to vary in expression during the developmental stages investigated. To further compare and validate the expression of a subset of these genes TLDA (Figure 5A-F) and semi-quantitative RT-PCR (Figure 6A-C) were used. Several TLDA gene assays were chosen for their known involvement in vertebrate pluripotency and differentiation processes (Additional file 7). A few developmentally uncharacterized genes that were detected in the RNA-Seq data set were also included. Furthermore, semi-quantitative RT-PCR analysis was used to investigate and validate the detection of a number of developmentally expressed genes, such as *plk1, foxA1, foxA2 *(Figure 6A), *slc39a9, zgc:136359 *(Figure 6B) and *pou5f1*, *klf4 *and *foxA3 *(Figure 6C). The TLDA (Figure 5A-F) and semi-quantitative RT-PCR (Figure 6A-C) analyses showed similar expression patterns as previously observed using RNA-Seq, thus confirming the developmental regulation of the investigated gene transcripts.

### Putative novel transcribed regions detected by RNA-Seq

**Figure 7:**
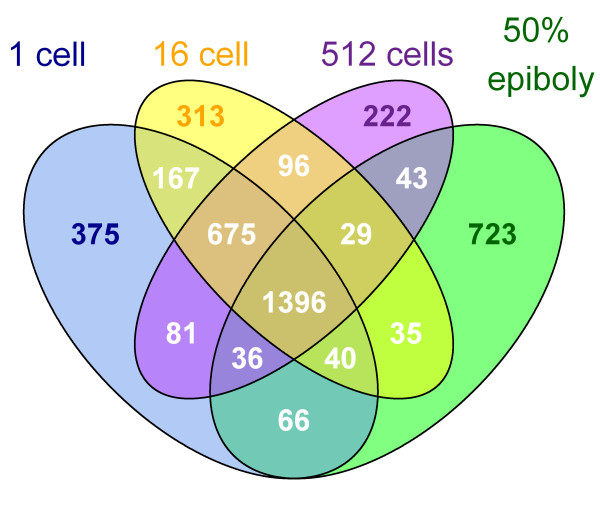
**Venn diagram showing the developmental distribution of the putative novel transcribed region identified from the RNA-Seq**. The total number of detected putative novel transcribed regions in the studied developmental stages was 3068 (1-cell), 2958 (16-cell), 2766 (512-cell) and 2493 (50% epiboly) respectively, with the largest number of stage specific putative novel transcribed regions found in the 50% epiboly stage.

A significant advantage of RNA-Seq compared to microarrays is that it is possible to identify and characterize novel mRNA transcripts. It is likely that there are many yet unidentified transcripts that play important roles in early development. Therefore, in order to identify putative novel transcribed regions, mapped reads were clustered into seqfrags [29]. A seqfrag contains at least two reads with overlapping coordinates on the same strand. Seqfrags with more than 5 reads located at least 20 kb from known UCSC reference genes and ENSEMBL genes [30] were called as putative novel transcribed regions. In this way a total number of 4297 putative novel transcribed regions could be identified (Figure 7). 3068 of these putative novel transcribed regions were identified in the 1-cell stage, followed by 2958, 2766 and 2493 putative novel transcribed regions in 16-cell, 512-cell and 50% epiboly stages respectively. Sharing of transcripts between developmental stages is consistent with our previous results for known genes; sharing is highest between the three first stages (n = 675), and the 50% epiboly stage has the largest amount of unique novel transcripts (n = 723) (Figure 7). This confirms the overall picture of a major transition in transcriptional activity taking place between the 512-cell stage and the 50% epiboly stage. We validated the expression of one putative novel transcribed region located on chromosome 25 by regular PCR amplification from cDNA and subsequent sequencing (for details see Additional file 8). The sequence shows significant similarity to a recently predicted zebrafish protein, a-kinase anchor protein 13 [GenBank: XP684554]. This indicates that we have identified a potentially novel transcript in our RNA-Seq dataset, demonstrating the utility of the RNA-Seq method for annotation and characterization of the zebrafish early transcriptome.

## Discussion

### RNA-Seq detection of developmentally expressed gene transcripts

The number of unique mapped reads obtained using RNA-Seq was on average 21 million for all four studied developmental stages (Table 1) with a total number of 11187 different gene transcripts detected. About 0.5 M reads were mapped to known junctions in each stage. The 2423 gene transcripts that were not detected (Figure 1), showed enrichment of GO functional terms seemingly unrelated to early development, such as olfactory receptor activity (Additional file 1). Apart from being undetected because they are not expressed these transcripts may be undetected due to them not being present in the unfertilized oocyte and thus not carried forward as maternal transcripts, that they are specifically degraded before 1-cell stage or that their expression is too low to be detected with the sequencing depth reached in this study. For example, maternal degradation of *sox11a*, one of the non-detected transcripts, has been previously shown [31], thus offering an explanation to its absence in our dataset.

### Similar numbers of transcripts detected in the four developmental stages studied

The total number of different transcripts found in the four studied stages was very similar, despite the fact that the sample points cover development from 1-cell stage up until 50% epiboly (Figure 1). A degradation of maternal RNA taking place pre-MBT has been reported in zebrafish [15,31] and this may cause the modest decrease observed in unique transcripts that occurs between the 1-cell stage and 512-cell stage. In addition, the number of transcripts found only at one specific stage was low, indicating that the majority of transcripts needed for development beyond 50% epiboly are already present at the 1-cell stage.

### Pre-MBT developmental stages have similar high abundance transcript profile

The ten most abundant transcripts in the four different stages during development were almost identical for the 1-, 16-, and 512-cell stage, whereas in the 50% epiboly stage the most abundant transcripts differed from previous developmental stages (Table 2). These abundant transcripts code mainly for nucleic acid binding proteins (*khdrbs1a, mid1ip1, bactin, hspa8, hnrnpa01, cirbp*), metal ion binding proteins (*fth1, mt2*), cell adhesion proteins (*cldn d, cldn g, zgc:110304*), proteolytic proteins (*ctsl1a, ctssb.1*) and proteins involved in the cell cycle (*ccna1, gmnn, rrm2*). A few of these have been implicated in vertebrate development previously, while the specific developmental roles of the others are yet unknown. For instance Zgc:110304, a putative epithelial cell adhesion molecule, has been implicated in epiboly [32]. From our RNA-Seq data it is indicated that Zgc:110304 may have additional functions during early development, since the transcript is detected at relatively high levels already at 1- and 16-cell stage.

### Early stage-specific transcripts are detected at low expression levels

The subsets of gene transcripts found exclusively in 1-, 16- and 512-cell stages were detected at relatively low RPKM levels. Although the biological relevance of transcripts at such low RPKM values might be uncertain, a number of these transcripts have been previously reported as expressed during early development. For example, in the present study *vegfaa*, *ing5 *and *elovl2 *were among the most abundant transcripts detected only in the 1-cell stage (Additional file 9). In a previous microarray-based study human *VEGFA *and *ING5 *transcripts were found in GV, MI and MII oocytes [33] and using RT-PCR *elovl2 *has been detected in zebrafish embryos at 0 hpf [34]. Vegfaa plays a part in vascular development [35], Ing5 has been shown to be involved in chromatin remodeling [36], and Elovl2 is part of the long-chain polyunsaturated fatty acid biosynthesis pathway [37]. Thus the detection of these transcripts in the 1-cell stage during zebrafish development is plausible and the respective function of these three genes further implies their involvement in later stages of embryo development.

### Methyltransferase activity is enriched at 1-cell stage

It has been postulated that during early development the embryo genome is silent and transcription is activated first at MBT. The observed enrichment of GO methyltransferase activity in the 1-cell stage detected gene transcripts may reflect the developmental chromatin-modifying dynamics taking place (Figure 4). Interestingly, *DNA methyltransferase 1 *(*dnmt1*) was detected in all four stages, although with a decreasing gene expression profile (Figure 3A). Dnmt1 has been shown to direct terminal tissue differentiation in zebrafish and knocking down *dnmt1 *causes an increase in embryo mortality [38]. Thus the observed developmental regulation of *dnmt1 *may reflect the suppression of transcription present pre-MBT.

### Polo kinase kinase activity associated transcripts decrease during development

Interestingly, only one GO term molecular function, polo kinase kinase activity, was enriched in the clusters displaying a decrease in gene expression profile (Figure 3). Polo-like kinases 1-4 (*plk1-4*) were detected already at 1-cell stage suggesting that these transcripts are of maternal origin. Both *plk1 *and *plk3 *were found at relatively high RPKM levels and decrease during development, whereas *plk2 *and *plk4 *were expressed at a lower level throughout the studied time period (Additional file 9). *Plk1 *has been previously reported to be expressed at the 8-cell stage and shown to be central to mitosis [25]. Depletion of Plk1 caused mitotic arrest and embryo death, whereas the structurally related Plk2 and Plk3 were found to be non-essential for embryo development. With the rate of mitosis declining during the development from 1-cell stage to gastrulation [39], the decrease in *plk1 *transcript numbers observed in the present study are likely to be associated with the progressing development and decreasing rate of mitosis. In addition, the present finding of developmental regulation of *plk1 *(Figure 6A) may reflect the importance of precise regulation of Plk1 gene transcript levels in order to preserve genome integrity since increasing or decreasing the Plk1 expression has been shown to cause chromosomal instability [25].

### Developmentally interesting genes detected at 1-cell stage

We find among the 10096 transcripts detected at the 1-cell stage several known maternal gene transcripts, as well as previously unreported maternal transcripts. For example, the tudor domain containing 7 (*tdrd7*) was detected at the 1-cell stage (Figure 5A-B), in accordance with the reported presence of *tdrd7 *in 4-cell embryos [40]. Tdrd7 function is required for development of the correct number and size of germ cell granules in zebrafish germ cells. In addition, *Tdrd7 *has been shown to be a target of miR-430, a miRNA involved in the degradation of zebrafish maternal RNAs pre-MBT [31]. MiR-430 specific targeting of *tdrd7 *may thus explain the decrease in expression levels detected at the 50% epiboly stage (Figure 5A-B). Another interesting find at 1-cell stage was the previously unreported *zgc:136359*, which displayed an increasing expression profile during developmental progression (Figure 6B). Interestingly, the *zgc:136359 *contains a DEP domain known to be involved in signal transduction (reviewed in [41]). This domain can be found in such proteins as Dishevelled (Dvl) in the Wnt signaling pathway, suggesting that *zgc:136359 *might be involved in signaling events during early development. In a recent report *zgc:136359 *was shown to be first expressed in the enveloping layer at 4hpf using whole-mount *in situ *[42]. The discordance in developmental stage of detection in the present study may indicate the higher sensitivity of RNA-Seq when detecting early transcripts. The specific role of zgc:136359 during development is not known. In addition, two of the four so-called Yamanaka transcription factors, *Krüppel-like factor 4 *(*klf4*) and *Pou-domain class 5 transcription factor 1 *(*pou5f1*), were detected already at 1-cell stage and showed a large increase in RPKM value at the 50% epiboly stage (Figure 5A-D and Figure 6C).

### Developmental function of maternal transcripts

Previously reported numbers of maternal transcripts varies between species. In fully-grown zebrafish follicles 11399 different transcripts have been reported [43]. Zebrafish are oviparous animals and subsequently the zebrafish embryo may be more dependent on factors transferred from the female to the egg prior to fertilization than the embryos of viviparous species. It has been speculated that during evolution a shift from zygotic to maternal expression has taken place in oviparous species saving energy and allowing for earlier hatching through the transfer of transcripts [44,45]. However, these transcripts may also function indirectly as a nutrition source providing nucleotides and phosphorous [44] implying that all maternal transcripts do not have to be directly involved in embryonic developmental processes *per se*. This may explain the present finding of a relatively large number of different transcripts detected in the 1-cell stage, as well as to the small number of 1-cell stage specific transcripts (Figure 1).

### Detected increase in gene transcript levels before the onset of MBT

The postulated pre-MBT transcriptional repression may not be global. In fact, an extensive microarray based study by Mathavan *et al*. (2005) reports accumulation of 125 gene transcripts before the onset of MBT [15]. In the present study we observe a small subset of genes with increasing RPKM levels at the start of the MBT, but several of the genes previously reported do not correlate with our RNA-Seq expression profiles (50% of the genes display a correlation of <0.30) (Additional file 10). This may be due to differences in embryo sample time point and/or methodological differences between microarray and RNA-Seq. Furthermore, the peak in transcript accumulation detected at 68/128-cell stage by Mathavan *et al*. (2005) need not persist until the 512-cell stage and may therefore not be detected in the present study. In order to further confirm the possibility of pre-MBT transcription a number of candidate genes were selected (eukaryotic translation initiation factor 1B (*eif1b*), sideroflexin 2 (*sfxn2*), zinc transporter solute carrier family 39, member 7 (*slc39a7*), cytotoxic granule-associated RNA binding protein 1, like (*tia1l*), transformer-2 alpha (*tra2a*)), and their developmental expression pattern analyzed by semi-quantitative RT-PCR. In the expression analysis sideroflexin 2 (*sfxn2*) and transformer-2 alpha (*tra2a*) showed a modest accumulation before the onset of MBT, whereas the zinc transporter *slc39a7 *displayed a 2-fold increase in relative transcript levels already at 16-cell stage (Additional file 11). *Sfxn2 *on the other hand has been shown previously to be increased at the 64-cell stage [15] and the present data indicates that this accumulation takes place after the 16-cell stage (Additional file 11). The early accumulation of *slc39a7 *could also be observed in the RNA-Seq data set (Additional file 9). The accumulation of transcripts at different developmental stages prior to MBT may indicate their involvement in specific processes such as transcriptional activation of the zygotic genome and make them suitable candidates for future functional studies.

### Fox gene family transcription level increase coincides with MBT

Members of the Fox gene family were detected as shared between the 512-cell stage and 50% epiboly stage (Additional file 2) and shown to vary in expression levels during development (Figure 6A and 6C). The expression of these genes has been reported during gastrulation and neurulation [46], but the role of the Fox gene family during zebrafish pre-MBT development has not been completely characterized. FoxA has been suggested to be an early transcription factor able to open up compacted chromatin structures, thereby facilitating the binding of other transcription factors [47]. Thus the detected upregulation of *foxA2 *(Figure 6A) and *foxA3 *(Figure 6C) transcripts between 512-cell stage and 50% epiboly may indicate a role in the onset of transcription during zebrafish early development.

### Enrichment of nucleic acid binding and structural molecule activity function post-MBT

The four clusters displaying an increase in RPKM values during development (Figure 2A-D) were significantly enriched for GO molecular functions such as nucleic acid binding, structural molecule activity, DNA binding and binding (Additional file 5A). Similar profiles during early development have been previously reported in other teleost species [15,48]. This enrichment profile may reflect the need for DNA binding molecules such as transcription factors for the activation and continued transcription of the genome. In turn, the increase in structural molecule activity might be explained by the factors needed for the increase in cell number that takes place during development, as well as by the involvement of structural molecules in the gastrulation process. If considering only cell structural molecules, the very early cleavages essentially consist of one large cell that is being rapidly cleaved into many smaller cells, and thus the need for structural components will increase in later stages of development (Additional file 12). Accordingly, we find in this group several cytokeratins, such as *krt8 *and *krt18 *(Figure 2D andAdditional file 4), which have been shown to be present at protein level during post-MBT development [49]. Interestingly, disrupting the expression of the Fox gene family member FoxH1 during zebrafish development has been shown to significantly lower *krt8 *and *krt18 *levels, disrupt gastrulation and cause death at 1 day post fertilization [50]. Increasing levels of *foxH1 *is detected during the studied developmental stages and correlates with the observed increase in *krt8 *and *krt18 *levels (Additional file 9). Another transcript found in these clusters is tight junction component Cldn e. Cldn e was recently reported to be required for zebrafish epiboly [51]. Accordingly, we find the *cldn e *to increase in abundance from 512-cell stage to 50% epiboly (Figure 2B, 5E-F).

### Post-MBT stage specific transcripts are detected at relatively high levels

In comparison to the 1-to 512-cell stage specific gene transcript levels, the transcripts found only at the 50% epiboly stage have relatively higher RPKM values. The higher abundance of transcripts at this stage may be a result of the activation of transcription and the appearance of novel post-MBT transcripts. Accordingly, in this subset of genes we find an enrichment of GO molecular functions such as sequence specific DNA binding, receptor binding, nucleic acid transcription factor activity and sequence specific factor activity (Additional file 6D).

### Claudin f transcript is only detected after the onset of epiboly

Among the most abundant gene transcripts detected only in 50% epiboly is *claudin f *(*cldn f*) (Additional file 6). Claudins have been shown to accumulate to relatively high transcript levels during early zebrafish development [15] and are involved in cell adhesion (reviewed in [52]). Interestingly, the *cldn f*-related *cldn d *and *cldn g *are detected at high RPKM levels from the 1-cell stage up until the 512-cell stage (Figure 5A-B and Additional file 9), only to decrease in relative abundance during 50% epiboly (Table 2). This observation indicates different developmental functions for these three cell adhesion proteins. In accordance with this, Clelland and Kelly (2010) show *cldn d *to be highly expressed in ovary but absent from many other adult tissues. Furthermore, they reported a low but detectable expression of *cldn f *in the eye, gill and ovary [53].

### Enrichment of metal ion binding molecular functions after the onset of MBT

From our results we observe a significant enrichment of GO molecular functions such as ion binding, cation binding, metal ion binding, zinc ion binding and DNA binding in the 4000 highest expressed genes during the 50% epiboly stage (Figure 4). The upregulation of these transcripts may reflect the molecular function demands post-MBT. This is plausible taking into account that metal ion and metal ion interactions play an important role in conformation, topology, stability and folding kinetics of nucleic acids [54,55]. Furthermore, metal ions may also play an important role in RNA function (reviewed in [56]). In addition, transcription factors have been shown to be dependent on zinc and other metals for their activity and specificity [57,58]. Subsequently, we find many zinc finger motif containing protein-coding genes in the GO term metal binding enriched gene subset. The importance of metal homeostasis during embryonic development has been shown [59] and the deleterious effects of heavy metals on embryonic development in fish are well known (reviewed in [60]). Interestingly, asymmetric distribution of metals has been shown in Xenopus (*Xenopus laevis*) oocytes and is believed to provide a framework for cell patterning during development [61]. Thus the upregulation of genes with GO molecular function metal ion binding and zinc ion binding may reflect not only the start of transcription activity but also a need of a precise concentration and distribution of metals throughout the developing embryo.

### Role of membrane-bound transporters during early development

During development the transport and gradient of different factors are important for the growth and differentiation of the embryo. In addition, several non-endogenous compounds such as drugs and environmental toxins are able to use these transporters [62]. Exposure to non-endogenous compounds during early development often leads to death or malformation of the growing organism therefore it is of medical and pharmacological interest to obtain further understanding of the function of these transporters in embryo development. The Slc gene superfamily consists of membrane-bound transporters (reviewed in [63]) and several of these were detected in the four developmental stages studied (Figure 5 and Additional file 13). Interestingly, the metal ion transporter solute carrier family 39 (Slc39) had several members among the most abundant transcripts detected. In addition, *slc39a7 *belongs to the subset of gene transcripts displaying an increase in detected expression levels before MBT (Additional file 11). Another member of the Slc39 subfamily detected was *slc39a9 *(Figure 5A-B and Figure 6B), which has been shown to be important in regulating zinc homeostasis in cells [64]. It has been suggested that Slc39a7 mediated release of zinc from the ER is required for tyrosine kinase activation [65]. Inhibition of tyrosine kinase activity has been shown to cause developmental arrest at the beginning of epiboly [66], therefore the observed pre-MBT increase of *slc39a7 *may be associated with the tyrosine kinase activity required for zebrafish gastrulation.

### Discovery of putative novel transcribed regions

Recent studies have suggested that transcription may take place from a larger part of the vertebrate genome than previously thought [29,67,68], thus a large number of unknown novel genes may yet remain to be discovered. When searching the RNA-Seq data for putative novel transcribed regions during development a total of 4297 such regions could be detected in the four developmental stages studied (Figure 7). The largest number of stage-specific putative novel transcribed regions was found at 50% epiboly (n = 723), perhaps reflecting a more transcriptional permissive state of the genome after MBT. The observation of these putative novel transcribed regions shows the potential of RNA-Seq for discovering novel gene transcripts and exemplifies one of the benefits of RNA-Seq compared to microarray gene expression analysis. With the increased accuracy of zebrafish genome sequencing and the development of new RNA-Seq analyzing software, it may be possible to extract new information from our dataset and make additional observations that are cumbersome by the tools available at present.

## Conclusions

**Figure 8:**
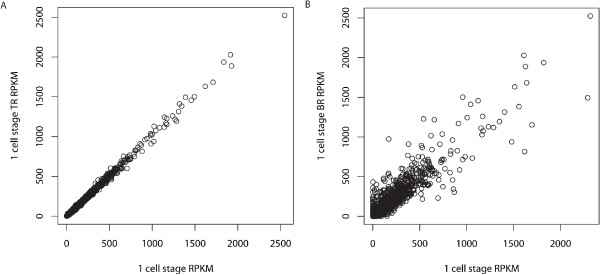
**Correlation plot for 1-cell stage**. A) Technical replicate of 1-cell stage. B) Biological replicate of 1-cell stage. For technical replication comparison r2 is 0.99 (p < 2.2e^-16^), and for biological replication comparison r2 is 0.88 (p < 2.2e^-16^).

In the present study RNA-Seq was used to describe the expression profiles of 11187 gene transcripts detected during early zebrafish development. With the transition from the 1-cell stage to 50% epiboly a number of gene transcripts with different GO molecular functions from previous stages becomes significantly enriched among the most highly expressed genes indicating a switch in the developmental transcriptome (Figure 4). In addition, several genes are shown to be present at the 1-cell stage and developmentally regulated during zebrafish early development. A number of gene transcripts detected in this study has been previously implicated in developmental processes thus corroborating our findings. Moreover, we show that RNA-Seq may be used for investigating expression profiles of developmentally uncharacterized gene transcripts as well as screening for putative novel transcribed regions thus providing a descriptive base for developmental studies. The characterization of the zebrafish early transcriptome presented in this study provides a stepping-stone for further studies into the earliest processes taking place during vertebrate development. With the aid of new sequencing technology it is possible to obtain a vast amount of information about the early zebrafish transcriptome and the changes in global gene profiles during development. A potential concern of the current study is the lack of biological replicates. Replicates were performed of the 1-cell stage to assess reproducibility, with technical replicate showing high (r2 = 0.99) reproducibility, and biological replicate somewhat lower (r2 = 0.88) reproducibility (Figure 8). The pooling of a large number of embryos somewhat remediate this problem, since the detected expression levels are population averages. In addition, both TLDA and semi-quantitative RT-PCR validates the expressional profiles obtained for several gene transcripts present in the RNA-Seq data set. However, small differences in expression should still be interpreted with caution. Despite these issues, our data serve as a valuable resource for further validation and meta-analyses of zebrafish early development. Thus the observations presented in this study may contribute to the many research fields that are using zebrafish as a model system and add knowledge to developmental biology as such. Considering the on-going improvement of RNA-Seq methodology and the recent possibility of sequencing single-embryo transcriptomes [69], we will focus future studies on describing zebrafish development on a single-embryo level and combining transcriptome analyses with studies of the early proteome.

## Methods

### Embryo collection

The zebrafish were kept on a light-dark cycle of 14h/10h at 28.5°C. The mating was performed in 3-liter aquaria using a net to keep the fish separated from the fertilized eggs. The eggs were collected immediately after fertilization, cleaned and kept in aquaria-system water at 28.5°C. The embryos were staged by developmental time and observations of developmental stage. The developmental stages collected for this study were 1-cell (0.75 hpf), 16-cell (1.5 hpf), 512-cells (2.75 hpf) and 50% epiboly (5.25 hpf) stages. When the specific developmental stage was reached embryos were immediately collected in 1.5 ml screwcap tubes containing RNAlater (Ambion), and subsequently stored at -80°C until RNA extraction. The research protocol was approved by the Swedish Ethical Board (application number N230-10 and S170-08).

### RNA extraction and RNA-seq library construction

Total RNA was extracted from approximately 150 embryos per developmental stage using Trireagent (Sigma-Aldrich). The total RNA was then processed further according to the Small RNA Expression Kit (Applied Biosystems). In short, the quality of the total RNA was determined using Agilent Bioanalyzer, and 10 μg total RNA with a RNA integrity number (RIN) of 9.7 or higher was depleted of ribosomal RNA using the Ribominus kit according to the protocol (Invitrogen). RNase III (Ambion) was used for RNA fragmentation. Further RNA-Seq library construction was performed according to the Small RNA Expression Kit protocol (Applied Biosystems) using 200 ng of fragmented RNA as input.

### SOLiD system bead library sequencing

Templated bead preparation was made according to the Template Bead Preparation Guide for Applied Biosystems SOLiD 3 Plus System (Applied Biosystems). The templated bead libraries were quantified using the workflow analysis procedure described in the Instrument Operation Guide (Applied Biosystems). The library was sequenced using SOLiD Opti Fragment Library Sequencing kit Master Mix 50 chemistry (Applied Biosystems) giving 50 bp reads. Sequencing was done on a SOLiD System Sequencing platform version 3 plus (Applied Biosystems).

To assess the quality of the sequencing quality value box plots were generated using the FASTX toolkit [70] (Additional file 14).

### Read mapping and expression evaluation

RNA-Seq sequencing reads were analyzed using whole transcriptome software tools [71] from Applied Biosystems. Briefly, the reads generated from each sample were subjected to a filtering step to remove other sources of RNA such as ribosomal RNA. All filtered reads were then mapped to the zebrafish genome Zv7 assembly [72] by using the Split Read-Mapper Program. The program splits reads into two fragments starting from either end (25 bp from the left, 30 bp from the right), and each fragment is mapped to the reference genome independently. During the mapping phase up to two mismatches were allowed. The mapping of each anchor was extended as far as possible. The resulting alignments from both left and right ends were merged. The alignment score is the sum of scores at each position in the alignment. The score at each position is +1 for a match and -1 for a mismatch. Matching locations were subsequently used to generate counts for annotated features, exons, transcripts or genes using University of California Santa Cruz (UCSC) RefSeq Genes track for exons genomic locations of known transcripts or coverage files (wiggle format). Only uniquely aligned reads with min score >= 24 were used for read counting based on exon models for zebrafish [73] by using Count Tags Program. Finally, expression of a gene was calculated as the number of reads per kilobase of exon model per million mapped reads (RPKM) [74].

### Data mining

For a gene to be detected as expressed, the cutoff was arbitrarily set at more than 1 read per gene, thus genes with 0 or 1 read were considered as not expressed. To find groups of genes with a high degree of similarity in expression profiles across the four developmental stages, RPKM values were clustered using K-means method via GeneSpring (Agilent Technologies). The number of clusters was fixed to 20 and similarity was evaluated by Euclidean measurement. The maximum number of iteration was fixed to 50. Clusters were scored visually as increasing or decreasing, and clusters not grouped into these two categories were assigned as steady state.

### Identification of putative novel transcribed regions

Putative novel transcribed regions were identified by clustering mapped reads into seqfrags [29], where a seqfrag contains at least two reads with overlapping coordinates on the same strand. Seqfrags with more than 5 reads located at least 20 kb from known UCSC reference genes and ENSEMBL genes [30] were called as putative novel transcribed regions. The cutoffs were chosen based on previous studies [74]. Seqfrags were grouped into putative transcribed regions based on the size of intervening regions. By classifying these as intronic two consecutive regions would be grouped into a transcribed region. A simple decision boundary was estimated to 17.6 kb by solving the equation ratio p(x|intron)/p(x|intergenic) = 1, where the probability densities were assumed normal. Based on the reference gene annotation file, the mean intronic length in zebrafish was approximated to 2.8 kb (sd 5.6 kb) and mean intergenic length to 97 kb (sd 164 kb). To test this a candidate was selected for further confirmation by visual examination of the chromosomal region (Additional file 8). Primers were designed using Primer3 [75] to recognize the putative novel transcribed regions and the corresponding fragments were amplified from cDNA by PCR. The cDNA was synthesized as described previously. The amplified PCR fragments were subcloned into the PGEM-T vector (Promega) or the pCR^®^2.1-TOPO vector (Invitrogen) and sequenced (Eurofins mwg GmbH) to confirm identity.

### TaqMan low density array (TLDA) analysis of gene expression

A TaqMan^® ^array micro fluidic card (TLDA, Applied Biosystems) was designed in order to study the expression of a subset of genes expressed during development. Total RNA was extracted using Trireagent (Sigma-Aldrich). The integrity of the RNA was determined using nanodrop 8000 (Thermoscientific) and 1 μg of total RNA with a 260/280 ratio above 1.95 were used for cDNA synthesis (Superscript III, Invitrogen). The reaction mixture containing 50 μl cDNA template (200 ng) and an equal volume of TaqMan^® ^universal master mix (Applied Biosystems) was added to each line of TLDA after gentle vortex mixing. The samples were run on a 79000HT Fast-Real-Time PCR System (Applied Biosystems) and the cycling conditions were as follows: 2 min at 50°C, 10 min at 94.5°C and 30 s at 97°C, and 1 min at 59.7°C for 40 cycles. The threshold cycle Ct was automatically given by SDS2.2 software package (Applied Biosystems). The TLDA contains 18s rRNA and beta actin (actb) gene assays for control. Data derived from the TLDA were normalized against the average values of the two control genes. Biological duplicates for the studied developmental stages were analyzed. Gene expression results are presented as delta Ct values (dCt) and delta delta Ct values (ddCt). The dCt values were obtained by subtracting the Ct values of target genes from Ct values of the average Ct from controls. For ddCt values, additional subtractions were performed by comparison values at 1 cell stage as reference. Relative quantities (RQ) were determined using the equation: RQ = 2-ddCt. All sample were generated two times (separate TLDA cards) and expressed as mean +/- SE.

### Gene expression analysis using semi-quantitative RT-PCR

RNA was extracted as before and a total of 3 pools of 100 embryos from each developmental stage were used for expression analysis. 1 μg total RNA from each of the developmental stages was used for cDNA synthesis. The cDNA reactions were made in a volume of 20 μl using random hexamers and oligo (dT)_20 _primers (Superscript III First-strand synthesis system, Invitrogen). Gene transcript specific primers for *eif1b*, *foxA1*, *foxA2*, *foxA3*, *klf4*, *plk1*, *pou5f1*, *sfxn2*, *slc39a7*, *slc39a9*, *tia1l*, *tra2a *and *zgc:136359 *(Additional file 15) were designed using Primer 3 [75]. Primer pairs were designed in different exons and purchased from Sigma-Aldrich. Primers were used at a final concentration of 100 nM, after proven equally efficient by calibration curves. Semi-quantitative RT-PCR was done using Fast SYBRGreen master mix (Applied Biosystems). Melting curve analysis, agarose gel electrophoresis and sequencing (Eurofins mwg GmbH) were performed to ensure the correct PCR products from each primer pair. Semi-quantitative RT-PCR measurements of individual cDNAs were performed on the 7500 Fast Real-Time PCR system (Applied Biosystems) using a 10 μl mixture containing SYBR green PCR master mix (Applied Biosystems), 1 μl cDNA and forward and reverse primers. Gene transcripts were assayed using duplicate reactions and biological triplicates in MicroAmp Fast Optical 96-well reaction plates (Applied Biosystems). Relative expression of the different gene transcripts was calculated by the delta-delta-Ct (ddCt) method and converted to the relative expression ratio (2^-ddCt^). All data were normalized to the endogenous reference genes actb or rpl13a. Fold change was calculated using expression at 1-cell stage to 1. Values are presented as average fold change. The Ct-values for the reference genes in the cDNA samples varied from 15 to 18.

### Gene molecular function enrichment

Gene molecular function enrichment in different gene subsets was determined using Generic Gene Ontology (GO) Term Finder [24]. GO Term Finder finds significant GO terms in a list of genes and each gene product may be represented by three independent structured, controlled vocabularies; namely molecular function, biological process and cellular component.

## Abbreviations

hpf: hours post fertilization; MBT: mid-blastula transition; RPKM: reads per kilobase of exon model per million mapped reads; RT-PCR: real-time polymerase chain reaction; TLDA: TaqMan low density array.

## Authors' contributions

LV designed the study, performed the experiments, contributed to the interpretation of the data and wrote the manuscript; HJ performed the RNA-Seq mapping, analyzed the data, contributed to the interpretation of the data and helped in drafting the manuscript. PU analyzed the data, contributed to the interpretation of the data and helped in drafting the manuscript. OH and JK were involved in the conception of the study and in revising the manuscript. All authors read and approved of the final manuscript.

## Supplementary Material

Additional file 1 **Go term enrichment in transcripts not detected by RNA-Seq**. Table giving the significant GO term enrichments for biological process, molecular function and structure component in the set of 2423 gene transcripts not detected using RNA-Seq (p < 0.01). Click here for file

Additional file 2 **Gene transcripts shared between different developmental stages**. The table gives the official gene symbols and the RefSeq accession numbers for gene transcripts detected in the indicated developmental stages. The stages between which the transcripts are shared are indicated in the table. Click here for file

Additional file 3 **Expression profile clustering of detected transcripts**. Genes with a high degree of similarity in expression patterns across the four developmental stages RPKM values were clustered using K-means method via GeneSpring (Agilent Technologies). The number of clusters was fixed to 20 and similarity was evaluated by Euclidean measurement. The maximum number of iterations was fixed to 50. Click here for file

Additional file 4 **Gene transcripts present in the expression profile clusters**. Expression profile clustering information for the detected gene transcripts. Click here for file

Additional file 5 **Significant enrichment of GO molecular functions**. A) GO molecular functions significantly enriched for the four increasing clusters. B) GO molecular functions significantly enriched within the three clusters displaying a decrease in transcript abundance during development (p < 0.01). Click here for file

Additional file 6 **The GO term enrichment in developmental-stage specific transcripts**. The significantly enriched GO terms for transcripts found only in one out of the four studied developmental stages were investigated. A) Significantly enriched cellular component and biological processes for the 1-cell stage. Out of the 170 transcripts uniquely found in this stage 47 transcripts were unknown. B) Enrichment of GO molecular function in the 150 transcripts uniquely found in the 16-cell stage. In this set 55 transcripts were unknown. C) Among the 100 transcripts found only in the 512-cell stage 31 were unknown and the remaining 69 were enriched for 2 GO molecular functions. D) Significantly enriched GO cellular components and molecular functions among the transcripts found exclusively in the 50% epiboly stage. Out of the 440 transcripts in this group 125 were unknown. (p < 0.01). Click here for file

Additional file 7 **Gene transcript assayed using TaqMan low density array (TLDA)**. The TLDA gene expression assay numbers (assay ID), the RefSeq accession numbers (RefSeq) and the official gene symbol for the assayed transcripts are given in the table. In addition, the amplicon length is given. Transcripts further assayed using semi-quantitative RT-PCR are marked with a *. Click here for file

Additional file 8 **Putative novel transcribed region identified from the RNA-Seq**. Validation of a putative novel transcribed region. The illustration shows selected tracks from the UCSC viewer http://genome.ucsc.edu in a region on chromosome 25 that lacks annotated transcripts. The four upper tracks show the mapped RNA-Seq reads for each developmental stage, in which the exon structures can be identified via the piled-up reads. The bottom track shows the results of a blat alignment of the sequenced PCR products to the genome. The PCR products and RNA-Seq reads indicate similar exon structures. Click here for file

Additional file 9 **RPKM values for a selected subset of developmentally expressed genes**. The table gives the RPKM values for a subset of developmentally expressed genes that are discussed in the present study. Click here for file

Additional file 10 **Correlation plot between previous reported pre-MBT accumulated transcripts and transcripts detected in the present study**. Plot showing the correlation between Mathavan *et al*. (2005) microarray data and RNA-Seq RPKM values from the present study. The correlations are based on the comparisons of stages 4 cell - 1 cell, 64 cell - 16 cell, and 6 hpf - 50% epiboly, where the first stage in each pair corresponds to the average expression value in the Mathavan *et al*. (2005) data set. Click here for file

Additional file 11 **Validation of pre-MBT accumulation of gene transcripts using semi-quantitative RT-PCR**. Mathavan *et al*. (2005) reported a pre-MBT accumulation of *eif1b*, *tra2a*, *tia1l *and *sfxn2*, which could not be reliably validated in this analysis. The zinc transporter gene transcript *Slc39a7 *was detected as increasing between 1-cell stage and 16-cell stage in the RNA-Seq dataset. The semi-quantitative RT-PCR expressional analysis confirms the pre-MBT accumulation of *slc39a7*. The analysis was made using duplicate samples of biological triplicates from each developmental stage. Click here for file

Additional file 12 **Bar-plot of structural gene transcript expression during development**. Bar-plot showing the expression profile of a number of structural gene transcripts in the four developmental stages studied. The different stages are indicated using following legends: 1c - 1-cell stage; 16 c - 16-cell stage; 512 c - 512-cell stage and 50 e - 50% epiboly. Click here for file

Additional file 13 **Slc gene superfamily members detected using RNA-Seq**. Gene transcripts from 154 different members of the Slc gene superfamily were detected in the four studied developmental stages. Click here for file

Additional file 14 **Sequence quality for the different developmental stages studied**. Box plot of phred quality value distributions by read position using FASTX toolkit to assess the sequencing quality. The different stages are indicated using following legends: 1c - 1-cell stage; 16 c - 16-cell stage; 512 c - 512-cell stage and 50 e - 50% epiboly. Click here for file

Additional file 15 **Primers used in RT-PCR gene expression analysis**. Table of RT-PCR primers used for the study of gene expression of selected genes expressed during early zebrafish development. * denotes endogenous control. Click here for file
