# Supplementary material for: The zebrafish transcriptome during early development
Source: BMC Dev Biol. 2011 May 24;11:30. doi: 10.1186/1471-213X-11-30 (PMC3118190; doi:10.1186/1471-213X-11-30)
Supplement: Additional file 1 — Go term enrichment in transcripts not detected by RNA-Seq. Table giving the significant GO term enrichments for biological process, molecular function and structure component in the set of 2423 gene transcripts not detected using RNA-Seq (p < 0.01). [file 1471-213X-11-30-S1.PDF]

Additional file 1: Significant GO terms enriched in the subset of 2423 genes not expressed (p<0.01).

| GO term | Biological Process | Molecular Function | Structure component |
|---|---|---|---|
| Significantly enriched GO terms for the 2423 genes not found | G-protein coupled receptor protein signaling pathway<br>cell surface receptor linked signaling pathway<br>unannotated<br>signaling pathway<br>signaling<br>gas transport<br>oxygen transport<br>immune response<br>sensory perception<br>neurological system process<br>phototransduction<br>visual perception<br>sensory perception of light stimulus<br>antigen processing and presentation of peptide or polysaccharide antigen via MHC class II<br>signaling process<br>signal transmission<br>detection of light stimulus<br>signal transduction<br>defense response to Gram-negative bacterium | olfactory receptor activity<br>G-protein coupled receptor activity<br>transmembrane receptor activity<br>receptor activity<br>signal transducer activity<br>molecular transducer activity<br>unannotated<br>receptor binding<br>hormone activity<br>oxygen transporter activity<br>oxygen binding<br>photoreceptor activity<br>ligand-gated ion channel activity<br>ligand-gated channel activity<br>endopeptidase inhibitor activity<br>endopeptidase regulator activity<br>G-protein coupled amine receptor activity | integral to membrane<br>intrinsic to membrane<br>membrane part<br>extracellular region<br>cell periphery<br>plasma membrane<br>membrane<br>connexon complex<br>hemoglobin complex<br>gap junction<br>MHC class II protein complex |
