# Supplementary material for: The zebrafish transcriptome during early development
Source: BMC Dev Biol. 2011 May 24;11:30. doi: 10.1186/1471-213X-11-30 (PMC3118190; doi:10.1186/1471-213X-11-30)
Supplement: Additional file 2 — Gene transcripts shared between different developmental stages. The table gives the official gene symbols and the RefSeq accession numbers for gene transcripts detected in the indicated developmental stages. The stages between which the transcripts are shared are indicated in the table. [file 1471-213X-11-30-S2.PDF]

Additional file 2: Uniquely shared gene transcripts

1cell-16cell		1cell-512cell		1cell-50% epiboly	
abcg2d	NM_001042772	helt	NM_207065	atp1a1a.3	NM_131688
erbb4	NM_001143751	lbx1a	NM_001163312	gpm6ab	NM_214687
klf7	NM_001020643	LOC796649	NM_001110761	bach1	NM_001020663
lgi2b	NM_001039642	zgc:112300	NM_001030268	si:ch211-243g18.2	NM_001044910
sc4mol	NM_213353	zgc:136908	NM_001039928	zgc:158652	NM_001083020
ssp2	NM_212840	zgc:173915	NM_001109850	zgc:162320	NM_001110452
zgc:152698	NM_001079934	slc5a1	NM_200681	zgc:77101	NM_212686
zgc:162208	NM_001089560	zgc:112199	NM_001017707	opn1lw2	NM_001002443
zgc:56295	NM_201115	LOC797338	NM_001142375	tceb3	NM_200121
zgc:77076	NM_212889	ndel1a	NM_201344	vsg1	NM_001030244
mpx	NM_212779	zgc:171544	NM_001114742	zgc:136902	NM_001040359
sim2	NM_131836	nkx2.5	NM_131421	rds2	NM_131566
tmem88a	NM_205670	zgc:114084_dup1	NM_001030213_dup1	zgc:162235	NM_001110465
zgc:112118	NM_001017740	cadm1b	NM_001113552	rtn1a	NM_001029967
etnk2_dup2	NM_001034175_dup2	itm2ca	NM_001002549	si:dkeyp-2e4.6	NM_001130654
si:ch211-15p9.2	NM_001044990	pdk3b	NM_001080688	zgc:66298_dup1	NM_200518_dup1
atad4b	NM_001017705	phox2a	NM_207070	il2rgb	NM_001123050
slc16a12b	NM_001145814	zgc:92869	NM_001030271	otop1	NM_198803
zgc:113062	NM_001017875	paqr7b	NM_183345	spon2b	NM_131008
LOC799537_dup1	NM_001110170_dup1	zgc:172086	NM_001110415	zgc:101740	NM_001004628
plek2	NM_001128739	zgc:172265	NM_001114911	zgc:158328	NM_001080189
rh30	NM_001024819	dio2	NM_212789	scn1ba	NM_001077539
six2.1	NM_131783	vax1	NM_194410	stmn2a	NM_001005923
atoh8	NM_001079991	LOC100005645	NM_001100095	zgc:73062	NM_199959
fgf20b	NM_001039172	zgc:175148	NM_001114458	col10a1	NM_001083827
zgc:114084_dup2	NM_001030213_dup2	clu	NM_200802	rasgrp3	NM_001110464
zgc:136859	NM_001040301	cyp2j29	NM_001082936	fam168a	NM_001045470
il1rapl2	NM_001142584	rgs6	NM_001035263	si:ch211-132b12.1	NM_001083064
samsn1a	NM_001128669	si:ch211-246k22.2	NM_001020662	si:ch211-203b8.6	NM_001128730
zgc:86870_dup1	NM_001002680_dup1	si:dkey-51e6.1	NM_001030119	sparcl	NM_001130605
zgc:92349	NM_001002446	si:dkey-7c18.9	NM_001082898	efna1a	NM_200597
taf12_dup2	NM_198368_dup2	c1qc	NM_001005976	necab1	NM_001017848
tnfrsf1a	NM_213190	capn2b	NM_001018227	psmb8	NM_131392
zgc:91887	NM_001004542	si:ch211-262h13.5	NM_001045212	si:dkeyp-92c9.2	NM_001045220
drd4a	NM_001012616	si:dkey-72g22.1	NM_001110518	zgc:73061	NM_213042
LOC100000800	NM_001159836	slc12a10.2	NM_001045001	amy2a	NM_213011
nkx2.2a	NM_131422	lrrc33	NM_001003775	atp1b3a	NM_131221
parvb	NM_199726	ptrf	NM_001114549	cadm3_dup1	NM_001045246_dup1
zgc:101786	NM_001007398	zgc:153639	NM_001077372	cbln2a	NM_001128688
zgc:193725	NM_001128719	cyb5r2	NM_001045360	flvcr1	NM_001025522

Additional file 2: Uniquely shared gene transcripts

wdr69	NM_001044352
nkd2a	NM_001098197
si:ch211-194e15.5	NM_001083090
si:ch211-206a7.2	NM_001135132
si:dkey-284p5.3	NM_001044842
slc9a3.1	NM_001113473
zgc:153728	NM_001045460
zgc:92739	NM_001002576
ftr11	NM_001110397
gfi1.1	NM_001020776
st6galnac3	NM_212625
tpo	NM_001143753
zgc:113144	NM_001013571
zgc:154006	NM_001080004
zgc:163081	NM_001089512
si:dkey-33i22.2	NM_001030220
zgc:136808	NM_001045298
zgc:101774	NM_001004623
zgc:136885	NM_001105278
zgc:158231	NM_001080179
foxq2	NM_001104941
rdh5	NM_001030101
si:dkey-21e7.2	NM_001100013
si:dkeyp-98a7.5	NM_001160362
zgc:123116	NM_001044930
zgc:171500	NM_001110128
bmp7b	NM_001077146
cacnb3a	NM_001083555
nadl1.2	NM_131361
zgc:172103	NM_001110410
zgc:173927	NM_001110041
ahrra	NM_001035265
vipr2	NM_131779
zgc:91999	NM_001003498
otomp	NM_001045087
tigara	NM_001039836
zgc:92489	NM_001002381
atp2a1	NM_001007029
epor_dup1	NM_001043334_dup1
grap	NM_001020573
prl	NM_181437
zgc:194665	NM_001128746
cyp3c1l2	NM_001007400
LOC100001340	NM_001127520
zgc:110152	NM_001017853
zgc:73293_dup2	NM_213028_dup2
rtn4r	NM_203478
zgc:112282	NM_001020674
chrna1	NM_131445
pfkmb	NM_001089417
zgc:103466	NM_001006028
si:ch211-138a11.5	NM_001123054
tmem88b	NM_001077144
zgc:158677	NM_001082995
zgc:92192	NM_001002340
lgi3	NM_001039679
sult1st3	NM_183348
zgc:194990	NM_001130790
hoxd13a	NM_131169
si:ch211-194d6.1	NM_001045137
nkx2.2b	NM_001007782
six4.1	NM_131717
zgc:136888	NM_001040378
iclp2	NM_131372
tlcd1	NM_001006071
zgc:101809	NM_214717
zgc:73142_dup2	NM_200873_dup2
im:6805837	NM_001118891
LOC571364	NM_001045218
mfap2	NM_001039357
serpinc1	NM_182863
si:ch211-154a22.8	NM_001100440
si:dkey-253d23.4	NM_001110375
atp1b2a	NM_131669
cmyb	NM_131266
igsf21b	NM_001110473
sgk2_dup2	NM_001113622_dup2
zgc:171775	NM_001111167
zgc:65861	NM_199552
met	NM_001007124
pkm2b	NM_001003488
slc17a6l	NM_001009982
tnni2a.1	NM_001007365
tnnt3a	NM_131565
foxj1a	NM_001076706
lgals3bpa	NM_001040041
lgals3bpb	NM_212873
zgc:100868	NM_001003526
zgc:153968	NM_001077456
zgc:77415	NM_207053
zgc:91874	NM_001002188
abcc9	NM_001030154
b2m	NM_001159768
ccdc3	NM_001025510
metap2l	NM_001100054
si:dkey-153k10.9	NM_001045110
kiss1rb	NM_001110531
p2rx1	NM_198982
prlrb	NM_001113500
tnc	NM_130907
cyp24a1l	NM_001089458

Additional file 2: Uniquely shared gene transcripts

rnd2	NM_001045364
zgc:136820	NM_001045859
zgc:173593	NM_001109705
lin7a	NM_200709
nots	NM_131804
si:dkey-61p9.7	NM_001044889
zgc:175035_dup1	NM_001135684_dup1
atp1b2b	NM_131838
chrne	NM_001003772
cx27.5	NM_131811
dab1	NM_001040685
dao.3	NM_205694
dmrt1	NM_205761
dmrt3a	NM_001005779
foxn4	NM_131099
LOC562135	NM_001100949
LOC562468	NM_001126404
LOC797046	NM_001114691
zgc:73336	NM_200794
dmrt2b	NM_001079976
rhcgl1	NM_001109843
sema3fa	NM_001014822
slc6a13l	NM_001122761
tp63	NM_152248
zgc:158252	NM_001080085
cldn15l	NM_200404
hsd11b2	NM_212720
pbx1a	NM_131614
pbx1a	NM_131615
gck	NM_001045385
mapkapk2b	NM_001101803
prr16	NM_001110405
wnt5a	NM_001079834
zgc:158613	NM_001083018
klf12b	NM_001109865
si:ch211-125e6.14	NM_001111241
tmem30c	NM_200596
zgc:123096	NM_001037226
znf385b_dup1	NM_212793_dup1

dytn	NM_001080587
il12rb2	NM_001113505
tp63	NM_152987
wdr82	NM_001009897
zgc:101810	NM_001007772
zgc:110179	NM_001017840
zgc:153219	NM_001076581
dio1	NM_001007283
dusp2	NM_001003451
eps8l3	NM_200361
ildr2	NM_001030192
rdh1l	NM_199609

Additional file 2: Uniquely shared gene transcripts

Additional file 2: Uniquely shared gene transcripts

16cell-512 cell		16cell-50% epiboly		512cell-50% epiboly	
crmp1	NM_001020725	mxa	NM_182942	asb5	NM_001017753
zgc:165343	NM_001080178	zgc:77358	NM_205632	fgf20a	NM_001037103
zgc:165621	NM_001100441	glis3	NM_001080607	gefiltin	NM_131032
camkv	NM_200450	sept5b	NM_001003782	ldb2b	NM_131315
nr0b1	NM_001082947	zgc:111821	NM_001002308	LOC557028	NM_001089374
pax7a	NM_131324	zgc:153423	NM_001077461	LOC563273	NM_001145580
si:dkey-13a21.4	NM_001013478	btf3l4_dup2	NM_200694_dup2	tll1	NM_131010
si:ch211-146f4.4	NM_001044816	LOC100150352	NM_001161401	vegfc	NM_205734
fgfr3	NM_131606	si:busm1-6a2.1_dup2	NM_001075104_dup2	zgc:101052	NM_001003579
ret1	NM_181662	zgc:113184	NM_001020716	zgc:136353	NM_001045275
zgc:153142	NM_001077339	zgc:158773	NM_001082411	otx1l	NM_131215
itln3	NM_001159584	zgc:77375	NM_205631	zgc:136359	NM_001045269
zgc:154020	NM_001077607	zgc:109930	NM_001024436	zgc:172339	NM_001111177
zgc:92608	NM_001002706	zgc:194501	NM_001130780	b3gnt5	NM_198876
abcg4b	NM_001111212	fgfr2_dup3	NM_178303_dup3	ctns	NM_001020571
zgc:77409	NM_205704	osr1	NM_001006079	socs8	NM_001114554
cyp4	NM_199216	pcdh2ab12	NM_001009591	sult6b1	NM_214686
pex5_dup2	NM_201156_dup2	pde6a	NM_001007160	zgc:154141	NM_001077273
sim1	NM_178222	pls3	NM_001002326	aqp8a	NM_001004661
LOC794087	NM_001128782	zgc:92762	NM_001002561	dkk1	NM_131003
pabpc4	NM_201296	cldna	NM_131762	hoxb5b	NM_131537
zgc:154055	NM_001077142	scn3b	NM_001080802	mxtx2	NM_001079816
zgc:175176	NM_001114460	zgc:110585	NM_001017906	slc16a9b	NM_001003552
cyp19a1a	NM_131154	zgc:112083_dup1	NM_001017764_dup1	sult2st1	NM_198914
grk7b	NM_001033090	zgc:123204	NM_001039991	zfhx1	NM_131709
LOC100004247	NM_001144815	zgc:153721	NM_001079680	cxcl12a	NM_178307
si:dkey-246a16.3	NM_001045133	zgc:153898	NM_001077555	fgfr2_dup2	NM_178303_dup2
zgc:158671	NM_001100000	a2bp1l	NM_214775	ism1	NM_001012376
LOC100000030	NM_001100067	ceacam1	NM_001113794	pla2g12b_dup2	NM_213430_dup2
si:ch211-197n10.4	NM_001082901	pklr	NM_201289	tlr4a	NM_001131051
opn4l	NM_178289	zgc:101640	NM_001007444	vent	NM_131700
pbx1a	NM_131615	zgc:101891	NM_001005959	zgc:153073	NM_001077581
zgc:103762	NM_001006007	zgc:172162	NM_001128252	zgc:193505	NM_001128697
kcnh5a	NM_001044798	insm1b	NM_199658	fgf24	NM_182871
prrx1b	NM_200050	si:ch211-151h10.2	NM_001045140	kazald2	NM_001105124
cfb	NM_131338	slc12a3	NM_001045080	pcdh18b	NM_001115099
gsdf	NM_001114668	zgc:103515	NM_001006013	spry1	NM_001122600
trpc6	NM_001030282	asb4	NM_001017628	zic3	NM_001001950
lhx9	NM_001037243	si:dkey-21p19.1	NM_001083084	chd	NM_130973
rgs2	NM_001002662	si:dkeyp-113d7.4	NM_001114342	cldne	NM_131765

Additional file 2: Uniquely shared gene transcripts

si:dkeyp-31e2.1	NM_001082925
zgc:171601	NM_001111234
mip1	NM_001003534
zgc:136569	NM_001045287
drd3_dup2	NM_183067_dup2
gygl	NM_001002062
LOC100141479	NM_001115101
zgc:162127	NM_001159975
cbx8a	NM_205616
rln3	NM_001037803
zgc:109934	NM_001020531
zgc:92419	NM_001002330
si:dkey-21o22.1	NM_001030186
dao.2	NM_214732
plp1b	NM_001005586
vps33a	NM_001099973
zgc:113858	NM_001025490
lingo1b	NM_001004576
mnx1	NM_001009885
mybpc3	NM_001044349
zgc:153148	NM_001076742
zgc:92903	NM_001002461
alas2	NM_131682
foxe3	NM_001079682
si:dkey-24f17.5	NM_001040382
si:dkeyp-38g6.2	NM_001044342
zgc:110354	NM_001020586
hoxd9a	NM_131126
cdc14aa	NM_201149
cldn10l	NM_131771
LOC568508	NM_001159830
ripk1l	NM_001043350
allc	NM_001002716
rd3	NM_001080631
si:dkey-239i20.2	NM_001044829
cel.1	NM_199607
cldnc	NM_131764
pitx1	NM_001040346
slc45a2	NM_001110377
zgc:112359	NM_001111214
zgc:55420	NM_213507
hspb7	NM_001006040
LOC563864	NM_001110521
rx1	NM_131225
si:dkey-205o12.6	NM_001080804
slit1b	NM_001034975
LOC553461	NM_001159639
zgc:175128	NM_001114453
zgc:73155	NM_200829
zgc:86915	NM_001002092
zgc:112054	NM_001017664
zgc:172142	NM_001114565
dkk3	NM_001089545
cyp2k6	NM_200509
eps8l1	NM_001013313
fzd2	NM_131140
gsg1l	NM_001039976
hsp70_dup2	NM_131397_dup2
slc35b1	NM_001004583
slc5a11	NM_001007300
timp2b	NM_213296
zgc:66268	NM_200512
chpt1	NM_212624
tnnt1	NM_181499
zgc:153948	NM_001077276
zgc:158179_dup1	NM_001083814_dup1
zgc:86661	NM_001002059
gcnt4	NM_201583
mboat4	NM_001122944
dharma	NM_130979
gip	NM_001100144
kirrel3	NM_001099344
mab21l1	NM_152974
nnr	NM_001029947
sag	NM_001033749
vtna	NM_001020672
zgc:101797	NM_001007397
zgc:171582	NM_001111210
enpp2	NM_200603
pou50	NM_131161
tnfaip8l	NM_200374
zgc:161979	NM_001045488
qk_dup1	NM_131224_dup1
zgc:113886	NM_001030079_dup1
zgc:113886	NM_001030079_dup2
zgc:153973	NM_001077547
zgc:77799	NM_200868
foxa3	NM_131299
has3	NM_173220
lmo2	NM_131111
si:dkey-270i2.2	NM_001044883
agr2	NM_001012481
cki	NM_131108
mhc1uba	NM_131471
rab25	NM_001008641
si:dkeyp-113d7.7	NM_001083105
spag1	NM_001089406
zgc:92533	NM_001003445
cahz	NM_131110
gng12	NM_213312
zgc:172106	NM_001114563
epha7	NM_001044979
igfbp1a	NM_173283
jun	NM_199987
lnx1	NM_001075113
LOC556137	NM_001044796
moxd1l	NM_001128525
si:ch211-261f7.2	NM_001025550
si:dkey-25e12.3	NM_001025555
tagap	NM_001004548

Additional file 2: Uniquely shared gene transcripts

naaladl1	NM_001098190	uts1	NM_001030180
npr3	NM_001102673	adora2a.2	NM_001040036
si:ch211-241j12.1	NM_001080028	fgfr4	NM_131430
si:ch211-255a21.1	NM_001126431	gria1b	NM_205730
si:rp71-10d23.3	NM_001089376	lhx5	NM_131218
zgc:136377	NM_001045255	slc26a1	NM_001080667
zgc:194158	NM_001128754	zgc:85869	NM_213226
caspxa	NM_001083862	tmcc2	NM_001044919
gad1	NM_194419	zgc:100942	NM_001003626
quo	NM_001004109	chl1	NM_001163106
zgc:152917	NM_001077369	gata5	NM_131235
zgc:162630	NM_001082825	hnf4a	NM_194368
zgc:172082	NM_001111229	sp5l	NM_194371
zgc:92105	NM_001004547	zgc:101673	NM_001006080
zgc:92231	NM_001004584	zgc:153784	NM_001080055
f2	NM_213390	plcd1a	NM_001109700
htr5al	NM_001007121	zgc:112515	NM_001017762
pax6b	NM_131641	foxb1.2	NM_131285
zgc:158632	NM_001080678	irx3b	NM_213100
LOC100002223	NM_001126472	kctd15l	NM_001006012
LOC560297	NM_001126396	zgc:113571	NM_001013512
tas2r202	NM_001100629	eve1	NM_131114
znf131	NM_200505	notch3	NM_131549
si:dkey-11f4.16	NM_001098254	wu:fb01b03	NM_001142583
		zgc:114118	NM_001030202
		zgc:123295	NM_001037562
		zgc:162495	NM_001089551
		zgc:172253	NM_001114483
		zgc:174646	NM_001122615
		epyc	NM_001017903
		net1	NM_001007767
		prickle1b	NM_001030098
		si:dkey-14d8.5	NM_001044841
		si:dkey-14d8.6	NM_001044839
		vegfab	NM_001044855
		zgc:163027	NM_001089333
		zgc:174708	NM_001114900
		zgc:85975	NM_213265
		ctsll	NM_001005999
		efnb1	NM_131805
		LOC564882	NM_001105113

Additional file 2: Uniquely shared gene transcripts

		LOC567472	NM_001128541
		si:ch211-89p1.1	NM_001082982
		si:dkey-252h13.6	NM_001075103
		tagln2	NM_201576
		zgc:136538_dup1	NM_001045237_dup1
		zgc:136538_dup2	NM_001045237_dup2
		zgc:171581	NM_001114586
		foxd3	NM_131290
		klfd	NM_130936
		lrrc15	NM_001080682
		sb:cb742	NM_001118897
		slco2a1	NM_001089582
		zgc:101788	NM_001105601
		zgc:153063	NM_001076770
		zgc:153702	NM_001076592
		zgc:165344_dup1	NM_001100137_dup1
		alox12	NM_199618
		plekhf1	NM_200340
		aplnra	NM_001075105
		fgf17	NM_214808
		foxd5	NM_131270
		her3	NM_131080
		hnf1a	NM_170764
		zgc:77806	NM_205691
		asb11	NM_001045313
		asb11	NM_214792
		frzb	NM_130943
		ghrhr	NM_001131052
		has2	NM_153650
		lcp1	NM_131320
		stat1b	NM_200091
