# Supplementary figures and images for: The zebrafish transcriptome during early development
Source: BMC Dev Biol. 2011 May 24;11:30. doi: 10.1186/1471-213X-11-30 (PMC3118190; doi:10.1186/1471-213X-11-30)

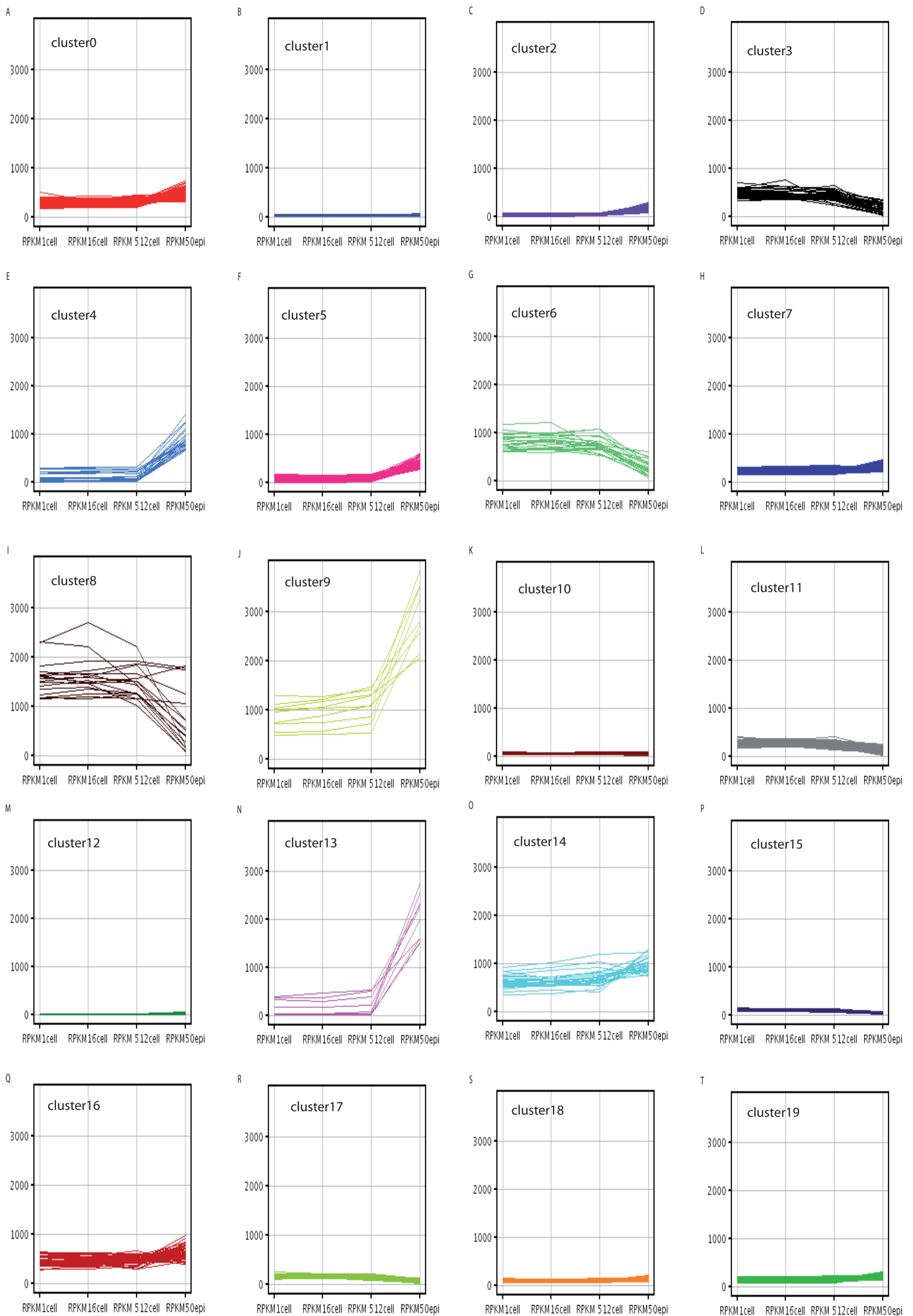

Supplement: Additional file 3 — Expression profile clustering of detected transcripts. Genes with a high degree of similarity in expression patterns across the four developmental stages RPKM values were clustered using K-means method via GeneSpring (Agilent Technologies). The number of clusters was fixed to 20 and similarity was evaluated by Euclidean measurement. The maximum number of iterations was fixed to 50. [file 1471-213X-11-30-S3.PDF]

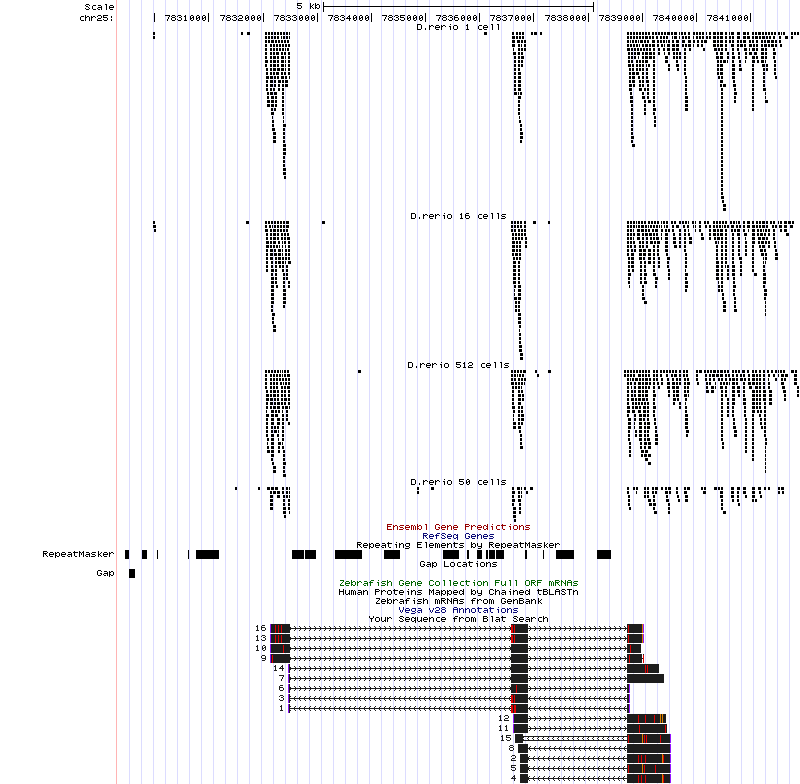

Supplement: Additional file 8 — Putative novel transcribed region identified from the RNA-Seq. Validation of a putative novel transcribed region. The illustration shows selected tracks from the UCSC viewer http://genome.ucsc.edu in a region on chromosome 25 that lacks annotated transcripts. The four upper tracks show the mapped RNA-Seq reads for each developmental stage, in which the exon structures can be identified via the piled-up reads. The bottom track shows the results of a blat alignment of the sequenced PCR products to the genome. The PCR products and RNA-Seq reads indicate similar exon structures. [file 1471-213X-11-30-S8.PNG]

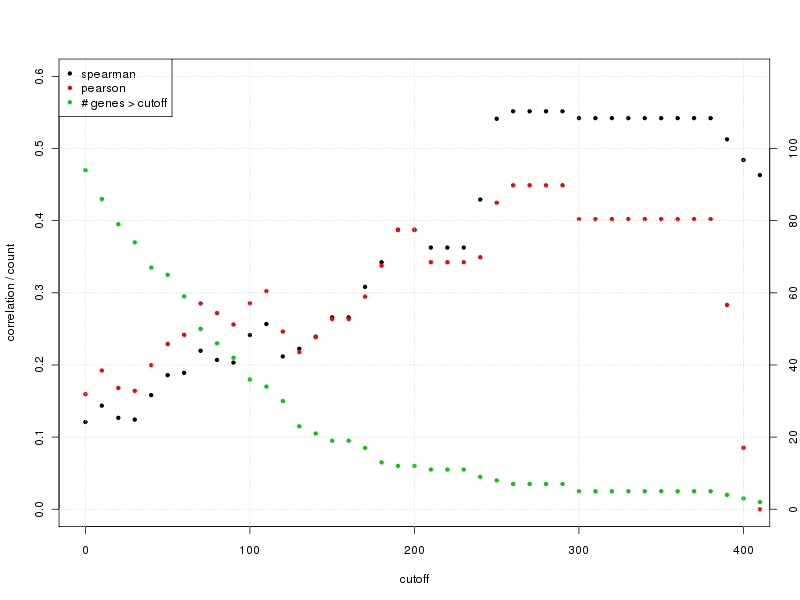

Supplement: Additional file 10 — Correlation plot between previous reported pre-MBT accumulated transcripts and transcripts detected in the present study. Plot showing the correlation between Mathavan et al. (2005) microarray data and RNA-Seq RPKM values from the present study. The correlations are based on the comparisons of stages 4 cell - 1 cell, 64 cell - 16 cell, and 6 hpf - 50% epiboly, where the first stage in each pair corresponds to the average expression value in the Mathavan et al. (2005) data set. [file 1471-213X-11-30-S10.TIFF]

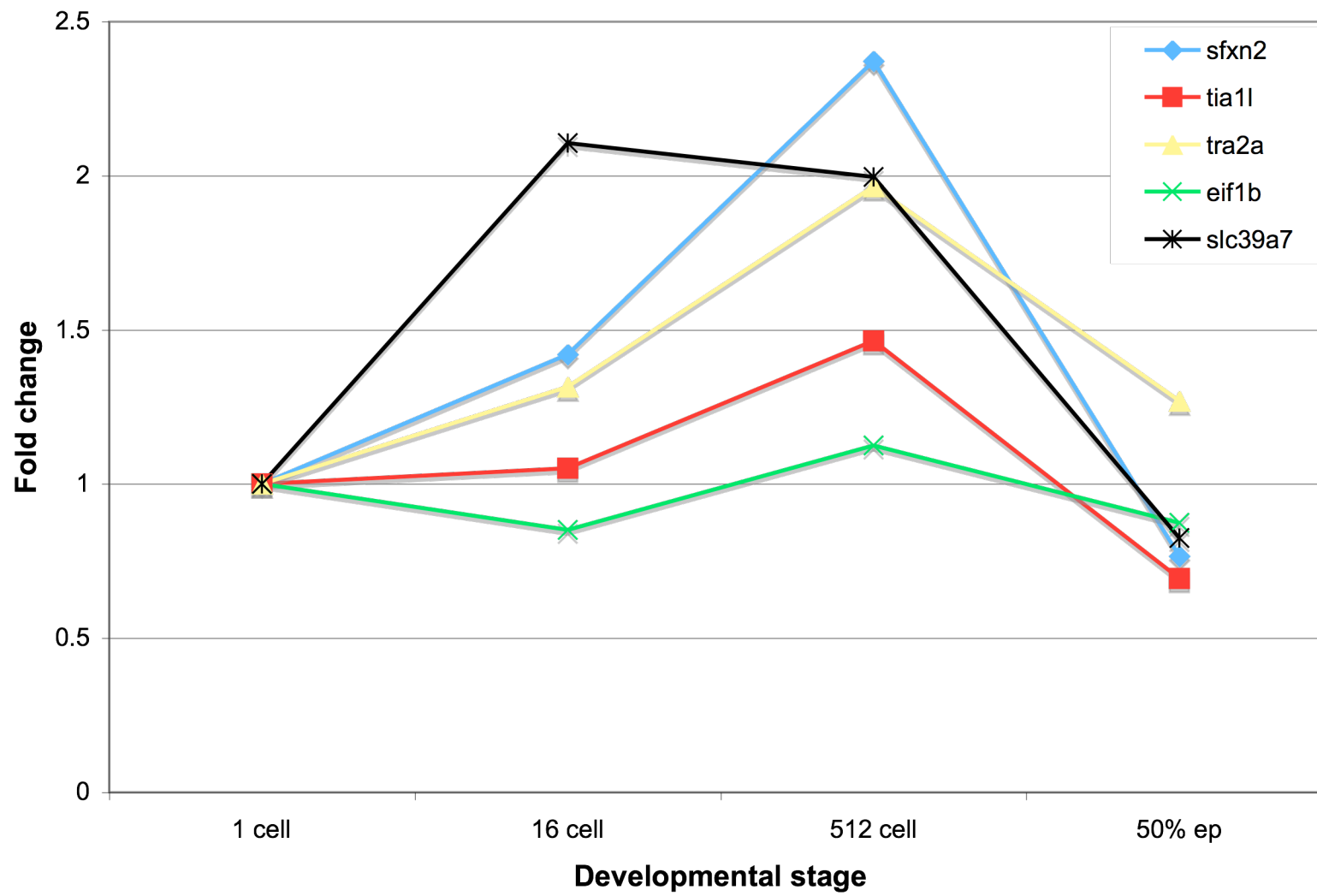

Supplement: Additional file 11 — Validation of pre-MBT accumulation of gene transcripts using semi-quantitative RT-PCR. Mathavan et al. (2005) reported a pre-MBT accumulation of eif1b, tra2a, tia1l and sfxn2, which could not be reliably validated in this analysis. The zinc transporter gene transcript Slc39a7 was detected as increasing between 1-cell stage and 16-cell stage in the RNA-Seq dataset. The semi-quantitative RT-PCR expressional analysis confirms the pre-MBT accumulation of slc39a7. The analysis was made using duplicate samples of biological triplicates from each developmental stage. [file 1471-213X-11-30-S11.PDF]

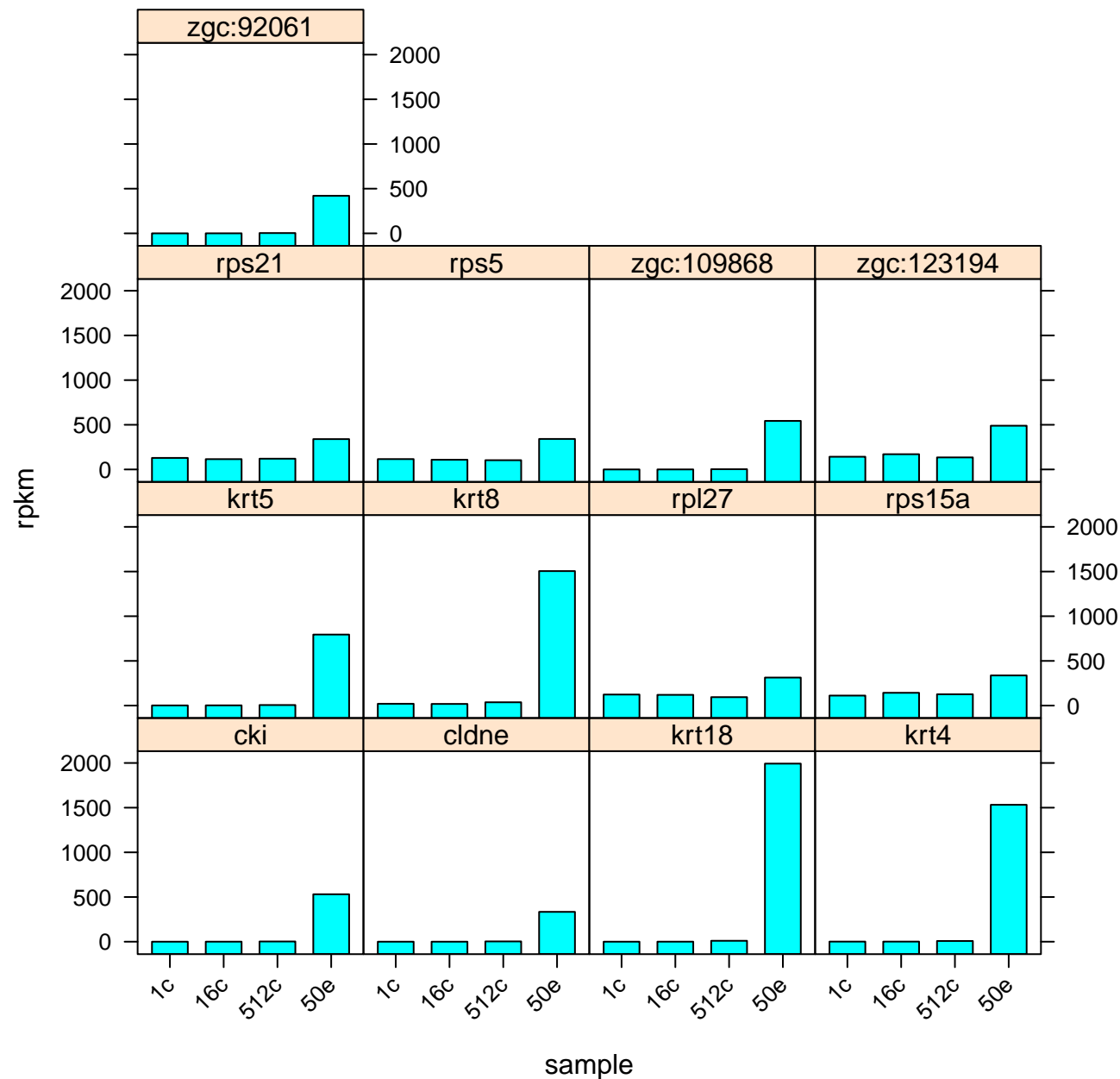

Supplement: Additional file 12 — Bar-plot of structural gene transcript expression during development. Bar-plot showing the expression profile of a number of structural gene transcripts in the four developmental stages studied. The different stages are indicated using following legends: 1c - 1-cell stage; 16 c - 16-cell stage; 512 c - 512-cell stage and 50 e - 50% epiboly. [file 1471-213X-11-30-S12.PDF]

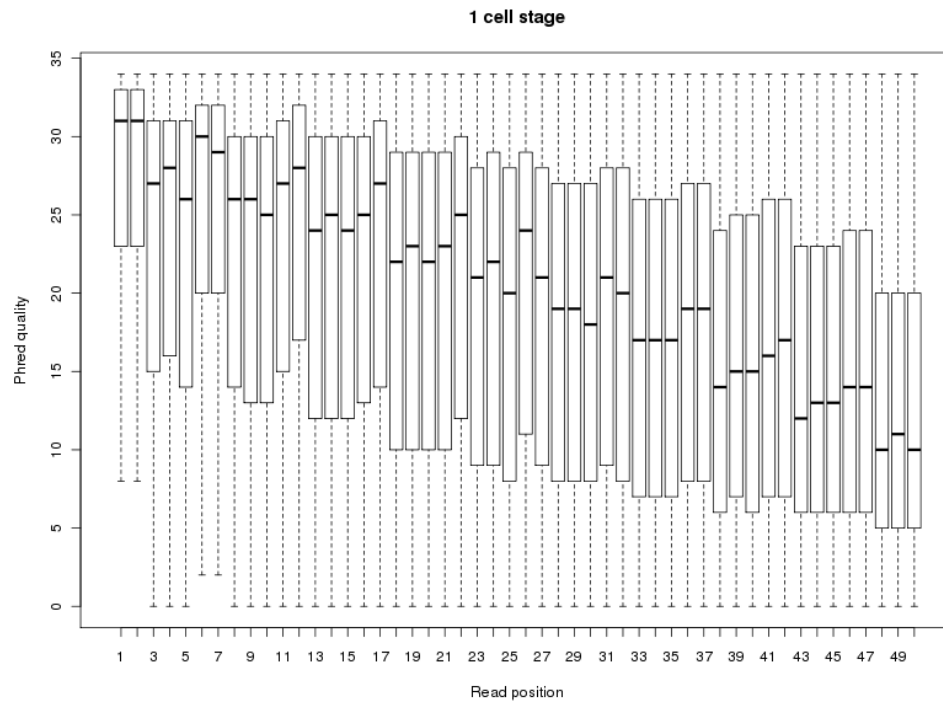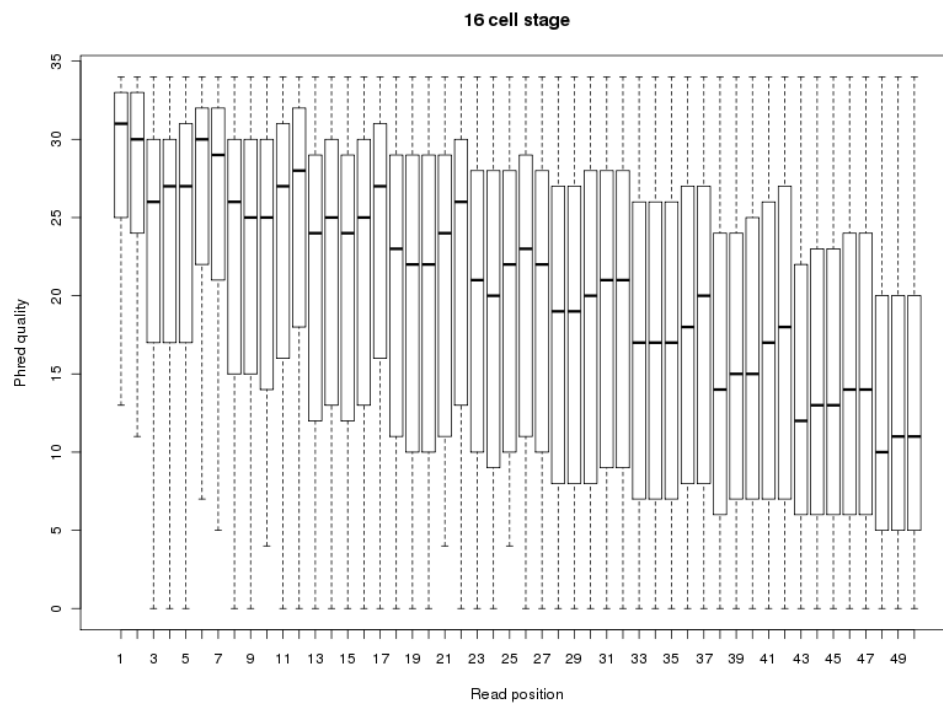

512 cell stage

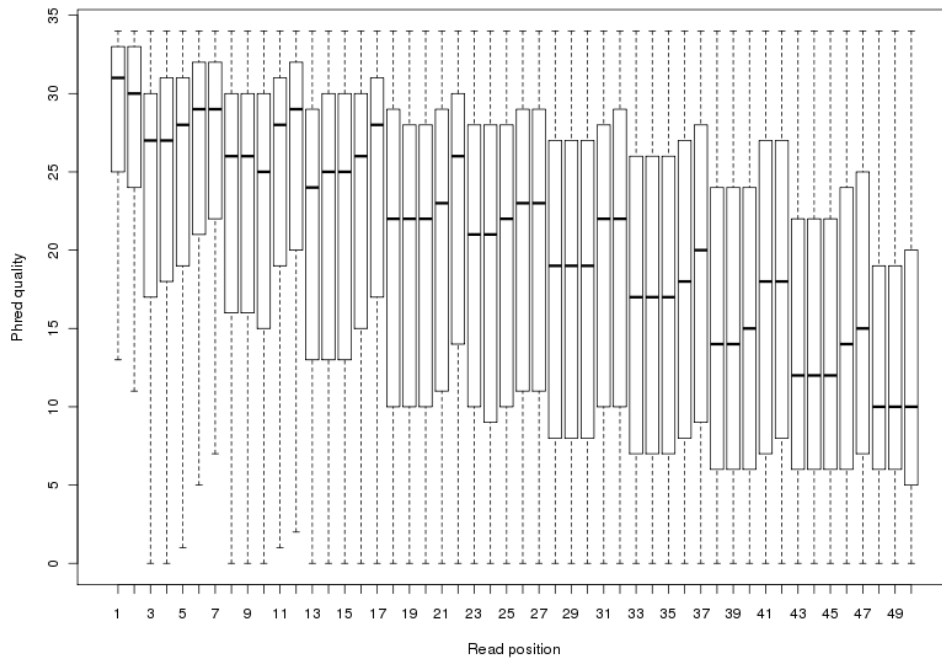

50% epiboly stage

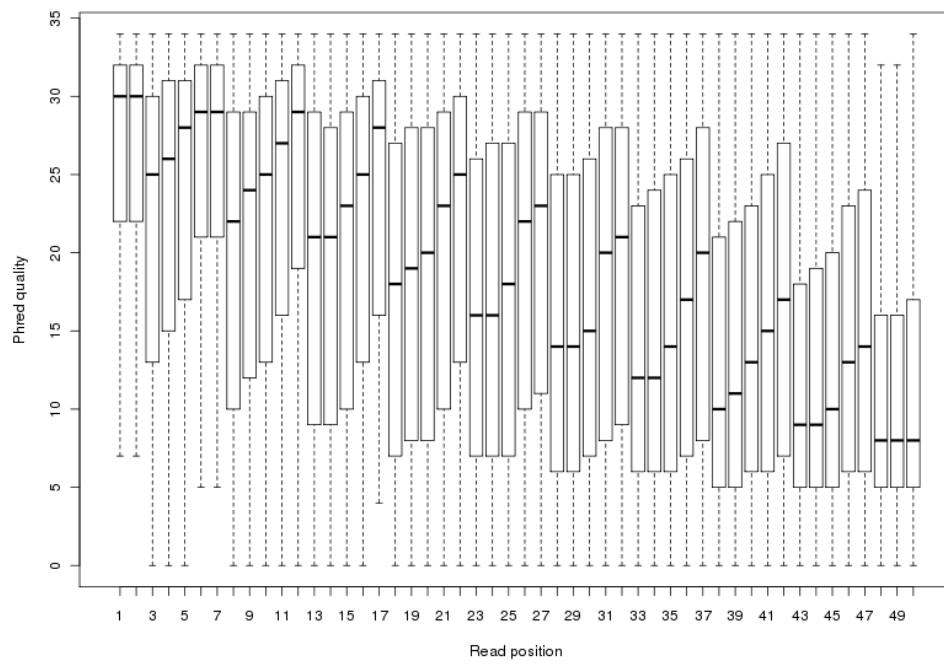

Supplement: Additional file 14 — Sequence quality for the different developmental stages studied. Box plot of phred quality value distributions by read position using FASTX toolkit to assess the sequencing quality. The different stages are indicated using following legends: 1c - 1-cell stage; 16 c - 16-cell stage; 512 c - 512-cell stage and 50 e - 50% epiboly. [file 1471-213X-11-30-S14.PDF]
