# Supplementary material for: The zebrafish transcriptome during early development
Source: BMC Dev Biol. 2011 May 24;11:30. doi: 10.1186/1471-213X-11-30 (PMC3118190; doi:10.1186/1471-213X-11-30)
Supplement: Additional file 4 — Gene transcripts present in the expression profile clusters. Expression profile clustering information for the detected gene transcripts. [file 1471-213X-11-30-S4.PDF]

Gene symbol	Cluster number
zgc:163038	0
zgc:73265	0
zgc:92237	0
cldn7	0
etf1	0
mat2a	0
tmed2	0
ddx19	0
top1l	0
zgc:171710	0
zgc:171772	0
zgc:174506	0
zgc:66127_dup1	0
psme3	0
psap	0
rps24	0
si:ch211-198a12.6	0
ccng1	0
ctnna_dup1	0
ctnna_dup2	0
fam60al	0
zgc:56330	0
ctsc	0
ywhae1	0
cited3	0
ctnnb1	0
fbl	0
pabpc1a	0
sec61b	0
zgc:110525	0
zgc:92872	0
eif2s1l	0
npc2	0
rpl13a	0
arf5_dup2	0
eif4g2b	0
mapk6	0
rpl4	0
ctnnb2	0
hdac1	0
khdrbs1b	0
rpl11	0
rpl15	0
tra2a	0
zgc:55292	0

zgc:91910	0
elavl1	0
rpl7	0
rps28	0
zgc:77429	0
erh	0
rplp1	0
sox11b	0
xrn2	0
dkc1	0
rpl23a	0
zgc:77767	0
oaz1	0
sfrs3b	0
si:dkey-42i9.4	0
uhrf1	0
zgc:136952	0
hmgb2l	0
sfrs6a	0
eif5a	0
hsd17b12a	0
zgc:173585	0
ddx39b	0
gspt1l	0
zgc:92066	0
nap1l1	0
rpl18a	0
si:ch211-51e12.7	0
aif1l	0
ewsr1b	0
f11r	0
ranbp1	0
rplp0	0
tia1l	0
vcp	0
zgc:77155	0
glulb	0
rpl5b	0
sec61al1	0
serbp1	0
zgc:86706	0
dnaja2	0
fam60al_dup2	0
zgc:101832	0
rpl10a	0
rpl6	0

seta	0
rpl8	0
sap18	0
acy3.1	1
agpat3	1
ankrd37	1
appa	1
aptx	1
arl6	1
atp5j	1
b3gntl1	1
cdc37	1
chtf18	1
clgn	1
clock3	1
creb1_dup2	1
ctnna2	1
dpcd	1
dZ63M10.3	1
fam134a	1
gab1	1
galnt7	1
gne	1
hadh	1
hhip	1
itsn1	1
jam2	1
lap3	1
LOC100003587	1
LOC407614	1
LOC449616	1
LOC554386	1
LOC555790	1
LOC558857	1
LOC559710	1
LOC563863	1
LOC571795	1
lrpap1	1
msxb	1
nup133	1
pi4k2a	1
ppm1k	1
pspc1	1
rcbtb1	1
scoc	1
setdb2	1

sh3gl2	1
si:ch73-131e21.7	1
slc2a15b	1
tnrc5	1
ufsp2	1
vps37a	1
zgc:100913	1
zgc:101017	1
zgc:103414	1
zgc:103440	1
zgc:103570	1
zgc:103736	1
zgc:109898	1
zgc:110091	1
zgc:110196	1
zgc:110248	1
zgc:110323	1
zgc:110461	1
zgc:110639	1
zgc:111880	1
zgc:112136	1
zgc:112393	1
zgc:113078	1
zgc:123172	1
zgc:123274	1
zgc:136845	1
zgc:136864	1
zgc:152851	1
zgc:153156	1
zgc:153286	1
zgc:153957	1
zgc:154081	1
zgc:158312	1
zgc:158441	1
zgc:158710	1
zgc:162351	1
zgc:162591	1
zgc:162946	1
zgc:165595	1
zgc:171472	1
zgc:171747	1
zgc:171777	1
zgc:172007	1
zgc:172295	1
zgc:174890	1
zgc:195220	1

zgc:56292	1
zgc:64174	1
zgc:64227	1
zgc:65781	1
zgc:66455	1
zgc:73228	1
zgc:77005	1
zgc:77820	1
zgc:91844	1
zgc:91951	1
zgc:92601	1
znf330	1
akt2l	1
anxa3b	1
auh	1
bnip3l	1
cry5	1
dusp11	1
dyrk1aa	1
fam136a	1
fhl	1
hdhd2_dup1	1
hdhd2_dup2	1
hnrnpd	1
il6st	1
kctd9	1
LOC556956	1
mrpl48	1
mrps24	1
mrrf	1
msi2a	1
nipbl	1
nrarpa	1
orai1	1
pitpnb_dup1	1
pitpnb_dup2	1
polm	1
por	1
ppwd1	1
rad51c	1
rbm18	1
rxfp2	1
sec31a	1
sgta	1
si:rp71-56i13.6	1
slc20a1b	1

snrnp35	1
snx30_dup2	1
sppl3	1
stambp	1
tln1	1
tmem120b	1
tpm2	1
trim3b	1
ube2r2	1
ufm1	1
usp25	1
vps26bl	1
vps36	1
wdr45	1
wdr74	1
xpo7	1
zgc:100918	1
zgc:101696	1
zgc:103664	1
zgc:103754	1
zgc:110006	1
zgc:110246	1
zgc:110281	1
zgc:112045	1
zgc:112329	1
zgc:114136	1
zgc:123019	1
zgc:152924	1
zgc:152984	1
zgc:158234	1
zgc:158416	1
zgc:162918	1
zgc:162979	1
zgc:165597	1
zgc:173602	1
zgc:56235	1
zgc:56368	1
zgc:86773	1
zgc:91931	1
zgc:92106	1
zgc:92243	1
atxn7l2a	1
bxdc5	1
cflar	1
dynlt3	1
gnl3l	1

gpx1a	1
gtpbp3	1
iars	1
ift52	1
lims1	1
magi1	1
mbd1	1
mfn1	1
mrpl45	1
nek4	1
pfkfb4	1
rab7	1
rbm34	1
rem1	1
rpn1	1
slc11a2	1
slc25a26	1
sort1a	1
sp2	1
spcs1	1
srpk1b	1
tada3l	1
tax1bp3	1
tk1	1
wdr51a_dup1	1
wdr51a_dup2	1
zgc:100909	1
zgc:100947	1
zgc:101127	1
zgc:110034	1
zgc:110388	1
zgc:112364	1
zgc:113114	1
zgc:123075	1
zgc:136367	1
zgc:136566	1
zgc:153365	1
zgc:153457	1
zgc:153788	1
zgc:158388	1
zgc:158644_dup2	1
zgc:158648	1
zgc:162873	1
zgc:163064	1
zgc:171488	1
zgc:171818	1

zgc:55557	1
zgc:55764	1
zgc:63485	1
zgc:63749	1
zgc:85644	1
zgc:92006	1
zgc:92107	1
zgc:92127	1
arhgap12	1
bmpr1ab	1
bms1l	1
ca10a	1
capg	1
cby1	1
dcxr	1
galk1	1
gbl	1
ghdcl	1
ghitm_dup1	1
im:7138264	1
lipf	1
LOC559344	1
maff	1
mapk7	1
med29	1
mgrn1_dup2	1
minpp1	1
mpp7	1
noc3l	1
pppde2b	1
psmd11b	1
rangap1	1
rhoq_dup2	1
rnf40	1
socs3b	1
stat5.2	1
tef	1
tubgcp2	1
unk	1
uros	1
vti1a	1
zgc:101094	1
zgc:110306	1
zgc:112254	1
zgc:114107	1
zgc:136865	1

zgc:152869	1
zgc:152875	1
zgc:153060	1
zgc:153358	1
zgc:153609	1
zgc:154039	1
zgc:154088	1
zgc:158759	1
zgc:162204	1
zgc:162976	1
zgc:163016	1
zgc:163044	1
zgc:163107	1
zgc:171487	1
zgc:171978	1
zgc:192851	1
zgc:55344	1
zgc:64213	1
zgc:76887	1
zgc:76924	1
zgc:85611	1
zgc:92181	1
zgc:92822	1
zgc:92926	1
actr2b	1
adka	1
agpat4	1
anxa11a	1
ap3m1	1
atp5s	1
bsdc1	1
cox15	1
dact2	1
elovl6l	1
fancl	1
fbxw4	1
glud1a	1
hps1	1
kbp	1
klc1	1
kop	1
lgi1a	1
LOC560826	1
LOC563246	1
LOC564367	1
LOC566435_dup1	1

LOC568554	1
lrpprc	1
lycat	1
manba	1
map4k5	1
metap1	1
mkks	1
nvl	1
pacs2	1
pdcd2	1
pdss2	1
polr1c	1
pp2ca2	1
rab1a	1
rgs17	1
rock2a	1
rtn4b	1
samm50l	1
sar1a	1
sfxn4	1
si:ch211-101n13.9	1
si:ch211-206k20.5	1
si:ch211-221n23.1	1
si:ch211-67n3.1	1
si:ch211-67n3.4	1
smek2	1
snapc1b	1
tasp1	1
tdrd9l	1
tmem39b	1
tp53bp2	1
tysnd1	1
vrk2	1
wdr92	1
wu:fc08a03	1
zfyve21	1
zgc:100846	1
zgc:101006	1
zgc:101581	1
zgc:101650	1
zgc:103568	1
zgc:109969	1
zgc:111878	1
zgc:112945	1
zgc:113068	1
zgc:113201	1

zgc:136497	1
zgc:136881	1
zgc:153077	1
zgc:158219	1
zgc:162148_dup1	1
zgc:162966	1
zgc:163030	1
zgc:163080	1
zgc:171356	1
zgc:172209	1
zgc:56251	1
zgc:65960_dup1	1
zgc:66426	1
zgc:73311	1
zgc:77529	1
zgc:77775	1
zgc:86834	1
zgc:92087	1
zgc:92598	1
znf503	1
znf511	1
acsl4a	1
aga	1
arl13a	1
atp7a	1
c1galt1c1	1
clint1	1
dlg3	1
dpf2l	1
ef1	1
fibp	1
fmr1	1
foxa	1
gla	1
gpr137bb	1
ints10	1
LOC572060	1
med12_dup1	1
mgat4b	1
mgst2	1
mif	1
mospd1	1
mtmr7a	1
nr3c1	1
nudcd2	1
ogt	1

ppid	1
prom1a	1
rab28	1
rgs14	1
rhoga	1
selt2	1
si:dkey-147f20.5	1
slc9a6a	1
snx25	1
sybl1	1
tcerg1	1
timm8a	1
tmem107	1
tnip1	1
ubxn1	1
upf3b	1
xiap	1
zbtb33	1
zgc:101060	1
zgc:101132	1
zgc:101583	1
zgc:103433	1
zgc:109991	1
zgc:110010	1
zgc:110652	1
zgc:110791	1
zgc:110843_dup1	1
zgc:112326	1
zgc:113138	1
zgc:114080	1
zgc:114085	1
zgc:136929	1
zgc:152683	1
zgc:153168	1
zgc:153361	1
zgc:153681	1
zgc:153710	1
zgc:154054_dup1	1
zgc:154054_dup2	1
zgc:158284	1
zgc:158411	1
zgc:162240	1
zgc:172215	1
zgc:194246	1
zgc:56197	1
zgc:63631	1

zgc:92045	1
zgc:92353	1
znf711	1
acad8	1
actn4	1
aldh3a2	1
bat3	1
bcas3	1
blmh	1
cbl	1
cul5	1
fkrp	1
gbas	1
glod4	1
hmbsb	1
ift80	1
im:7140576	1
lhx1a	1
LOC563525	1
mll	1
mre11a	1
nxn	1
pcsk7	1
rabgef1l	1
sbds	1
slc46a1	1
tfdp2	1
thyn1	1
tmem199	1
tpst1	1
tsr1	1
tubd1	1
vezf1	1
wdfy1_dup2	1
wu:fj53g11	1
zgc:101015	1
zgc:101719	1
zgc:110292	1
zgc:110333	1
zgc:110718	1
zgc:112183	1
zgc:112414	1
zgc:112998	1
zgc:113969	1
zgc:123272	1
zgc:152785	1

zgc:153218	1
zgc:153425	1
zgc:153893	1
zgc:154038	1
zgc:154057	1
zgc:158605	1
zgc:162306_dup1	1
zgc:162698	1
zgc:165514	1
zgc:172049	1
zgc:174574_dup1	1
zgc:55491	1
zgc:55621	1
zgc:56117	1
zgc:63480	1
zgc:63938	1
zgc:66024	1
zgc:85700	1
zgc:92781	1
alg2	1
brd2b	1
casp2	1
cldn12	1
cyb5r4	1
dpy19l1l	1
efna1	1
fam8a1	1
fbxl4	1
gtpbp10	1
LOC563343	1
mtx1a	1
ndufb9	1
phactr4	1
pi4kb	1
plekha8	1
polr3c	1
ptk2.1	1
rhbdl2	1
rpz2	1
rundc3b	1
rxrbb	1
selenbp1	1
si:ch211-154o6.2	1
si:ch211-225p5.3	1
snrk	1
stx17	1

tax1bp1b	1
tfpt	1
tgfbr2	1
tmem103	1
trim32	1
ttc35_dup2	1
tubb5	1
wu:fb25h12	1
wu:fd10a06	1
zdhhc3	1
zgc:100908	1
zgc:101119	1
zgc:101716	1
zgc:101798	1
zgc:103481	1
zgc:110844	1
zgc:112079	1
zgc:113036	1
zgc:113313	1
zgc:114140_dup1	1
zgc:114140_dup2	1
zgc:136493	1
zgc:136982	1
zgc:153328	1
zgc:153966	1
zgc:153995	1
zgc:154073	1
zgc:158262	1
zgc:158455	1
zgc:158642	1
zgc:162701	1
zgc:165655	1
zgc:171438	1
zgc:171680	1
zgc:194590	1
zgc:194596	1
zgc:55908	1
zgc:56388	1
zgc:63907	1
zgc:66117_dup1	1
zgc:66117_dup2	1
zgc:77056	1
zgc:77312	1
zgc:91853	1
zgc:91890	1
zgc:92520	1

zgc:92606	1
abhd12	1
abhd2a	1
adam17a	1
adck1	1
asap2a	1
atg10	1
bcmo1	1
bre	1
cnksr1	1
ctage5	1
ddefl1	1
dnajc17	1
emilin1b	1
ero1l	1
evla	1
flj20272l	1
fuca2	1
fyna	1
hmbox1	1
iah1	1
iars2	1
im:7162391	1
itgb1bp1	1
ivd	1
LOC569212	1
lrrc57	1
mdh1b	1
mpp5a	1
nenf	1
nipal3	1
otx1	1
pank1a	1
pdcd7	1
ppp2r3c	1
pygb	1
qrsl1	1
setd3	1
si:ch211-175f11.2	1
sly1	1
smyd3	1
snx6	1
spdya	1
srp54	1
supt3h	1
supt7l	1

tfb2m	1
tmem167a	1
tmem30a	1
ttc8	1
tulip1	1
vps39	1
zgc:100795	1
zgc:100927	1
zgc:103738	1
zgc:110265	1
zgc:110391	1
zgc:110783	1
zgc:110805	1
zgc:112072	1
zgc:112153	1
zgc:112267	1
zgc:112365	1
zgc:112487	1
zgc:113338	1
zgc:113944	1
zgc:114067	1
zgc:152894	1
zgc:153222	1
zgc:153297	1
zgc:153379	1
zgc:153460	1
zgc:153612	1
zgc:154061	1
zgc:158327	1
zgc:158466_dup2	1
zgc:162183	1
zgc:162964	1
zgc:162968	1
zgc:171644	1
zgc:171667	1
zgc:171690	1
zgc:194204	1
zgc:194556	1
zgc:63689	1
zgc:65997	1
zgc:66313	1
zgc:91976	1
zgc:92683	1
agk	1
bida	1
cd151l_dup1	1

cog8	1
cpsf5	1
ets1a	1
gas8	1
golim4a	1
gro2	1
hnrnpul1	1
igf1ra	1
kbtbd4	1
LOC555838	1
LOC565175	1
LOC571606	1
lonp2	1
nucb2b	1
nudt4	1
nup205	1
pak1	1
paqr5a	1
pard6a	1
pid1	1
ppp1r13l	1
pskh1	1
ptplad1	1
rrp15	1
sema3e	1
si:busm1-241h12.4	1
si:ch211-15i6.2	1
si:ch211-196l7.3	1
si:ch211-212o1.2	1
si:ch211-216l23.1	1
si:ch211-216l23.2	1
si:ch211-234g24.1	1
si:ch211-257c9.1	1
si:dkey-102f14.5	1
si:dkey-103i16.1	1
si:dkey-24l11.4	1
si:dkey-7l12.4	1
si:dkeyp-1h4.2	1
si:dkeyp-84g1.1	1
siae	1
slc33a1	1
st8sia2	1
synbl	1
tiparp	1
trabd	1
ube2q1	1

vasp	1
wash	1
zgc:111960	1
zgc:112052	1
zgc:112097	1
zgc:112101	1
zgc:112172	1
zgc:112432	1
zgc:113340	1
zgc:113920	1
zgc:114135	1
zgc:153239_dup1	1
zgc:153294	1
zgc:153569	1
zgc:158718	1
zgc:171915	1
zgc:77202	1
zgc:85963	1
zgc:92878	1
znf592	1
abcf1	1
atp6v1c1l	1
atp9b	1
bat1	1
brd9	1
brp16	1
ccdc126	1
cno	1
cpsf1	1
daxx	1
dpm3	1
flot1	1
grb10	1
gtf2h4	1
hibadha	1
hsd17b8	1
hsf1	1
ing4	1
krit1	1
lass2	1
LOC799904	1
mpp6	1
mrpl24	1
msto1	1
mterfd1	1
mtx1b	1

nfe2l3_dup2	1
nkiras1	1
ppp1r10	1
psmd3	1
rprd2	1
rps18	1
rrm2b	1
sdc2_dup1	1
si:ch211-13c6.2	1
si:ch211-13c6.3	1
si:ch211-222e23.8	1
si:ch211-254e15.2	1
si:ch211-282e16.1	1
si:ch211-287b5.3	1
si:ch211-81a5.7	1
si:dkey-158b13.2	1
si:dkey-190l1.1	1
si:dkey-218l8.1	1
si:dkey-218n20.1	1
si:dkey-222b8.2	1
si:dkey-46a12.1	1
si:dkeyp-113d7.1	1
si:dkeyp-114f9.2	1
si:dkeyp-34f6.5	1
si:dkeyp-46h3.2	1
si:dkeyp-66g8.1	1
si:dkeyp-84f11.5	1
si:dkeyp-89d7.1	1
si:rp71-45k5.2	1
skiv2l	1
slc25a32a	1
tbc1d5	1
tomm40l	1
triqk	1
trnau1ap	1
vars2	1
zfpm2b	1
zgc:100998	1
zgc:103448	1
zgc:103488	1
zgc:103761	1
zgc:110561	1
zgc:111823	1
zgc:111986	1
zgc:153371	1
zgc:153389	1

zgc:153436	1
zgc:158297	1
zgc:163126	1
zgc:165522	1
zgc:171663	1
zgc:175136	1
zgc:175173	1
zgc:56305	1
zgc:63863	1
zgc:73210	1
zgc:73380_dup1	1
zgc:73380_dup2	1
zgc:77395	1
zgc:92308	1
adhfe1	1
arhgef11	1
arpc5a	1
chico	1
d2hgdh	1
dlg1l	1
dpyd	1
efr3a	1
eif4a2	1
epdr1	1
fam69a	1
fam73a	1
fggy	1
ftr24	1
fzd8b	1
glmna	1
gng7	1
hccsa	1
hectd3	1
hs2st1	1
ift57	1
kdsr	1
kifap3	1
LOC100149582	1
LOC100150696	1
LOC557127	1
LOC558213	1
lyrm4	1
mycb	1
nadk	1
nr2f6a	1
nsun4	1

pak2a	1
pgpep1	1
pik3r2	1
pld1a	1
pter	1
rpap2	1
scp2	1
si:ch211-12e1.4	1
si:ch211-241e1.5	1
si:ch211-284b7.3	1
si:ch211-284g18.3	1
si:dkey-181m9.10	1
si:dkey-216e9.4	1
si:dkeyp-35b8.5	1
smg7	1
st3gal3l	1
stap2a	1
stard3nl	1
tmem68_dup1	1
tmem68_dup2	1
ube2w	1
ubtfl	1
urod	1
xrp1	1
zgc:101828	1
zgc:110183	1
zgc:110343_dup2	1
zgc:110537	1
zgc:110542	1
zgc:110602	1
zgc:111879	1
zgc:111944	1
zgc:114139	1
zgc:153046	1
zgc:153342	1
zgc:153534	1
zgc:161973	1
zgc:171570	1
zgc:172285	1
zgc:174263	1
zgc:56556	1
zgc:56628	1
zgc:63734	1
zgc:63882	1
zgc:63947	1
zgc:66160	1

zgc:76867	1
zgc:76925	1
zp3	1
acat2	1
adat2	1
amd1	1
banp	1
ccdc25	1
ccr6a	1
cd164	1
cd2apl	1
ches1	1
chst14	1
churc1	1
dap1b_dup2	1
dcun1d4	1
dhcr24	1
ehd3	1
eif2b2	1
eif2b4	1
enah	1
ephx1	1
exoc1	1
fam20b	1
fam82a2	1
fam84a	1
galca	1
heca	1
hook1	1
igf2r	1
ints7	1
jag2	1
klf11a	1
klhl26	1
lats1	1
map3k7ip2	1
mia3	1
mut	1
myo6a	1
nat5	1
nbas	1
ndpkz6	1
pdcd6	1
pex3	1
pigc	1
pp2ca1	1

ppil4	1
ppp2r5c	1
pycrl	1
qdpra	1
rars2	1
rnf144ab	1
rngtt	1
rock2b	1
sars2	1
sccpdhb	1
scfd2	1
serac1	1
sesn1	1
si:ch211-142e24.2	1
si:ch211-147d7.2	1
si:ch211-199m3.9	1
si:ch211-203k16.1	1
si:ch211-208c9.4	1
si:ch211-20b12.1	1
si:ch211-22i13.2	1
si:ch211-244p18.3	1
si:ch211-260g14.3	1
si:ch211-278n15.1	1
si:ch211-59d15.5	1
si:ch211-59d15.9	1
si:ch211-63o20.5	1
si:ch211-89p3.3	1
si:dkey-177p2.3	1
si:dkey-177p2.6	1
si:dkey-183n20.16	1
si:dkey-217m5.1	1
si:dkey-221h15.1	1
si:dkey-264g21.1	1
si:dkey-34f16.5	1
si:dkey-77p13.2	1
si:dkey-85n7.5	1
si:dkey-86e18.1	1
si:dkeyp-55f12.3	1
si:rp71-81o21.1	1
sirt5	1
sirt6	1
slc30a1	1
slc35b2	1
snapc1a	1
snx14	1
srp9	1

stmn4	1
taf1b	1
tube1	1
vash2	1
vps18	1
zgc:101706	1
zgc:103418	1
zgc:103638	1
zgc:110175	1
zgc:110211	1
zgc:114082	1
zgc:136239	1
zgc:152769	1
zgc:153725	1
zgc:162161	1
zgc:171813	1
zgc:175221	1
zgc:55327	1
zgc:56518	1
zgc:56581_dup1	1
zgc:56581_dup2	1
zgc:63554	1
zgc:63562	1
zgc:63695	1
zgc:77563	1
zgc:86635	1
zgc:92063	1
zgc:92470	1
zgc:92716	1
zgc:92758	1
zp2.2	1
aldoc	1
arpc3	1
c1qtnf3	1
clk4b	1
crcp	1
dlat	1
ftsj1	1
gpn3	1
hbegf	1
hpse	1
ik	1
im:7160157	1
jak2a_dup2	1
jam3	1
klhl8	1

lmbrd2b	1
LOC100002393	1
med15	1
ndfip1l	1
ndr1	1
neu3.2	1
nf2	1
noc4l	1
nop14_dup1	1
otul	1
p2rx4a	1
pacs1	1
pcf11	1
pvrl3l	1
rab24	1
rars	1
rnf14	1
rnmtl1-b	1
sart1	1
sept4a	1
sfrs1	1
sfrs8	1
slc25a14	1
slc7a3	1
snx12	1
stom	1
stx5al	1
tcf3	1
tpst1l	1
traf4b	1
wdr55	1
zgc:101028	1
zgc:101635_dup1	1
zgc:101635_dup2	1
zgc:101639	1
zgc:103720	1
zgc:110031	1
zgc:110063	1
zgc:110224	1
zgc:110308	1
zgc:110540	1
zgc:113019	1
zgc:113162	1
zgc:123260	1
zgc:136279	1
zgc:158449	1

zgc:158624	1
zgc:158847	1
zgc:163077	1
zgc:171284	1
zgc:172359	1
zgc:56372	1
zgc:63572	1
zgc:77214	1
add3a	1
btbd2	1
camsap1l1	1
cherp	1
ddx59	1
dnaja3b	1
ell	1
fbxo42	1
insrb	1
kdm4b	1
klf2a	1
LOC562920	1
LOC563808	1
LOC792919	1
LOC798111	1
mbd3b	1
med20	1
mef2b	1
midn	1
nek7	1
pla2g6	1
pxk	1
rassf1	1
safb	1
si:ch211-129c21.6	1
si:ch211-197g15.3	1
si:ch211-226h8.14	1
si:ch211-239j15.4	1
si:ch211-258l4.3	1
si:ch211-258l4.7	1
si:dkey-171o17.2	1
si:dkey-19f23.2	1
si:dkey-236e20.6	1
si:dkey-254a11.3	1
si:dkey-286j15.3	1
si:dkey-44g23.7	1
si:dkeyp-53d3.5	1
si:dkeyp-84a8.8	1

slc26a11	1
slc30a7	1
slc6a6	1
tspan7b	1
ttc39c	1
ubxn6	1
uchl5	1
vps4a	1
zgc:110252	1
zgc:113191	1
zgc:113259	1
zgc:136330	1
zgc:136939	1
zgc:153290	1
zgc:153312	1
zgc:153635	1
zgc:153713	1
zgc:154106	1
zgc:158268	1
zgc:171490	1
zgc:171699	1
zgc:174165	1
zgc:175165	1
zgc:56068	1
zgc:56533	1
zgc:85972	1
zgc:92654	1
zgc:92701	1
atp6ap1	1
c1galt1a	1
c20orf14_dup2	1
casp9	1
ccbl2	1
csad	1
csrp1	1
dffa	1
dnmt4	1
dpm1	1
elovl4	1
eno1	1
fbxo25	1
fbxo44	1
fitm2	1
foxp1a	1
gpr157	1
hbs1l	1

icmt	1
kif1b	1
LOC553453	1
lrrc4c	1
mfsd5	1
ndnl2	1
otud3	1
pcsk5b	1
pfkfb2	1
pgd	1
pik3cd	1
prim1	1
prkcbp1l	1
sec14l1	1
slc9a8	1
sorbs3	1
spata2	1
src	1
taf8	1
tcea2	1
ttpal	1
wu:fi49f09	1
zgc:101056	1
zgc:101574	1
zgc:101621	1
zgc:110243	1
zgc:110684	1
zgc:110708	1
zgc:110775	1
zgc:112248	1
zgc:112481	1
zgc:113278	1
zgc:123113	1
zgc:136762	1
zgc:136901	1
zgc:153354	1
zgc:153623	1
zgc:153916	1
zgc:158609	1
zgc:158828	1
zgc:165430	1
zgc:194450	1
zgc:55746	1
zgc:56526	1
zgc:63672	1
zgc:92018	1

zgc:92784	1
zhx3	1
abcd3a	1
acot9	1
aggf1	1
anxa13	1
cdk8	1
cdv3	1
commd3	1
copeb	1
cpne3	1
ctdspla	1
decr2	1
fam164a	1
galnt11	1
gnal	1
hs6st1b	1
hspc049l	1
idi1	1
lztfl1	1
mak	1
mllt10	1
ndufb4	1
oxsr1b	1
rala	1
rdh10a	1
rdh12l	1
rhot2	1
rps6ka3b	1
serpinb1l3	1
slc35b3_dup1	1
slc35b3_dup2	1
stau2	1
stub1	1
tfap2a	1
top1mt_dup2	1
usp12	1
wdr24	1
wdr41	1
zgc:101569	1
zgc:110066	1
zgc:112043	1
zgc:112982	1
zgc:113037	1
zgc:123165	1
zgc:123336	1

zgc:152948	1
zgc:153492	1
zgc:158623_dup1	1
zgc:162250	1
zgc:162318	1
zgc:162928	1
zgc:171474	1
zgc:171977	1
zgc:172324	1
zgc:56530	1
zgc:73144	1
zgc:92030	1
zgc:92591	1
actr6	1
adpgk	1
alkbh3	1
btbd10a	1
ca5	1
ciapin1	1
dnajc24	1
edc3	1
ergic2	1
far1	1
frs2	1
galk2	1
ldha	1
ldhd	1
LOC564413	1
mpped2	1
mrpl23	1
parva	1
ppfibp2b	1
pth1a	1
rad52	1
saal1	1
scamp2	1
slc25a44a	1
tcp11l1	1
tsga14	1
unc45a_dup1	1
unc45a_dup2	1
vac14	1
wu:fc89g05	1
wu:fd21f07	1
wu:fi41d10	1
wwox_dup1	1

zgc:101566	1
zgc:101783	1
zgc:103499	1
zgc:112015	1
zgc:112084	1
zgc:113426	1
zgc:114150	1
zgc:136689	1
zgc:136858	1
zgc:153343	1
zgc:153556	1
zgc:153935	1
zgc:154036	1
zgc:154077	1
zgc:158861	1
zgc:162280	1
zgc:162420	1
zgc:162565	1
zgc:162634	1
zgc:171459	1
zgc:194879	1
zgc:55652	1
zgc:64166	1
zgc:66110	1
zgc:66449	1
zgc:77158	1
zgc:92512	1
zgc:92795	1
zgc:92915	1
anxa4	1
arhgdig	1
atp13a	1
atxn7l3	1
baxb	1
brd4	1
carhsp1	1
ccnf	1
cd2bp2	1
clcn7	1
copz2	1
cyp3c1	1
cyth1	1
dnajb1	1
dnm2	1
flr	1
fus	1

gcdhl	1
gipc1	1
gtf2f1	1
gys1	1
hirip3	1
jmjd5	1
kri1l	1
map2k4	1
mif4gdb	1
mlx	1
mvp	1
ndufa6	1
nmt1a	1
nsmce1	1
nubp1	1
nudt1	1
pcnt1	1
pdap1	1
pgls	1
pigq	1
pih1d1	1
polr3k	1
psmg3	1
rbbp6l	1
rcn3	1
retsat	1
rnf113a	1
rsad1	1
sh2b1	1
si:ch211-175l6.9	1
si:dkey-121j17.1	1
si:dkey-204f11.62	1
smg1	1
smurf2	1
strada	1
thraa	1
tmem184a	1
tmub2	1
ttyh2l	1
tubgl	1
vat1	1
wu:fb12c06	1
wu:fj33d10	1
xab2	1
xpnpep3	1
zgc:100999	1

zgc:103564	1
zgc:109932	1
zgc:112000	1
zgc:112270	1
zgc:112350	1
zgc:112352	1
zgc:112404	1
zgc:113210	1
zgc:113380	1
zgc:113411	1
zgc:123046	1
zgc:136827	1
zgc:153240	1
zgc:153257	1
zgc:153465	1
zgc:153624	1
zgc:153652	1
zgc:154097	1
zgc:158403	1
zgc:158419	1
zgc:158799	1
zgc:158860	1
zgc:162198	1
zgc:162544	1
zgc:162613	1
zgc:163109	1
zgc:165666	1
zgc:175094	1
zgc:195139_dup1	1
zgc:56230	1
zgc:56719	1
zgc:73356	1
zgc:77244	1
zgc:77462	1
zgc:86935	1
zgc:92244	1
znf598	1
agbl5	1
apaf1	1
arid2	1
ccdc53	1
cerk	1
chchd3	1
cmas	1
cwf19l1	1
cyb5r3	1

dera	1
dmd	1
dopey2	1
dyrk2	1
fbxo7	1
fgd6	1
ifrd1	1
im:6894100	1
kin	1
lmf2	1
LOC100148543	1
LOC791572	1
mrps33	1
nup37	1
orc5l	1
parp12b	1
pawrl	1
pmpcb	1
prkcq	1
pwp1	1
rap1b	1
sbf1	1
si:ch211-117l16.1	1
si:ch211-125a15.1	1
si:ch211-152c2.4	1
si:ch211-203h15.1	1
si:ch211-239e6.4	1
si:ch211-51e12.6	1
si:ch211-92l17.2	1
si:dkey-183c2.4	1
si:dkey-217k21.3	1
si:dkey-21h14.2	1
si:dkey-31f5.7	1
smo	1
taf3	1
tbc1d22a	1
terfa	1
tmcc3	1
trmu	1
uhrf1bp1l	1
wu:fb95g02	1
zgc:110307	1
zgc:110447	1
zgc:112202	1
zgc:114172	1
zgc:123180	1

zgc:123333	1
zgc:158603	1
zgc:162948	1
zgc:162956	1
zgc:194209_dup1	1
zgc:194209_dup2	1
zgc:55308	1
zgc:56178	1
zgc:63566	1
zgc:63570	1
zgc:92033	1
zgc:92448	1
zgc:92620	1
aatf	1
aldh2b	1
ankfy1	1
ankra2	1
ascc2	1
atp6v1al	1
bri3bp	1
ccbl1	1
ciz1	1
col4a3bp	1
crfb6	1
dpagt1	1
ephb4a	1
erlin2	1
fcho2	1
gbgt1l4	1
gfm2	1
Gpsm1	1
gsnb	1
gtf2h2	1
gtf3aa	1
h2afv	1
h3f3c	1
hint2	1
hmbsa	1
kcmf1	1
kptn	1
lmbrd2a	1
lmo4	1
LOC100150029	1
LOC556864	1
LOC559192	1
LOC561123	1

LOC561959	1
LOC564669	1
LOC565190	1
LOC565400	1
LOC569543	1
LOC569656	1
LOC793882	1
LOC796385	1
mapkapk5	1
marveld2b	1
mbd2_dup1	1
med22	1
mks1	1
mtmr12	1
mtmr2	1
mtmr8	1
mybbp1a_dup1	1
mybbp1a_dup2	1
ncor1	1
ndor1	1
ndufs4	1
nova1	1
oor	1
pex12	1
piwil2	1
pole	1
pomt1	1
ptcd2	1
rilpl2	1
rnf181	1
sfrp1a	1
si:ch211-66i11.1	1
si:dkey-110k5.6	1
si:dkey-127j5.5	1
si:dkey-170o10.3	1
si:dkey-201c13.3	1
si:dkey-3h3.3	1
si:dkeyp-110c7.1	1
slc25a25b	1
slc25a46	1
smarca2	1
taf1	1
taf6	1
tmem161b	1
tmem175	1
tmem2	1

tnfsf10l4	1
trmt2a	1
trpv4	1
uap1l1	1
usp30	1
yipf6	1
ypel1	1
zdhhc5	1
zgc:100861	1
zgc:100974	1
zgc:101609	1
zgc:103473	1
zgc:103561	1
zgc:110112	1
zgc:110219	1
zgc:110329	1
zgc:110331	1
zgc:112362	1
zgc:112985	1
zgc:114185	1
zgc:158784	1
zgc:163008_dup2	1
zgc:171426	1
zgc:56466	1
zgc:63910	1
zgc:65779	1
zgc:66242	1
zgc:66317	1
zgc:77165	1
zgc:77391	1
zgc:77421	1
zgc:85772	1
zgc:85789	1
zgc:86715	1
zgc:86898	1
zgc:92510	1
zgc:92599	1
acox1	1
ak3l1	1
alg6	1
cep63	1
chd1l	1
dctn2	1
dlg1	1
eefsec	1
efcab7	1

ercc3	1
fancd2	1
gadd45a	1
gcdh	1
gga3	1
gpx1b	1
hemk1	1
her6	1
jak1	1
lnpb	1
mtx2	1
negr1	1
nipa1	1
nit2	1
os9	1
pacsin1	1
pars2	1
pik3r3	1
pob	1
ppig	1
ppil1	1
rab22a	1
rbm5	1
rhoae	1
si:ch211-10e8.6	1
si:ch211-10e8.7	1
si:dkey-98p3.1	1
slc6a13	1
smx5	1
sox21a	1
stau1	1
tgm2b_dup1	1
tprg1	1
ttc26	1
tubgcp5	1
tusc4	1
uap1	1
ubn2	1
utxl1	1
vdrb	1
zc3h10	1
zgc:101893	1
zgc:103456	1
zgc:103549	1
zgc:110671	1
zgc:110788	1

zgc:112435	1
zgc:113104	1
zgc:113227	1
zgc:113409	1
zgc:114087	1
zgc:114103	1
zgc:123305	1
zgc:153206	1
zgc:153949	1
zgc:162191	1
zgc:162722	1
zgc:162953	1
zgc:165594	1
zgc:172009	1
zgc:193690	1
zgc:66135	1
zgc:66453	1
zgc:73238	1
zgc:73345	1
zgc:77222	1
zgc:77817	1
zgc:85939	1
zgc:92024	1
zgc:92194	1
zgc:92785	1
znf207b	1
aco1	1
aprt	1
arnt2	1
cars	1
dctn1a	1
dhcr7	1
dhodh	1
dhx38	1
dis3l	1
dok1b	1
elp4	1
ext2	1
fbxo3	1
galns	1
ggh	1
hdac8	1
igf1rb	1
il13ra1	1
katnb1	1
LOC407698	1

LOC796180	1
mcee	1
men1	1
mfge8l	1
mns1	1
mrpl15	1
nae1	1
ncapg2	1
neo1	1
nit1	1
nqo1	1
nr1h3	1
otub1	1
oxa1l	1
phf23b	1
pik3c3	1
ptpmt1	1
rbpjb	1
rnaseka	1
rras2	1
sc:d837	1
scrib	1
scube2_dup2	1
si:ch73-314g15.3	1
si:dkey-173l11.4	1
sirt3	1
sltm	1
spg21	1
tk2	1
tmed3	1
traf6	1
wu:fk11d03	1
ywhae2	1
zgc:101867	1
zgc:109889	1
zgc:110184	1
zgc:110591	1
zgc:113403	1
zgc:152898	1
zgc:152958	1
zgc:153233	1
zgc:153296	1
zgc:153347	1
zgc:153377	1
zgc:153587	1
zgc:153732	1

zgc:158285	1
zgc:158430	1
zgc:158452_dup1	1
zgc:158636	1
zgc:162269	1
zgc:162401	1
zgc:163000	1
zgc:163003	1
zgc:163091	1
zgc:171682	1
zgc:171731	1
zgc:175152	1
zgc:55634	1
zgc:55686	1
zgc:64136	1
zgc:77041_dup1	1
zgc:92594_dup2	1
zgc:92643	1
abl2	1
adam9	1
anapc5	1
ap3s1	1
apeh	1
arpc5l	1
bmp1a	1
bnip3l2	1
cpt2	1
cxxc1l	1
derl3	1
dffb	1
dnajc5aa	1
dock5	1
ercc8	1
fem1c	1
fgfr1a	1
gfpt1	1
gkap1	1
gnb1	1
hcfc1b	1
hirip5	1
lgtn	1
LOC100003663	1
LOC556245	1
LOC562755	1
LOC563100	1
LOC567180	1

LOC567359	1
LOC567547	1
mapk4	1
mmp9	1
ndufaf2	1
npl	1
otud5	1
pex10	1
pggt1b	1
plxna3	1
pold2	1
prkar2aa	1
qars	1
rbm39b	1
rexo1	1
rfx2	1
rgl1	1
rorca	1
sec22bb	1
sept5a	1
sh3gl1b	1
si:ch211-198n5.11	1
si:ch211-199o1.5	1
si:ch73-250d21.1	1
si:dkey-6n6.4	1
si:dkeyp-22b2.2	1
slc25a37	1
slc7a4	1
srp19	1
stap2b	1
suds3	1
suv39h1a	1
tcf7l1b	1
tmem127	1
tmem9	1
tpd52l2a	1
vps13a	1
xpc	1
zgc:100817	1
zgc:101122	1
zgc:101664	1
zgc:101841	1
zgc:101894	1
zgc:103495	1
zgc:103670	1
zgc:110020	1

zgc:110083	1
zgc:110697_dup1	1
zgc:110697_dup2	1
zgc:112263	1
zgc:112962	1
zgc:114043_dup1	1
zgc:114043_dup2	1
zgc:114095	1
zgc:136971	1
zgc:153958	1
zgc:153997	1
zgc:154013	1
zgc:154089	1
zgc:158415	1
zgc:162251_dup1	1
zgc:172062	1
zgc:195008	1
zgc:56538	1
zgc:63479	1
zgc:63587	1
zgc:63767	1
zgc:65891	1
zgc:66430	1
zgc:77486	1
zgc:86609	1
zgc:91897_dup1	1
zgc:91897_dup2	1
zgc:92850	1
appb	1
arhgef7a	1
bcs1l	1
brp44	1
ccdc93	1
clybl	1
dnajc10	1
dnajc28	1
dnajc3	1
ehhadh	1
fkbp7	1
fn1	1
fstl1b	1
fundc1	1
gpr161	1
gtdc1	1
hibch	1
igfbp2a	1

ing1	1
inpp4a	1
insig2	1
iqwd1	1
kdelc1	1
LOC100001896	1
LOC555224	1
LOC561913	1
LOC562077	1
LOC566620	1
LOC791769	1
mospd2	1
mycbp2	1
pdia5	1
pknox1.1	1
pms1	1
pofut2	1
prmt2	1
rabl3	1
ralb	1
rbm45	1
rnaseh2b	1
rp42-pen	1
rpe	1
10-Sep	1
sft2d3	1
si:ch211-125e6.12	1
si:ch211-194d6.3	1
si:ch211-194d6.5	1
si:ch211-219a4.3	1
si:ch211-219a4.7	1
si:dkey-101k6.7	1
si:dkey-204g5.2	1
si:xx-35d8.1	1
slc15a2	1
slc35a5	1
slc39a10	1
slc9a2	1
tmem41aa	1
traf3ip1	1
ube2e3	1
ubxn4	1
wars2	1
wdfy2	1
wdr3	1
zgc:100963	1

zgc:110065	1
zgc:110221_dup1	1
zgc:110221_dup2	1
zgc:113314	1
zgc:152951	1
zgc:153663	1
zgc:153679	1
zgc:158320	1
zgc:158619	1
zgc:162619	1
zgc:66484	1
zgc:77010	1
zgc:77397	1
zgc:77734	1
zgc:85702_dup1	1
zgc:85702_dup2	1
zgc:92393	1
zgc:92925	1
capn9	2
mtp	2
zgc:101052	2
zgc:136353	2
zgc:64040	2
ewsr1a	2
hist2h2l	2
tcf7l1a	2
zgc:136359	2
b3gnt5	2
bmp7a	2
gnl3	2
nfya	2
rbm12	2
sfrs6b	2
zgc:110712	2
zgc:153665	2
calm2b	2
cyp26a1	2
mxtx2	2
pea3	2
cst3	2
eif3s6ip	2
fgf8a	2
flh	2
ism1	2
ldb1a	2
vent	2

zgc:153911	2
fb06f03	2
gpc4	2
her5	2
hs6st2	2
zgc:153018	2
hif1al	2
sesn3	2
ccne2	2
esrp1	2
fam46b	2
ubin	2
zgc:136360	2
zgc:161979	2
gsc	2
lft2	2
ywhaqb	2
yy1a	2
zgc:153175	2
zgc:92069	2
foxa3	2
wee1	2
zgc:112012	2
fuca1	2
pip5k1a	2
si:ch211-191i18.4	2
txnip	2
cnn2	2
elovl1a	2
gadd45bl	2
gata6	2
hes6	2
klf4	2
ptbp1a	2
zgc:163040	2
znfl2a	2
bmp2b	2
fau	2
hsd3b1	2
lft1	2
mcm3	2
rhov	2
zgc:109978	2
cldnb	2
clica	2
fgfr4	2

otub1l	2
blf	2
ier5	2
lmnb2	2
pak2b	2
si:ch211-262h13.3	2
zgc:100942	2
zgc:110289	2
zgc:112994	2
dnmt3	2
hnf4a	2
irx7	2
slc2a12	2
zgc:165664	2
snai2	2
tgif1	2
zgc:136591	2
zgc:56683	2
dusp6	2
sept7b	2
zgc:114037	2
arl5c	2
eve1	2
igf2bp1	2
lfng	2
notum3	2
ppl	2
zgc:100900	2
zgc:110779	2
zgc:112397	2
btg1	2
svopl	2
yeats4	2
anxa1a	2
ctsll	2
drl	2
fbp1b	2
fut9	2
her7	2
ier5l	2
si:ch211-114c12.2	2
wnt11	2
arl6ip5	2
atf4	2
atp1b1a	2
erm	2

mcm6	2
pfkfb4l	2
rheb	2
sb:cb742	2
zgc:161969	2
cdkn1c	2
hmgb3a	2
map1lc3b	2
plekhf1	2
sox32	2
zgc:110425	2
zgc:163061	2
zgc:171804	2
zgc:173587	2
zgc:55456	2
acer1	2
acsbg2	2
aplnra	2
dnmt7	2
foxd5	2
mgll	2
pim1	2
rhoaa	2
rhoab	2
si:ch211-15b7.3	2
si:ch211-170d8.2	2
vgll4l	2
wu:fb63a09	2
cbsb	2
fzd7a	2
gabpa	2
has2	2
klf2b	2
lcp1	2
mki67ip	2
zgc:136896	2
plk1	3
prdx2	3
zgc:55877	3
zgc:56493	3
btg3	3
ppial	3
slc16a3	3
tacc3	3
eef1g	3
gstp1	3

hspa9	3
lamp2	3
zgc:114130	3
zgc:77118	3
kif23	3
zgc:162879	3
mylipa	3
nutf2	3
ssr2	3
acadm	3
mknk2a	3
pif1	3
plk3	3
zgc:55701	3
chac1	3
gtf2a1	3
ppp1r3b	3
zgc:103482	3
zgc:158856	3
btg2	3
birc5b	3
ppdpfb	3
spsb1	3
zgc:162349	3
ppib	3
dnmt1	3
kpna2	3
sinup	3
thy1	3
zgc:153251	3
nasp	3
mad2l1	3
lipg	3
slc25a25	3
zgc:195245	3
anp32b	4
zic2b	4
ved	4
id1	4
sfrs5a	4
zgc:193505	4
aldob	4
sox3	4
nnr	4
anp32e	4
sfpq	4

ptmab	4
krt5	4
zgc:153405	4
h2afx	4
zgc:153409	4
mych	4
ccnd1	4
zgc:77041_dup2	4
dynll2	4
ybx1	4
asb11	4
cx43.4	4
g12	4
ak3	5
oep	5
zgc:101846	5
ptbp1b	5
sult6b1	5
bambi	5
marcksl1	5
vox	5
rbmx	5
zgc:77366	5
cldne	5
rps5	5
zgc:110251	5
cxcr4b	5
zgc:123210	5
cki	5
ntla	5
stm	5
zgc:101594	5
zgc:109868	5
zgc:112234	5
zgc:153349	5
zgc:55813	5
zgc:101000	5
zgc:92061	5
rps21	5
zgc:110216	5
zgc:113984_dup2	5
zgc:171937	5
zgc:173552	5
rpl27	5
rps15a	5
zgc:103629	5

zgc:153632	5
hnrpl	5
shisa2	5
si:dkey-252h13.6	5
sox19a	5
cxcr4a	5
sfrs3a	5
slc25a22	5
anp32a	5
zgc:154164	5
zgc:158350	5
zgc:56141	5
cebpb	5
slc16a1	5
tbx16	5
zgc:123194	5
zgc:195154	5
LOC796588	6
uchl1	6
zgc:56565	6
zgc:111868	6
cfl2l	6
tubb2c	6
si:ch211-132b12.8	6
zgc:56419	6
cdc20	6
rdh10b	6
si:ch211-14a17.7	6
ckbb	6
LOC100001110	6
pfn2	6
eno3	6
sec61g	6
hsp90b1	6
ldhb	6
hspa5	6
atp5g	6
zgc:77112	6
fam46c	6
atf7ip	7
kpnb3	7
rpl24	7
rpl9	7
elovl7b	7
oclna	7
setd8a	7

ucp2	7
zgc:101565	7
eif4a3	7
nucks1	7
rps15	7
sdc4	7
thoc7	7
zgc:109957	7
zgc:66127_dup2	7
zgc:77560	7
ppp4cb	7
zgc:171724	7
calm2a	7
cfl1	7
papolg	7
zgc:162603	7
ctnna_dup3	7
mmgt1	7
prp19	7
6-Sep	7
zgc:55886	7
hnrnpl2	7
kpna4	7
zgc:153795	7
zgc:158363	7
cct3	7
rps19	7
rps9	7
ywhabl	7
zgc:73262	7
zgc:158367	7
zgc:77304	7
caprin1b	7
ctcf	7
ctsd	7
rps3	7
ube2n	7
eif1b	7
eif3s2	7
psmb4	7
rpl14	7
rpl30	7
u2af2b	7
zgc:101724	7
fubp1	7
gng5	7

ivns1abpb	7
si:dkey-175g20.1	7
zgc:152779	7
zgc:55492	7
max	7
rps29	7
snrpd1	7
cnot6	7
cnot8	7
hmgb3b	7
nhp2	7
nop56	7
rpl28l	7
rrm1	7
skp1	7
cbx5	7
rpl10	7
rps26	7
tkt	7
zgc:109926	7
arf1l	7
api5	7
metap2	7
prc1	7
rsl24d1	7
zgc:77235	7
bud31	7
ilf3	7
rpl3	7
smarce1	7
zgc:85729	7
strap	7
btf3	7
cdh1	7
fam76b	7
setd8b	7
snrnp27	7
tia1	7
zgc:86607	7
pl10	7
rps10	7
snrpc	7
ssb	7
ywhab1	7
cct7	7
eif4g2a	7

gpt2	7
myl9l	7
nat11	7
puf60b	7
rbm4.2	7
rplp2l	7
rps4x	7
bcas2	7
ywhab2	7
pcbp2	7
rif1	7
bactin1	8
cldng	8
ccna1	8
zgc:110304	8
mid1ip1	8
ctssb.1	8
gapdh	8
ctsba	8
mt2	8
gmnn	8
rrm2	8
cldnd	8
btg4	8
acp5a	8
ccnb2	8
cth1	8
fth1	8
h2afvl	9
hspa8	9
khdrbs1a	9
hnrnpa0l	9
ef1a	9
cirbp	9
bactin2	9
h3f3a	9
ctsl1a	9
ankrd10a	10
arhgap10_dup1	10
arhgef7b_dup1	10
arhgef7b_dup2	10
cbr1	10
cep97	10
chordc1	10
dctd	10
dnaja1l	10

elf2		10
fam114a1		10
fbxl18		10
fbxo8		10
htt		10
LOC564372		10
lsm6		10
mmaa		10
mrpl39		10
ncapd3		10
	1-Oct	10
pane1		10
pdcd11		10
pgam1b		10
pibf1		10
pin1		10
rnaseh2a		10
si:dkey-170e5.1		10
spast		10
spcs3		10
taf5		10
tm9sf2		10
trappc5		10
ube2k		10
uso1		10
zgc:109809		10
zgc:112426		10
zgc:123253		10
zgc:136531		10
zgc:152968		10
zgc:153456		10
zgc:158370		10
zgc:162306		10
zgc:194985		10
zgc:56665		10
zgc:64148		10
zgc:66359		10
zgc:66472		10
zgc:73124		10
zgc:73375		10
zgc:76977		10
zgc:77739		10
zgc:77849		10
zgc:77880		10
aldh7a1		10
arfip2b		10

arrb2a	10
brap	10
cops4	10
ddx55	10
denr	10
enoph1	10
exosc8	10
hip1r	10
ilk	10
LOC559111	10
march5l	10
med11	10
nup155	10
ptrh2	10
rabgef1	10
rfc3	10
ripk4	10
sardh	10
slc12a2	10
slc27a6	10
smad2	10
snx30_dup1	10
tbl2	10
thop1	10
tpte	10
ubap1	10
uvrag	10
ywhah	10
zgc:101722	10
zgc:103508	10
zgc:110234	10
zgc:112025	10
zgc:112247	10
zgc:114173	10
zgc:114199	10
zgc:153463	10
zgc:162425	10
zgc:165539	10
zgc:173506	10
zgc:56317	10
zgc:56702	10
zgc:66327	10
zgc:77183	10
zgc:77306	10
zgc:85678	10
zgc:92611	10

znf259	10
afmid	10
appl1	10
cdkal1	10
cnpy2	10
cpne1	10
dido1	10
egln1	10
elovl1b	10
fam116a	10
gnai2l	10
hug	10
jup	10
ldb1b	10
LOC558435	10
LOC572324	10
maf1	10
mapk14b	10
mybl2	10
npepps	10
prickle2	10
rab43	10
rnpep	10
slc35e1	10
sypl2a	10
tmf1	10
yod1	10
zgc:100839	10
zgc:101614_dup1	10
zgc:101614_dup2	10
zgc:110000	10
zgc:110298	10
zgc:110815	10
zgc:136374	10
zgc:136866	10
zgc:153041	10
zgc:153376	10
zgc:153943	10
zgc:153972	10
zgc:154079	10
zgc:158357	10
zgc:158644_dup1	10
zgc:162816	10
zgc:162965	10
zgc:175088	10
zgc:175264	10

zgc:56077	10
zgc:56630	10
zgc:73111	10
zgc:77929	10
zgc:92502	10
zgc:92610	10
zgc:92628	10
zgpat	10
acsf2	10
adoa	10
aldh18a1	10
asb3	10
atad1b	10
bc2	10
birc5a	10
camk2g1	10
ccdc6b	10
cdkrap3	10
chmp6l	10
cript	10
dock1	10
eif2ak1	10
eif3c	10
ercc4	10
fam53b	10
glrx3_dup1	10
glrx3_dup2	10
gna13a	10
gpsm2l	10
klhl11	10
l3mbtl2	10
llgl2	10
map2k6_dup2	10
med24_dup2	10
mgrn1_dup1	10
mtr	10
nrbf2	10
nsfb	10
pank1b	10
phkg2	10
psmc5	10
rab18a	10
rfng	10
rhoq_dup1	10
slc9a3r2	10
tob1b	10

usp22	10
wdr45l	10
xrcc6	10
zgc:101662	10
zgc:110577	10
zgc:110659	10
zgc:112322	10
zgc:113056	10
zgc:158612	10
zgc:162286	10
zgc:174164	10
zgc:195155	10
zgc:55317	10
zgc:56706	10
zgc:64155	10
zgc:92655	10
adh8b	10
bscv	10
cdkn3	10
cog2	10
def	10
echs1	10
LOC566872	10
LOC570267	10
LOC794975	10
LOC798687	10
LOC799087	10
mapksp1	10
mark3	10
mfsd2a	10
mnat1	10
msh6	10
pcbd1	10
phf10	10
pp2cb	10
ppif	10
ppp2r5eb	10
psmb1	10
rab23	10
rtf1	10
sav1	10
sec23ip	10
sgpl1	10
si:dkey-162b3.1	10
si:dkey-76k16.7	10
slc25a28	10

slc38a6	10
slkn	10
smap1	10
tial1	10
trmt61a	10
tspan15	10
vti1b	10
wdr22	10
yipf3	10
zdhhc16	10
zgc:101107	10
zgc:103457	10
zgc:110319	10
zgc:113369	10
zgc:114078	10
zgc:114169	10
zgc:136403	10
zgc:136474	10
zgc:153407	10
zgc:153646	10
zgc:158849	10
zgc:174575	10
zgc:193664	10
zgc:92670	10
asah1a	10
aup1	10
canx	10
cops6	10
ddx41_dup1	10
ddx41_dup2	10
dnd	10
exoc7	10
foigr	10
hars	10
LOC556124	10
magt1	10
matr3l	10
mcts1	10
narg1a	10
nkapl	10
nsdhl	10
nudt22	10
nxt2	10
pdzd11	10
polr2b	10
prps1b	10

smyd5	10
stx3a	10
taf7	10
tmem33	10
wnt8-2	10
xkrx	10
zgc:112345	10
zgc:152864	10
zgc:153259	10
zgc:153400	10
zgc:153537	10
zgc:175091	10
zgc:63637	10
zgc:73136	10
zgc:92770	10
agfg1a	10
arfip2a	10
bccip	10
clptm1	10
crsp6	10
dhx16	10
ercc2	10
fancj	10
farsl	10
gemin4	10
lsrl	10
mett10d	10
mpzl3	10
mrpl38	10
mrps23	10
msi2b	10
nmd3	10
paf1l	10
panx1	10
rad1	10
sh3bp5la	10
si:dkey-24p1.5	10
supt5h	10
timm50	10
trappc4	10
trappc6bl	10
txndc17	10
usp16	10
vps26b	10
vps53	10
wdfy1_dup1	10

zgc:101710	10
zgc:103530	10
zgc:110266	10
zgc:110702	10
zgc:110726	10
zgc:110801	10
zgc:113149	10
zgc:152873	10
zgc:153051	10
zgc:153084	10
zgc:153258	10
zgc:154041	10
zgc:162306_dup2	10
zgc:162962	10
zgc:165596	10
zgc:77828	10
zgc:91936	10
adar	10
atg5	10
atp6v1c1	10
bat5l	10
bckdhb_dup1	10
bckdhb_dup2	10
ccdc12	10
cnot10	10
coq3	10
dap3	10
dctn3	10
dedd1	10
dhdds	10
dnase2	10
dscc1	10
dtnbp1a	10
epn1	10
ext1a	10
fkbp14	10
flot1b	10
gnl1	10
hibadhb	10
LOC561889	10
mboat7	10
mrpl13	10
ndrg1l	10
otud7b	10
pafah1b3	10
pbx2	10

pmpca	10
prpf31	10
rab5al	10
rbm42	10
rpp38	10
sh3bp5	10
si:dkey-77n11.3	10
snx27	10
sri	10
taf2	10
ubr5	10
zgc:100811	10
zgc:100980	10
zgc:110255	10
zgc:113841	10
zgc:136551	10
zgc:153301	10
zgc:153892	10
zgc:153909	10
zgc:158611	10
zgc:158863	10
zgc:171672	10
zgc:175096	10
zgc:194152	10
zgc:194983	10
zgc:56148	10
zgc:56175	10
zgc:63565	10
zgc:64102	10
zgc:66448	10
zgc:76869	10
zgc:86749	10
zgc:86850	10
zgc:92356	10
zgc:92801	10
zhx2	10
acss1	10
adi1	10
atp6v1ba	10
brms1l	10
clic4	10
crip2	10
ctbp2	10
degs2	10
dicer1	10
eapp	10

ecd	10
fam54b	10
fbxo30	10
foxo3a	10
id3	10
ikzf5	10
mrpl35	10
ndufb8	10
nol10	10
plk4	10
pomt2	10
ppie	10
psen1	10
ptena	10
rab3gap2	10
rmnd1	10
rps6ka1	10
sec23a	10
si:ch211-154e15.3	10
si:ch211-15d5.5	10
slc8a1b	10
snx9	10
sptlc2_dup2	10
srrm1	10
strn3	10
tbpl2	10
traf3	10
vps24	10
wu:fa98a01	10
xrcc4	10
yy1b	10
zgc:100799	10
zgc:110317	10
zgc:110621	10
zgc:136874	10
zgc:153540	10
zgc:153680	10
zgc:153748	10
zgc:56576	10
zgc:63942	10
zgc:63962	10
zgc:66323	10
zgc:73290	10
zgc:85800	10
zgc:91861	10
zmpste24	10

zpcx	10
zranb1	10
aplp2	10
arih1	10
arl2bp	10
bcl2l13	10
bcl7b	10
ddb2	10
dye	10
edc4	10
fam65a	10
fkbp4	10
nat10	10
nip7	10
opa3	10
pcyt1ab	10
pdp2	10
pepd	10
rap2b	10
selt1b	10
si:ch211-107o23.1	10
si:ch211-160d20.3	10
si:ch211-203b8.5	10
si:ch211-218c6.5	10
si:ch211-238n5.5	10
si:dkey-12e7.1	10
si:dkey-12o15.1	10
si:dkey-223n17.3	10
si:dkey-6e12.6	10
si:dkey-8l13.1	10
si:dkey-8l13.4	10
slc30a4	10
snapc5	10
tcf25	10
tmod2	10
tp53i11	10
trpm7	10
zgc:101555	10
zgc:103562	10
zgc:110460	10
zgc:112180	10
zgc:153239_dup2	10
zgc:153393	10
zgc:153446	10
zgc:158614	10
zgc:162301	10

zgc:165591	10
zgc:175098	10
zgc:55794	10
zgc:65774	10
zgc:86917	10
zgc:92172	10
zgc:92663	10
adrbk2	10
ap2b1	10
apoa1bp	10
bet1	10
bop1	10
c1orf109	10
clasp2	10
cndp2	10
cnot3b	10
crat	10
dkfzp564o0523	10
dync1li1	10
ehmt2	10
eif3eb	10
ext1b	10
fam63a	10
fbxl2	10
gabpb2	10
gstr1	10
laptm4b	10
LOC100000325	10
LOC100190889	10
LOC566469	10
LOC568159	10
lyricl	10
mrpl9	10
ndufs6	10
nfe2l3_dup1	10
nudcd1	10
osbpl3a	10
pard6gb	10
pdcd6ip	10
ppp1r11	10
prcc	10
psmg2	10
rab5a	10
rbm24	10
rmi1	10
sdc2_dup2	10

sept7a_dup2	10
sf3a3	10
shfm1	10
si:ch211-173p18.1	10
si:ch211-194e15.1	10
si:ch211-203l9.2	10
si:ch211-204a13.4	10
si:ch211-207c15.3	10
si:ch211-254e15.1	10
si:dkey-109n11.2	10
si:dkey-184p18.2	10
si:dkey-204a24.2	10
si:dkey-211g8.3	10
si:dkey-263h23.4	10
si:dkeyp-114f9.3	10
si:dkeyp-24a7.1	10
si:dkeyp-34f6.1	10
si:dkeyp-39e9.2	10
stard3	10
trip13	10
zgc:100787	10
zgc:103514	10
zgc:110214	10
zgc:110717	10
zgc:113071	10
zgc:113125	10
zgc:158604	10
zgc:165573	10
zgc:55580	10
zgc:56010	10
zgc:85936_dup1	10
zgc:85936_dup2	10
zgc:86833	10
zgc:92196	10
acin1a	10
aldh9a1b	10
arhgef18	10
atp6v0b	10
atp6v1h	10
c3orf58a	10
cacybp	10
ccdc49	10
cul1a	10
eif2b5	10
extl2	10
gipc2	10

gk5	10
gorasp1	10
gpsm2	10
hiat1a	10
insra	10
klhl18	10
lamc1	10
lrrc42	10
mpv17l2	10
mrpl37	10
oxsr1a	10
pard3	10
pcyt1aa	10
pex19	10
pitrm1	10
plcd1b	10
rab3a	10
rabggtb	10
riok1	10
ripk2	10
rpp40l	10
scarb2	10
si:ch211-119o8.6	10
si:ch211-14a17.11	10
si:ch211-14a17.6	10
si:ch73-38p6.1	10
si:ch73-52e5.1	10
si:dkey-218h11.4	10
slc39a6	10
socs6b	10
sord	10
sppl2	10
ssbp3	10
tnika	10
tyw3	10
usp33	10
zfpl1	10
zgc:100856	10
zgc:101733	10
zgc:103579	10
zgc:110269	10
zgc:110680	10
zgc:113691	10
zgc:123256	10
zgc:123304	10
zgc:152896	10

zgc:153115	10
zgc:153254	10
zgc:153664	10
zgc:153847	10
zgc:158364	10
zgc:158807	10
zgc:195172	10
zgc:73198	10
zgc:73230	10
zgc:85851	10
zgc:92635	10
actr10	10
aida	10
arpc5b	10
bpnt1	10
btbd6	10
btbd7	10
ccm2	10
cdc42bpb	10
ddx49	10
dtng	10
eef1db	10
elp3	10
exoc2	10
ezr	10
fntb	10
kcnk5	10
mtmr9	10
parp1	10
pbk	10
ppat	10
prpf39	10
psmc1b	10
ralgps2	10
rbm25	10
rcl1	10
sec63	10
serinc1	10
si:busm1-189a20.4	10
si:ch211-11m18.3	10
si:ch211-147d7.3	10
si:ch211-223m11.2	10
si:ch211-245h14.3	10
si:ch211-59d15.8	10
si:ch211-63o20.7	10
si:dkey-11e23.5	10

si:dkey-15j16.2	10
si:dkey-174m14.2	10
si:dkey-177p2.14	10
si:dkey-241l7.6	10
si:dkey-260j18.2	10
si:dkey-30h14.2	10
si:dkey-97o5.1	10
si:dkeyp-50f7.2	10
si:dkeyp-50f7.3	10
si:dkeyp-55f12.4	10
si:zfos-47c12.1	10
sipa1l1	10
slain2	10
snx17	10
snx9l	10
taf1a	10
tmem165	10
tpr	10
vamp4	10
wdr21	10
xrcc3	10
zbtb2b	10
zgc:101562	10
zgc:101872	10
zgc:112330	10
zgc:136552	10
zgc:154018	10
zgc:162268	10
zgc:171230	10
zgc:194176	10
zgc:194598	10
zgc:55448	10
zgc:56136	10
zgc:86838	10
zp3a.1	10
zp3a.2	10
add1	10
asl	10
bod1	10
cdc23	10
chchd10	10
chek1	10
chm	10
ddt	10
ddx46	10
dnajc21	10

dpp3	10
ell2	10
eri1	10
fpgs	10
hmgcrb	10
mrps18c	10
mtmr1b	10
notch1a	10
phpt1	10
ptpra	10
rb1_dup1	10
rb1_dup2	10
sap30l	10
sara2	10
siah2l	10
sms	10
spna2	10
sra1	10
supt6h	10
unc119.2	10
vdac1	10
zcchc10	10
zgc:100829	10
zgc:101585	10
zgc:110527	10
zgc:110682	10
zgc:112071	10
zgc:112340	10
zgc:123047	10
zgc:123105	10
zgc:152927	10
zgc:152953	10
zgc:153153	10
zgc:154031	10
zgc:158748	10
zgc:158760	10
zgc:162319	10
zgc:174935	10
zgc:175288	10
zgc:193790	10
zgc:194693	10
zgc:55582	10
zgc:73264	10
zgc:77424	10
zgc:77551	10
zgc:77838	10

zgc:91985	10
zgc:92417	10
znf131	10
atp5d	10
atp6ap2	10
bcor	10
cry4	10
csrp2bp	10
dag1	10
dbt	10
gna11	10
gtpbp1l	10
hmg20b	10
hnrnpk	10
ippk	10
jagn1b	10
kif14	10
lass5	10
ncl1	10
pdhb	10
psmd1	10
rnf13	10
rpp21	10
rqcd1	10
sdhb	10
si:dkey-121a9.3	10
si:dkey-42i9.5	10
si:dkey-42i9.6	10
si:dkey-42i9.9	10
si:dkey-44g23.5	10
si:rp71-1n18.2	10
slc25a20	10
timm44	10
tjp3	10
tnfaip8	10
tsc22d2	10
xpnpep1	10
zgc:103434	10
zgc:103487_dup1	10
zgc:103487_dup2	10
zgc:110528	10
zgc:153700	10
zgc:55604	10
zgc:63694	10
zgc:66326	10
zgc:92026	10

zgc:92335	10
acss2	10
adrm1b	10
bcl2l	10
clstn1	10
ctnnbl1	10
dazap2	10
ddx23	10
dnajc11	10
eif4ba	10
fam50a	10
ftsjd2	10
itga5	10
lzic	10
ndrg3b	10
nr4a1	10
pdlim2	10
pfkfb1	10
plcg1	10
prosc	10
psmb6	10
rnf114	10
sas	10
sdf4	10
slc35c2	10
smc1al	10
syap1	10
trappc6b	10
txnl1	10
ube4b	10
zgc:101011	10
zgc:103688	10
zgc:113121	10
zgc:153696	10
zgc:171525	10
zgc:171696	10
zgc:172210	10
zgc:195326	10
zgc:55389	10
zgc:63466	10
zgc:77115	10
zgc:92251	10
zgc:92811	10
ankha	10
c3orf58b	10
ccdc52	10

cdk5	10
cnn3a	10
cops5	10
cul1b	10
cyb5a	10
fech	10
gnptg	10
klhl15	10
LOC560360	10
luc7l	10
mppe1	10
mtfr1	10
mtmr6	10
myd88	10
ncoa2	10
ngdn	10
nupl1	10
pabpn1	10
pbx4	10
pdss1	10
sgk3	10
sulf1	10
top1mt_dup1	10
wu:fb76c07	10
ythdf3	10
zgc:101668	10
zgc:101800_dup1	10
zgc:101800_dup2	10
zgc:113074	10
zgc:153329	10
zgc:153918	10
zgc:153980	10
zgc:162943	10
zgc:171750	10
zgc:174160	10
zgc:66014	10
zgc:66442	10
zgc:76981	10
zgc:77808	10
zgc:92086	10
zgc:92337	10
zgc:92392	10
zgc:92458	10
zmynd11	10
amfr_dup1	10
amfr_dup2	10

cdkn1b	10
chmp1a	10
cnot1	10
csk	10
dldh	10
dnm1l	10
etfa	10
gabarapl2	10
immp2l	10
LOC100001081	10
LOC567494	10
polr2c	10
prmt7	10
samm50	10
sh3gl3	10
si:ch211-51l3.4	10
tead1	10
tes	10
wdr51b	10
zgc:100959	10
zgc:100960	10
zgc:110551	10
zgc:153293	10
zgc:162128	10
zgc:162335	10
zgc:63491	10
zgc:66306_dup1	10
zgc:66306_dup2	10
zgc:91811	10
zgc:91862	10
zgc:92126	10
zgc:92689	10
zgc:92726	10
abat	10
acly	10
aldh16a1	10
ankrd54	10
atp6v0a1	10
atxn2l	10
axin2	10
baiap2l1b	10
baxa	10
cant1a	10
ccdc101	10
ccdc47	10
cdipt	10

crlf3	10
dctn5	10
ddx42	10
e4f1	10
farsa	10
foxk1	10
hgs	10
kdelr3	10
lasp1	10
LOC553327	10
med25	10
nar	10
ndufb10	10
nlk1	10
nosip	10
nudt9	10
prkar1a	10
prkcb1	10
psmc3ip	10
rab40c	10
rab5c	10
rac2	10
sepx1	10
sgsm3	10
snrnp70	10
stat3	10
tbc1d16	10
thoc6	10
tmed1	10
ttc1	10
ubtf	10
wu:fc10d10	10
zgc:101777	10
zgc:109927	10
zgc:110084	10
zgc:110325	10
zgc:110443	10
zgc:110800	10
zgc:153165	10
zgc:153192	10
zgc:153292	10
zgc:153477	10
zgc:158267	10
zgc:158422	10
zgc:163143	10
zgc:165518	10

zgc:171980	10
zgc:55259	10
zgc:56066	10
zgc:63976	10
zgc:66260	10
zgc:66474	10
zgc:73225	10
zgc:77127	10
zgc:86892	10
zgc:91845	10
zgc:92453	10
znf207a	10
atp6v1e1	10
atp6v1f	10
calua	10
cand1	10
creld2	10
dennd5b	10
dnajc2	10
fgfr1op2	10
foxm1l	10
gcc1	10
irf5	10
lta4h	10
med21	10
mkln1	10
mkrn1	10
ncaph2	10
nudt5	10
pacsin2	10
pgm3	10
plxnb2a	10
ppp1r12a	10
rbm17	10
si:ch211-116i17.1	10
si:ch211-125a15.2	10
si:ch211-240j22.5	10
si:ch211-286m4.4	10
si:ch211-51e12.4	10
si:ch211-95o16.2	10
si:dkey-180p18.9	10
si:dkey-202b22.4	10
si:dkey-207j16.2	10
slc26a5	10
tmbim4	10
tmem168a	10

twf1b	10
ube2h	10
waslb	10
zbed4	10
zcrb1	10
zgc:101062	10
zgc:103517	10
zgc:113263	10
zgc:162885	10
zgc:165515	10
zgc:73078	10
aacs	10
ankrd13a	10
ap1b1	10
bmp2k	10
bmpr1b_dup1	10
bmpr1b_dup2	10
brd3b	10
cdc26	10
cdc42ep2	10
cds2	10
cobra1	10
ddx54	10
dpf2	10
dpm2	10
ehmt1a	10
fam125ba_dup1	10
fam125ba_dup2	10
fam172a	10
fbxw5	10
ficd	10
gabarap	10
gbgt1l1	10
hdr	10
il13ra2	10
jak2b	10
kctd10	10
limk2	10
LOC556237	10
LOC558044	10
LOC561094	10
LOC561164	10
LOC561321	10
LOC563823	10
LOC565118	10
LOC570879	10

mapk1	10
mink1	10
mobkl1a	10
mrps31	10
myst3	10
nccrp1	10
nol6	10
nup88	10
pdcd8	10
pdha1a	10
pes	10
ppp2r4	10
psat1	10
ptrh1	10
pxn	10
rab14	10
rabep1	10
rad17	10
rchy1	10
rfc5_dup1	10
riok2	10
rsrc2	10
sh3glb2	10
si:ch211-207c6.1	10
si:dkey-1o2.1	10
si:dkey-98f17.2	10
si:dkeyp-15f12.2	10
slc2a8l	10
slc31a1	10
ttc33	10
ufc1	10
usp20	10
wdr36	10
zc4h2	10
zgc:100952	10
zgc:112524	10
zgc:112973	10
zgc:152955	10
zgc:153929	10
zgc:153990	10
zgc:158343	10
zgc:162938	10
zgc:163008_dup1	10
zgc:194800	10
zgc:56134	10
zgc:56688	10

zgc:64116	10
zgc:86625	10
zgc:92317	10
zswim7	10
akr1a1b	10
anapc11	10
arl8ba	10
atf7b	10
atg4b	10
bin1	10
bin2b	10
casp8	10
cish	10
ergic3	10
gga1	10
gorasp2	10
ica	10
ip6k2	10
lmo4l	10
LOC560131	10
mars	10
mtpn	10
mypt2	10
nipa2	10
ola1	10
plxna1	10
rae1	10
slc25a12	10
slc35d1a	10
slc9a7	10
st3gal3	10
stx8	10
suz12b	10
tbc1d23	10
th1l	10
tmbim1	10
tomm34	10
unc50	10
usp1	10
vapb	10
vhl	10
vstm2l	10
wdr82	10
zgc:101577	10
zgc:101803	10
zgc:112212	10

zgc:112231	10
zgc:136560	10
zgc:153172	10
zgc:158241	10
zgc:158381	10
zgc:158429	10
zgc:171476	10
zgc:56112	10
zgc:63986	10
zgc:86841	10
zranb2	10
acadvl	10
adam10a	10
b3gat3	10
chmp5	10
ctr9	10
dhx15	10
dnajb6	10
ecsit	10
esrp2	10
faah2b	10
furina	10
glceb	10
harbi1	10
hrasl	10
hsd17b12b	10
htatip2	10
kif7	10
mettl3	10
mfap1	10
mllt3	10
ndufs2	10
ndufs3	10
plaa	10
plcb3	10
pop7	10
prmt3	10
rtn3	10
senp3b	10
sf1	10
si:busm1-112p11.1	10
si:dkey-265m8.2	10
slc39a1	10
slc7a6os	10
snrpfl	10
syt13	10

tada2b	10
ttc5	10
zdhhc13	10
zgc:101113	10
zgc:101653	10
zgc:101657	10
zgc:101658	10
zgc:101765	10
zgc:103545	10
zgc:110763	10
zgc:112142	10
zgc:136860	10
zgc:136970	10
zgc:152651	10
zgc:153080	10
zgc:153369	10
zgc:153386	10
zgc:153610	10
zgc:158257	10
zgc:158452_dup2	10
zgc:162892	10
zgc:171967	10
zgc:193816	10
zgc:55262	10
zgc:55443	10
zgc:55664	10
zgc:56049	10
zgc:63676	10
zgc:64105	10
zgc:65871	10
zgc:66450	10
zgc:77294	10
zgc:85696	10
zgc:92345	10
zgc:92664	10
zgc:92810	10
actr8	10
amt	10
bcap31	10
cdc2l1	10
cox6a1	10
creb3l3	10
dcp1a	10
ddx20	10
ddx51	10
dhx37	10

dmap1	10
faf1	10
fancc	10
ifrd2	10
kdm5bb	10
klhl12	10
LOC100004439	10
LOC558402	10
LOC567448	10
LOC568307	10
mak16	10
mknk2b	10
ogdh	10
osbpl9	10
piwil1	10
pola2	10
pop5	10
ppih	10
pqbp1l	10
prkab1b	10
ptp1b	10
rer1	10
si:ch211-147a11.7	10
si:ch211-218o21.2	10
snx2	10
t54l	10
tmed7	10
trim33	10
ufd1l	10
wdr46	10
wdr8	10
ythdf1	10
zgc:100970	10
zgc:101801	10
zgc:109953	10
zgc:109995	10
zgc:110709	10
zgc:112450	10
zgc:113491	10
zgc:152830	10
zgc:152874	10
zgc:154064	10
zgc:158399	10
zgc:158787	10
zgc:162485	10
zgc:171428	10

zgc:172291	10
zgc:56525	10
zgc:63688	10
zgc:73100	10
zgc:92140	10
zgc:92567	10
zgc:92668	10
aaas	10
actr5	10
acvr2a	10
agps	10
arglu1a	10
cask	10
ccnt2	10
chn1	10
cops8	10
dirc2	10
dync1i2a	10
eaf2	10
ercc1	10
esd	10
etv5	10
fzd8c	10
gdap2	10
hnmt	10
itgav	10
LOC570043	10
med14	10
mitd1	10
mmadhc	10
ndufa10	10
ormdl1	10
pdxkl	10
piga	10
rab20	10
rab9a	10
rftn2	10
si:ch211-194d6.2	10
si:dkey-189g17.2	10
si:dkey-22a1.3	10
si:dkey-253a1.3	10
sp3	10
spopl	10
stam2	10
tfg	10
tmem177	10

tra2b	10
txndc9	10
usp9	10
wdr33	10
zgc:101644	10
zgc:101659	10
zgc:153047	10
zgc:153086	10
zgc:153408	10
zgc:153683	10
zgc:158242	10
zgc:162345	10
zgc:171429	10
zgc:55283	10
zp2l1	10
ing2	11
zgc:101616	11
zgc:101819	11
zgc:112980	11
zgc:73273	11
zgc:77126	11
fbxw11b	11
phax	11
ppp2cb	11
sod1	11
vldlr	11
zgc:64133	11
zgc:110655	11
zgc:112296	11
zgc:92139	11
csnk1db	11
osbpl7	11
zgc:152977	11
zgc:85752	11
zgc:92313	11
c1d	11
vdac2	11
zgc:55819	11
dusp1	11
hmmr	11
med7	11
rmnd5b	11
stka	11
thoc3	11
pitpna	11
rpa1	11

zgc:55413	11
eif3ea	11
mcl1b	11
zgc:114060	11
zgc:153017	11
zgc:66195	11
zgc:66488	11
zgc:86599	11
kif11	11
rcor1	11
mibp2	11
pdcd5	11
pkm2a	11
si:dkey-238c7.16	11
wee2	11
zgc:123303	11
zgc:66107_dup1	11
zgc:66107_dup2	11
cdca8	11
LOC554876	11
LOC561143	11
mcl1a	11
rbb4	11
si:ch211-197n10.2	11
si:dkey-208k4.2	11
snx10a	11
txlna	11
zgc:110239	11
zgc:113424	11
zgc:56310	11
exosc4	11
mcm6l	11
prkacb	11
zgc:101744	11
zgc:66414_dup2	11
zgc:92744	11
dtl	11
glrx5	11
nek2	11
ociad1	11
pinx1	11
rpl7l1	11
si:ch211-150c22.2	11
ywhaqa	11
zgc:56703	11
zgc:92136	11

cct6a	11
pgk1	11
calr	11
rrbp1	11
si:ch211-250e5.16	11
zgc:153327	11
phc2	11
ssr4	11
uba1	11
zgc:114123	11
zgc:153867	11
zgc:55673	11
phex	11
zgc:56653	11
cat	11
cox5aa	11
cyp11a1	11
zgc:113028	11
arl6ip1	11
ppp4ca	11
ube2i	11
zgc:153952	11
atp5c1	11
caprin2	11
eif1axa	11
gldc	11
npm2	11
si:ch211-283h6.1	11
tmem93	11
tmprss4b	11
zgc:55461	11
ahcy	11
c20orf45	11
eif6	11
rtn4a	11
ruvbl1	11
sec61al2	11
tatdn2	11
zgc:100912	11
zgc:123190	11
got2b	11
krcp	11
orc6l	11
rbpms2	11
shcbp1	11
slc39a13	11

tipin	11
uqcrfs1	11
zgc:56231	11
zgc:77702	11
atp5f1	11
cdc14b	11
elovl7a	11
mapre1l	11
rab35	11
zgc:100919	11
zgc:55855	11
atp5o	11
cox17	11
retsatl	11
wdr75	11
zc3h15	11
zgc:153499	11
zgc:55512	11
zgc:55702	11
zgc:65979	11
abca1a	12
abcg2d	12
acsl1	12
acta1a	12
acy3.2	12
aip	12
arl11	12
arl13b	12
asb5	12
atp1a1a.2	12
atp1a1a.3	12
atp1a1a.4	12
atp1a1a.5	12
b4galt1	12
c3b	12
camk2d2	12
cbsa	12
cdadc1	12
cdc25d	12
chrnb3a	12
coro2a	12
crmp1	12
cryzl1	12
cyp2u1	12
cyp3a65	12
cyyr1	12

dachb	12
dachc_dup1	12
dla	12
dlx2b	12
dnm2l	12
ebpl	12
ednra	12
efnb2b	12
eng1b	12
erbb4	12
faah2a	12
fat1	12
fbxl3a	12
fga	12
fgb	12
fgf20a	12
fn1b	12
frem3	12
gefiltin	12
glb1	12
gpm6ab	12
gpm6ba	12
gpsn2	12
gtf2f2	12
hand2	12
helt	12
hmx4	12
il15	12
irx3b	12
itga6b	12
junb	12
kal1a	12
kctd12.2	12
kitb	12
klf12a	12
klf3	12
klf7	12
kpna3	12
lancl1	12
lbx1a	12
ldb2b	12
lef1	12
lgi2b	12
LOC100000151	12
LOC100001558	12
LOC100002676	12

LOC100003903	12
LOC100004819	12
LOC100005086	12
LOC100149185	12
LOC553253	12
LOC555748	12
LOC557028	12
LOC557257	12
LOC557882	12
LOC559126	12
LOC559804	12
LOC559976	12
LOC560006	12
LOC560319	12
LOC561940	12
LOC563273	12
LOC564697	12
LOC566247	12
LOC567248	12
LOC567859	12
LOC568915_dup1	12
LOC568915_dup2	12
LOC571908	12
LOC571915	12
LOC572312	12
LOC794323	12
LOC795258	12
LOC796649	12
LOC797213	12
mbnl2	12
med19b	12
meis4.1a	12
MGC174761	12
mhc1ze	12
mtmr7b	12
mtnr1aa	12
mxa	12
mylz3	12
nrp2a	12
odz3	12
pax5	12
pcdh10a	12
pcdh18a	12
pdlim3a	12
pgm2	12
pkd2	12

pmm2		12
prkcb1l		12
prom1b_dup1		12
psen2		12
qdprb1		12
qdprb2		12
rad23a		12
radil		12
rgs12		12
rnf11		12
rnf24		12
sc:d0144		12
sc4mol		12
	3-Sep	12
serhl		12
sfrp2		12
sgms2		12
si:busm1-105l16.2		12
si:ch211-214c7.5		12
si:ch211-217k17.10		12
si:ch211-241e15.2		12
si:ch211-281g13.2		12
si:dkey-1h24.2		12
si:dkey-245p14.4		12
si:dkey-7a20.6		12
si:dkey-82d4.2		12
sid4		12
slc25a10		12
slc25a4		12
slc34a2a		12
slc34a2aas		12
slit1b		12
slit2		12
smad1		12
smcr8b		12
smox		12
sox1b		12
ssp2		12
tctp		12
tll1		12
tlr2		12
trpc5		12
tuba8l2		12
tusc3		12
uncx4.1		12
vegfc		12

vps8	12
xpa	12
zgc:100814	12
zgc:100994	12
zgc:101855_dup1	12
zgc:101855_dup2	12
zgc:109983	12
zgc:110549	12
zgc:110664	12
zgc:110667	12
zgc:111946	12
zgc:112300	12
zgc:113099	12
zgc:123166	12
zgc:123242	12
zgc:123250	12
zgc:123318	12
zgc:136472	12
zgc:136584	12
zgc:136614	12
zgc:136652	12
zgc:136758	12
zgc:136908	12
zgc:136933	12
zgc:152698	12
zgc:152952	12
zgc:153020	12
zgc:153256	12
zgc:153638	12
zgc:153651	12
zgc:153720	12
zgc:153860	12
zgc:153894	12
zgc:153922	12
zgc:153928	12
zgc:153996	12
zgc:154024	12
zgc:158183	12
zgc:158288	12
zgc:158645	12
zgc:158803	12
zgc:162119	12
zgc:162208	12
zgc:162327	12
zgc:162825	12
zgc:162848	12

zgc:163136	12
zgc:165343	12
zgc:165520	12
zgc:165621	12
zgc:165626	12
zgc:171517	12
zgc:171773	12
zgc:171922	12
zgc:172216	12
zgc:173915	12
zgc:175214	12
zgc:193686	12
zgc:194578	12
zgc:194873_dup1	12
zgc:194873_dup2	12
zgc:194906	12
zgc:195282	12
zgc:55398	12
zgc:56295	12
zgc:56306	12
zgc:56409	12
zgc:63660	12
zgc:63691	12
zgc:63948	12
zgc:64012	12
zgc:64115	12
zgc:65888	12
zgc:66026	12
zgc:66080	12
zgc:76872	12
zgc:77076	12
zgc:77358	12
zgc:77636	12
zgc:77715	12
zgc:85727	12
zgc:85900	12
zgc:85981	12
zgc:91856	12
zgc:92115	12
zgc:92169	12
acads	12
agpat2	12
alcam	12
bach1	12
bmp1b	12
bsx	12

cadm2a	12
cnih2	12
cntfr	12
dbh	12
dclk1	12
dcp2	12
dgat2	12
dgcr2	12
dlg2_dup1	12
dlg2_dup2	12
dpysl2	12
dscam	12
epb4.1l4	12
erg	12
fez1	12
fgf13l	12
fgfr1b	12
foxo1b	12
frem2a	12
glis3	12
hdac4	12
il11ra	12
il1b	12
isl1l	12
isoc1	12
katnal2	12
kcnip3	12
kctd7	12
kdm6b	12
lhfp	12
LOC556326	12
LOC567731	12
lox	12
loxl2a	12
mef2ca_dup1	12
mef2ca_dup2	12
minal	12
mmp13	12
mogat2	12
morn5	12
mpx	12
musk	12
nbea	12
nfil3	12
nr2f1b	12
nrg1	12

nt5c2l1	12
or105-1	12
otx1l	12
paqr3b	12
pcdh1a3	12
pcdh1g22	12
pcdh1gb2	12
per1a	12
pigp	12
pip5k1b	12
pknox2	12
pnx	12
prkab1a	12
psmg1	12
purb	12
rasgef1bb	12
rassf6	12
rfx3	12
rhof	12
rilpl1	12
ripply3	12
rnasekb	12
robo3	12
samsn1b	12
sc:d0254	12
scpp8	12
sept5b	12
setd1ba	12
sfrp1b	12
si:busm1-57f23.1	12
si:busm1-79m10.4	12
si:ch211-237l4.5	12
si:ch211-243g18.2	12
si:xx-bac7cse.2	12
sim2	12
slc5a1	12
slc9a6b	12
smad9	12
smynd4	12
snx18b	12
sprn2	12
styxl1	12
syk	12
synj1	12
tlx3b	12
tmem126a	12

tmem88a	12
tuba7l	12
vamp2	12
vcanb	12
wnt11r	12
wu:fj39h09	12
zgc:101123	12
zgc:103419	12
zgc:103611	12
zgc:103654	12
zgc:109753	12
zgc:110011	12
zgc:110140	12
zgc:110200	12
zgc:110567	12
zgc:111821	12
zgc:112118	12
zgc:112138	12
zgc:113255	12
zgc:113274	12
zgc:113625	12
zgc:123275	12
zgc:136254	12
zgc:136455_dup1	12
zgc:136455_dup2	12
zgc:136807	12
zgc:136871	12
zgc:152845	12
zgc:152921	12
zgc:153423	12
zgc:158494	12
zgc:158652	12
zgc:158795	12
zgc:162139	12
zgc:162320	12
zgc:162322	12
zgc:162926	12
zgc:165474	12
zgc:171630	12
zgc:171684	12
zgc:172059	12
zgc:172243	12
zgc:172339	12
zgc:174237	12
zgc:194409	12
zgc:195147	12

zgc:55871	12
zgc:56039	12
zgc:63675	12
zgc:64196	12
zgc:77101	12
zgc:86759	12
zgc:91855	12
zgc:92034	12
zgc:92901	12
zswim6	12
ace2	12
acot8	12
alp	12
arrdc2	12
atp2b2	12
barx1	12
bhlhe40	12
btf3l4_dup1	12
btf3l4_dup2	12
cacna1d	12
camkv	12
cant1b	12
cdc42_dup2	12
cntn2	12
csf1a	12
ctns	12
cyb561d1	12
cybb	12
dhrs3b	12
dusp7	12
elk4	12
etnk2_dup1	12
etnk2_dup2	12
figf	12
gamt	12
gata2a	12
gdf11	12
gli2b	12
glt8d1	12
grma	12
hoxc12b	12
hoxc13b	12
igsf21a	12
im:7152789	12
irf2bp2	12
itih3	12

krml2	12
krml2.2	12
lgsn	12
LOC100150352	12
LOC560032	12
mafg2	12
map1lc3a	12
mapkapk3	12
matn4	12
mobkl2c	12
mxra8a	12
ndrg3a	12
nid1b	12
nlgn1_dup1	12
nlgn1_dup2	12
nr0b1	12
nr2f6b	12
olfml3	12
opn1lw2	12
opn1sw2	12
or134-1	12
or137-3	12
pax7a	12
pecr	12
pir	12
prph	12
rargb	12
reep6	12
setmar	12
sfrs7	12
si:busm1-136d19.2	12
si:busm1-6a2.1_dup	12
si:busm1-6a2.1_dup	12
si:ch211-15p9.2	12
si:dkey-13a21.14	12
si:dkey-13a21.3	12
si:dkey-13a21.4	12
si:dkey-13a21.9	12
sid1	12
silva_dup1	12
silva_dup2	12
skib	12
slc8a1a	12
socs8	12
sulf2	12
tceb3	12

tdo2b	12
tmem38a	12
tuba2	12
uox	12
vamp3	12
vsg1	12
xk	12
zgc:101030	12
zgc:101737	12
zgc:103663	12
zgc:110191	12
zgc:110286	12
zgc:110312	12
zgc:110548	12
zgc:110683	12
zgc:111961	12
zgc:112199	12
zgc:112265	12
zgc:112519	12
zgc:113184	12
zgc:113223	12
zgc:113276	12
zgc:113307	12
zgc:114079	12
zgc:136902	12
zgc:153629	12
zgc:153726	12
zgc:153764_dup1	12
zgc:153764_dup2	12
zgc:153953	12
zgc:153976	12
zgc:153987	12
zgc:154141	12
zgc:158258	12
zgc:158271	12
zgc:158274	12
zgc:158292	12
zgc:158299	12
zgc:158340	12
zgc:158635	12
zgc:158653	12
zgc:158773	12
zgc:162129	12
zgc:162182	12
zgc:162738	12
zgc:165463	12

zgc:165500	12
zgc:165543	12
zgc:171580	12
zgc:171923	12
zgc:172085	12
zgc:172180	12
zgc:172352	12
zgc:173545	12
zgc:175135	12
zgc:193807	12
zgc:194395	12
zgc:194937	12
zgc:195317	12
zgc:63629	12
zgc:64002	12
zgc:64054	12
zgc:64194	12
zgc:77375	12
zgc:86889	12
zgc:91942	12
zgc:92715	12
aarsd1	12
acta2_dup1	12
acta2_dup2	12
adrb1	12
aldoab	12
anxa11b	12
aqp8a	12
atad4b	12
atp2a1l	12
bhlha15	12
cbx1b	12
ch25h	12
chac2	12
chad	12
col1a3	12
cpn1	12
cpped1	12
crygmx	12
csdc2	12
csnk1e	12
dkk1	12
dlx3b	12
dpysl3	12
eef2k	12
entpd1	12

erbb2	12
exoc6	12
foxi1	12
foxj1b	12
fscn1l	12
g6pca	12
ghitm_dup2	12
ghrhr2	12
ghrhrl	12
gprc5c	12
gspt1	12
heatr1	12
hoxb1b	12
hoxb5b	12
hs3st3b1b	12
im:6895556	12
irf3	12
irf9	12
kdelr2l	12
ldb3b	12
lgi1b	12
LOC562651	12
LOC797338	12
lrrc20	12
mapk8	12
mkxa	12
mpp2b	12
mrpl10	12
narf	12
ndel1a	12
ndr2	12
neurod2	12
nploc4	12
nrxn1a	12
p4ha2	12
papss2	12
pcdh15b	12
pde6c	12
pde6g	12
pigf	12
plce1	12
pmp22b	12
psme2	12
pth1rb	12
pts	12
pvalb2	12

pvalb9	12
rap2ip	12
raraa	12
rbp4	12
rds2	12
rnls	12
scd	12
scn4aa	12
si:ch211-146f4.4	12
si:ch211-147h1.3	12
si:ch211-208d15.4	12
si:dkey-220f10.6	12
six3b	12
skap1	12
slc16a12b	12
slc16a9b	12
sox9a	12
sult2st1	12
sult2st2	12
tcf7l2	12
timp2	12
ttll6	12
ttyh2	12
valopb	12
wu:fj49c01	12
zfhx1	12
zgc:101815	12
zgc:101843_dup1	12
zgc:101843_dup2	12
zgc:103639	12
zgc:103767	12
zgc:109930	12
zgc:110030	12
zgc:110789	12
zgc:112145	12
zgc:112176	12
zgc:112954	12
zgc:113062	12
zgc:113325	12
zgc:113574	12
zgc:123217	12
zgc:136337	12
zgc:153278	12
zgc:153595	12
zgc:153947	12
zgc:158425	12

zgc:158782	12
zgc:158843	12
zgc:162187	12
zgc:162235	12
zgc:162915	12
zgc:165653	12
zgc:165670	12
zgc:171485	12
zgc:171544	12
zgc:171664	12
zgc:171713	12
zgc:171763	12
zgc:171853	12
zgc:172061	12
zgc:172248	12
zgc:172258	12
zgc:174622	12
zgc:174690	12
zgc:175131	12
zgc:175209	12
zgc:175222	12
zgc:194281	12
zgc:194501	12
zgc:194737	12
zgc:56272	12
zgc:63934	12
zgc:65890	12
zgc:73060	12
zgc:73134	12
zgc:73189	12
zgc:73259	12
zgc:86738	12
zgc:86776	12
zgc:91912	12
zgc:91926	12
zgc:92083	12
zgc:92648	12
zgc:92749	12
zgc:92858	12
znf668	12
zplxdc1	12
abcc2	12
abcg2c	12
acta1b	12
adam8a	12
adh8a	12

adss	12
arg2	12
atl1	12
b3galnt2	12
bag3	12
bcl11a	12
bcl2l11	12
btbd9	12
c10orf11	12
calhm2	12
capn1a	12
capn2a_dup1	12
capn2a_dup2	12
ccdc85a	12
cdh23	12
cdkl1	12
ckba	12
cox16	12
ctsh	12
cutc	12
cxcl12a	12
dap1b	12
degs1	12
dhrs7	12
disc1	12
dld	12
dlg5	12
dync2li1	12
eif2ak2	12
elovl5	12
emx1	12
emx2	12
epas1	12
esr2b	12
fancb	12
fbln5	12
fbxo9	12
fgfr2_dup1	12
fgfr2_dup2	12
fgfr2_dup3	12
fgfr3	12
glo1	12
gng2	12
gstal	12
gtpbpl_dup2	12
hk1	12

hkdc1	12
ikzf1	12
irf1	12
klhl31	12
kmo	12
lbx1b	12
ldb3a	12
ldlrap1b	12
LOC100002257	12
LOC100002333	12
LOC100002616	12
LOC100003801	12
LOC100149164	12
LOC100149820	12
LOC555251	12
LOC556058	12
LOC559814	12
LOC561206	12
LOC565246	12
LOC565375	12
LOC565426	12
LOC565716	12
LOC566435_dup2	12
LOC567812	12
LOC568087	12
LOC570926	12
LOC571658	12
LOC792757	12
LOC796680	12
LOC796750	12
LOC799537_dup1	12
LOXL3	12
loxl3b	12
lrit1	12
lrrc9	12
mat1a	12
matn3b	12
meis1	12
mlh1	12
morn4	12
mrpl14_dup1	12
msxc	12
mxtx1	12
nfkb2	12
nkx1.2la	12
nkx6.2	12

nrxn1b	12
nt5c2	12
olig4	12
oprm1	12
osr1	12
park2	12
pcdh15a_dup1	12
pcdh15a_dup2	12
pcdh21	12
pcsk2_dup2	12
pde10a	12
pitx3	12
pkz	12
pla2g12b_dup1	12
pla2g12b_dup2	12
plek2	12
pqlc3	12
prdm8	12
prkg1	12
rab4a	12
rcl	12
rdh12	12
rds4	12
rel	12
ret1	12
rh30	12
rhoua	12
rrh	12
rtn1a	12
runx3	12
scdb	12
sertad2	12
sft2d1	12
si:busm1-265n4.4	12
si:ch211-206k20.4	12
si:ch211-217g15.2	12
si:ch211-8a9.3	12
si:dkeyp-2e4.6	12
six2.1	12
six3a	12
six4.2	12
six6a	12
slc1a4	12
slc25a16	12
slc2a15a	12
slc30a10	12

slc4a11	12
slc8a3	12
slit1a	12
spred2	12
srfl	12
tlr4a	12
tlr4b	12
tlx1	12
trim9	12
ubtd1	12
unc5b	12
vcl	12
zbtb8os	12
zgc:101572	12
zgc:110105	12
zgc:110146_dup1	12
zgc:110197	12
zgc:110784	12
zgc:110796	12
zgc:111947	12
zgc:113197	12
zgc:113291	12
zgc:113956	12
zgc:114051	12
zgc:123068	12
zgc:123251	12
zgc:123267	12
zgc:123282	12
zgc:136953	12
zgc:153049	12
zgc:153073	12
zgc:153142	12
zgc:153169	12
zgc:153442	12
zgc:153588	12
zgc:153981	12
zgc:154058	12
zgc:154100	12
zgc:158392	12
zgc:158667	12
zgc:162303	12
zgc:162640	12
zgc:162982	12
zgc:163001	12
zgc:163054	12
zgc:171495	12

zgc:171534	12
zgc:171814	12
zgc:172124	12
zgc:172136	12
zgc:172321	12
zgc:194817	12
zgc:56340	12
zgc:63561	12
zgc:64043	12
zgc:64201	12
zgc:65960_dup2	12
zgc:66298_dup1	12
zgc:66298_dup2	12
zgc:92254	12
zgc:92261	12
zgc:92420	12
zgc:92501	12
zgc:92714	12
zgc:92799	12
zmiz1	12
anxa6	12
arhgef9	12
atoh8	12
bbs7	12
casp3b	12
cdx1a	12
cdx4	12
chrnb3b	12
cryba1l	12
csnk1a1_dup1	12
csnk1a1_dup2	12
ctsf	12
cxcl14	12
ddx26b	12
drp2	12
efemp2	12
egf	12
elovl6	12
emx3	12
enpp6	12
f9	12
fgf1	12
fgf13	12
fgf18a	12
fgf20b	12
fgf24	12

fgfrl1a	12
fgfrl1b	12
fhla	12
flt4	12
fosl1	12
fstl5	12
gabra6a	12
gabrb2	12
gdf9	12
glra1	12
glrbb	12
gpm6aa	12
gria1a	12
gria3b	12
her11	12
hnrnph1	12
hopx	12
igfbp7	12
il1rapl2_dup1	12
il1rapl2_dup2	12
il2rgb	12
insb	12
irf2	12
itk	12
itln3	12
kazald2	12
kctd12b	12
kctd8	12
kdrl	12
lbx2	12
ldb2a	12
limch1	12
mbnl3	12
med12_dup2	12
msxa	12
msxe	12
mtus1a	12
neurog1	12
nkx2.5	12
nkx3.2	12
nox1	12
otop1	12
pcdh10b	12
pcdh18b	12
pcdh2ab10_dup1	12
pcdh2ab11_dup1	12

pcdh2ab12	12
pcdh2ab2	12
pcdh2ab3	12
pcdh2ab6	12
pcdh2ab7	12
pcdh2ab8	12
pcdh2g17_dup1	12
pde6a	12
pitx2	12
plp1a	12
pls3	12
pmaip1	12
ppp2ca	12
rtn4rl2b	12
setd7	12
sfxn1	12
slc25a43	12
slc25a5_dup1	12
slc25a5_dup2	12
slc7a2	12
slit3	12
sox21b	12
spata4	12
spon2a	12
spon2b	12
spry1	12
tbx22	12
tdo2a	12
tgfbi	12
tlx2	12
tmem35	12
trim35	12
tsc22d3	12
ube2a_dup1	12
ube2a_dup2	12
zgc:101848	12
zgc:110017	12
zgc:110141	12
zgc:110376	12
zgc:110421	12
zgc:110426	12
zgc:110560	12
zgc:110843_dup2	12
zgc:112315	12
zgc:112431	12
zgc:112531	12

zgc:113229	12
zgc:113271	12
zgc:113342	12
zgc:113364	12
zgc:114084_dup1	12
zgc:114084_dup2	12
zgc:114137	12
zgc:136458	12
zgc:136859	12
zgc:136890	12
zgc:136936	12
zgc:152778	12
zgc:153146	12
zgc:154020	12
zgc:154040	12
zgc:154042	12
zgc:158426	12
zgc:158483	12
zgc:162144	12
zgc:162298	12
zgc:162893	12
zgc:171223	12
zgc:171591	12
zgc:171626	12
zgc:172101	12
zgc:172264	12
zgc:173617_dup2	12
zgc:173703	12
zgc:174864	12
zgc:175125	12
zgc:175202	12
zgc:193751_dup2	12
zgc:194297	12
zgc:194528	12
zgc:195063	12
zgc:63528	12
zgc:63568	12
zgc:64101	12
zgc:64130	12
zgc:66447	12
zgc:77513	12
zgc:77906	12
zgc:77938	12
zgc:86674	12
zgc:91894	12
zgc:92090	12

zgc:92093	12
zgc:92242	12
zgc:92249	12
zgc:92608	12
zgc:92762	12
zgc:92812	12
zgc:92907	12
zic3	12
abcg4b	12
acpl2	12
adfp	12
atxn3	12
bbc3	12
bco2a	12
cadm1b	12
cadm2b	12
chd	12
ckmb	12
cldn8	12
cldna	12
cldnf	12
clrn1	12
crfb7	12
cryba1a	12
cwf19l2	12
dharma	12
dhrs11b	12
dixdc1	12
dlc	12
dpf1	12
drd2a_dup1	12
drd2a_dup2	12
foxn1	12
frem2b	12
ftr72	12
gap43	12
gdpd1	12
gip	12
gucy2f	12
her13	12
hic1	12
hnf1b	12
hsp47	12
hspa13	12
ift20	12
il15l	12

il1rapl2	12
itm2ca	12
kcnj13	12
kirrel3	12
lim2.3	12
limk1	12
LOC100005599	12
LOC559844	12
lsamp	12
mab21l1	12
mag	12
mcam	12
meis3	12
mff	12
mtnr1ba_dup2	12
ncam3	12
nek8	12
nlcam	12
nos2b	12
nphs1l	12
odz4	12
or107-1	12
or111-11	12
or127-1	12
otx5	12
p2rx8	12
pdk3b	12
phox2a	12
picalml	12
postn	12
prcp	12
rab30	12
rab34	12
rhoub	12
rnmtl1	12
robo2	12
sag	12
samsn1a	12
sarm1	12
sc:d136	12
scn3b	12
sgcg	12
shox2	12
si:ch211-113p18.5	12
si:dkey-24p1.1	12
si:dkey-24p1.4	12

slc37a4	12
slc43a2	12
slc6a4a	12
smtnl	12
tbx2b	12
tbx4	12
tgfb1	12
tmem195	12
tmprss13a	12
tnfb	12
tnfrsf19	12
traf4a	12
tyr	12
vtna	12
wrb	12
wu:fb97d07	12
wu:fc20e06	12
wu:fc58a04	12
zgc:100896	12
zgc:101731	12
zgc:101740	12
zgc:101746	12
zgc:101797	12
zgc:103612	12
zgc:103681	12
zgc:109985	12
zgc:110293	12
zgc:110585	12
zgc:110692	12
zgc:112020	12
zgc:112083_dup1	12
zgc:112083_dup2	12
zgc:113279	12
zgc:113294	12
zgc:113423	12
zgc:114034	12
zgc:114148	12
zgc:123007	12
zgc:123187	12
zgc:123204	12
zgc:136739	12
zgc:136925	12
zgc:153009	12
zgc:153032	12
zgc:153120_dup1	12
zgc:153164	12

zgc:153184	12
zgc:153235	12
zgc:153310	12
zgc:153372	12
zgc:153454	12
zgc:153498	12
zgc:153721	12
zgc:153766	12
zgc:153898	12
zgc:154151	12
zgc:158328	12
zgc:158686	12
zgc:158707	12
zgc:162193	12
zgc:162197	12
zgc:162271	12
zgc:162608	12
zgc:162730	12
zgc:162872	12
zgc:162883	12
zgc:162936	12
zgc:162985	12
zgc:165502	12
zgc:165649	12
zgc:171582	12
zgc:171845	12
zgc:171927	12
zgc:172282	12
zgc:173444	12
zgc:174574_dup2	12
zgc:174895	12
zgc:195004	12
zgc:56201	12
zgc:63634	12
zgc:63663	12
zgc:63670	12
zgc:64103	12
zgc:65827	12
zgc:66101	12
zgc:73220	12
zgc:73371	12
zgc:73377	12
zgc:77409	12
zgc:77784	12
zgc:85932	12
zgc:86870_dup1	12

zgc:91858	12
zgc:92134	12
zgc:92326_dup2	12
zgc:92349	12
zgc:92869	12
a2bp1l	12
acbd7	12
ahr1a	12
aldh5a1	12
apoa4	12
arnt	12
aste1	12
bcan	12
cadm4	12
casp8l1	12
caspa	12
ccbe1	12
cd4-4	12
cdh17	12
ceacam1	12
cep76	12
cnot3a	12
crabp2a	12
csf3r	12
ctsk	12
ctssb.2	12
cx55.5	12
cxcr3.1	12
cxcr3.2	12
cyp11b2	12
cyp4	12
dbf4	12
depdc6	12
dfna5	12
efna3b	12
enpp2	12
entpd3	12
epb41	12
etv2	12
flad1	12
gapdhs	12
gdf6a	12
gem	12
gnb3l	12
gnl2	12
gpn2	12

hacl1	12
hapln2	12
hcst	12
hdac9b	12
herpud2	12
hfe2	12
hoxa13b	12
icn	12
im:7143992	12
invs	12
irge4	12
irx1a	12
irx2a	12
isg20l2	12
isoc2	12
kiaa0907	12
leng1	12
lmna	12
LOC100006927	12
LOC100149386	12
LOC559008	12
LOC559074	12
LOC563200	12
LOC572950	12
LOC791982	12
LOC799527	12
mc2r	12
mc5rb	12
me1	12
mecr	12
med18	12
mef2d	12
mylipb	12
nr2f5	12
onecutl	12
paqr7b	12
pard6ga	12
pex5_dup2	12
pklr	12
popdc1	12
pou47	12
pou50	12
ppox	12
prdm1a	12
prss35	12
psmd4	12

ptpn2	12
ptpn6	12
ptpru	12
rad21_dup1	12
rad21_dup2	12
rag1ap1	12
rbms3	12
rbp1a	12
rgl2	12
rhbg	12
rpz	12
rspo1	12
s100a10b	12
s100t	12
scn1ba	12
si:ch211-154o6.3	12
si:ch211-154o6.4	12
si:ch211-154o6.6	12
si:ch211-154o6.7	12
si:ch211-69g19.2	12
si:dkey-11p23.3	12
si:dkey-33i11.3	12
sim1	12
skap2	12
slc25a32b	12
slc39a3	12
slc6a19	12
slc6a3	12
snip1	12
sox4b	12
sp8l	12
stmn1b	12
stmn2a	12
sult2st3	12
syt11b	12
taf12_dup1	12
taf12_dup2	12
tbx20	12
tcea3	12
tgfb2	12
thbs3a	12
tlr18	12
tmod4	12
tnfaip8l	12
tnfrsf1a	12
try	12

ttc35_dup1	12
twist1b	12
tyrobp	12
upp1	12
vegfaa	12
zfpm2a	12
zgc:101640	12
zgc:101891	12
zgc:103778	12
zgc:110131	12
zgc:110324_dup1	12
zgc:110385	12
zgc:110410	12
zgc:110579	12
zgc:110643	12
zgc:110662	12
zgc:110759	12
zgc:112009	12
zgc:112037	12
zgc:112374	12
zgc:113199	12
zgc:113320	12
zgc:113646	12
zgc:123214	12
zgc:136803	12
zgc:136947	12
zgc:152945	12
zgc:153234	12
zgc:153288	12
zgc:153691	12
zgc:153955	12
zgc:154065	12
zgc:158151	12
zgc:158394	12
zgc:158431	12
zgc:158657	12
zgc:158741	12
zgc:158866	12
zgc:162040	12
zgc:162150	12
zgc:162160	12
zgc:162212	12
zgc:162213	12
zgc:162509	12
zgc:162582	12
zgc:165409	12

zgc:165534	12
zgc:171577	12
zgc:171704	12
zgc:171801	12
zgc:172086	12
zgc:172096_dup1	12
zgc:172096_dup2	12
zgc:172149	12
zgc:172162	12
zgc:172265	12
zgc:172323	12
zgc:174637	12
zgc:174888	12
zgc:174938	12
zgc:175182_dup1	12
zgc:175182_dup2	12
zgc:193796	12
zgc:194131	12
zgc:194210	12
zgc:194761	12
zgc:195265	12
zgc:63489	12
zgc:73062	12
zgc:73341	12
zgc:77556	12
zgc:91835	12
zgc:91887	12
zgc:92129	12
zgc:92327	12
zgc:92467	12
zgc:92763	12
acta1	12
actn2	12
adssl1	12
ahi1	12
ankrd9	12
ap4s1	12
bmp4	12
ccdc85ca	12
cfl2	12
chrna2a	12
chst10	12
col10a1	12
colec11	12
crim1	12
ctsl.1	12

cx35.4	12
cyp26c1	12
dio2	12
dnajc27	12
dnmt6	12
dpysl5a	12
drd4a	12
dtk	12
dtnb	12
dync1h1	12
egln3	12
egr2a	12
eml1	12
ephx2	12
esrrg	12
evlb	12
fabp7a	12
fam98b	12
foxa1	12
foxa2	12
foxg1	12
fut8	12
fzd3l_dup1	12
fzd3l_dup2	12
gchfr	12
gnrh3	12
hhat	12
hmx2	12
hmx3	12
htra1	12
il17a/f2	12
insm1b	12
k2p10.1	12
khk	12
kif3c	12
klf11b	12
lck	12
lin52	12
LOC100000800	12
LOC100004628	12
LOC569164	12
LOC794087	12
lpin1	12
ltk	12
manea	12
meis2.2	12

mocs1	12
mocs3	12
myo6b	12
nkx2.2a	12
nkx2.9	12
nrxn3a	12
nubpl	12
numb	12
otx2	12
pabpc4	12
parvb	12
pax9	12
pcmtl	12
plekhh1	12
pomca	12
prdm5	12
prox1	12
prox2	12
psmc1a	12
qk_dup1	12
qk_dup2	12
rasgrp3	12
rbks	12
rcan3	12
reep3l	12
rgs7	12
rnf144aa	12
rsad2	12
rtn4ip1	12
sccpdha	12
sepn1	12
slc16a9a	12
slc25a21	12
slc25a29	12
snap25b	12
socs4	12
spata17	12
spred1	12
sptb	12
tgfb3	12
tmem206	12
ublcp1	12
vax1	12
vrk1	12
vsx1	12
vsx2	12

xgb	12
zgc:100951	12
zgc:101540	12
zgc:101720_dup1	12
zgc:101720_dup2	12
zgc:101772	12
zgc:101786	12
zgc:109986	12
zgc:110176	12
zgc:110294	12
zgc:112003	12
zgc:112375	12
zgc:113377	12
zgc:113886	12
zgc:114065	12
zgc:123120	12
zgc:123271	12
zgc:136571	12
zgc:136752	12
zgc:152746	12
zgc:153026	12
zgc:153098	12
zgc:153298	12
zgc:153353	12
zgc:153417	12
zgc:153596	12
zgc:153973	12
zgc:153974	12
zgc:154055	12
zgc:158347	12
zgc:158361	12
zgc:158466_dup1	12
zgc:158651	12
zgc:158781	12
zgc:162165	12
zgc:162184	12
zgc:162310	12
zgc:162576	12
zgc:162909	12
zgc:162913	12
zgc:163122	12
zgc:165461	12
zgc:165580	12
zgc:165628	12
zgc:165635	12
zgc:171416	12

zgc:171694	12
zgc:172202	12
zgc:173816	12
zgc:174342	12
zgc:174379	12
zgc:175176	12
zgc:193725	12
zgc:194201	12
zgc:194668	12
zgc:195062	12
zgc:195269	12
zgc:63633	12
zgc:77303	12
zgc:77799	12
zgc:85722	12
zgc:91908	12
zgc:92375	12
zgc:92542	12
zgc:92867	12
zgc:92902	12
acadl	12
ampd3	12
arpp19a	12
bbs10	12
bbs2	12
bcl9l	12
bhlhb3l	12
c11orf67	12
c3orf33	12
cbfb	12
cbln1	12
cd9	12
cdon	12
cetp	12
chka	12
chrna5	12
cpt1b	12
cyba	12
cyp19a1a	12
cyp1a	12
dclk2	12
dennd4a	12
dhdhl	12
dusp22a	12
ehd2	12
exosc5	12

fam168a	12
fli1a	12
foxl1	12
fzd9	12
gatm	12
gnao1a	12
gnb5	12
gnrhr4	12
gpib	12
grk7b	12
gyltl1b	12
has3	12
hipk2	12
ighmbp2l	12
irf8	12
kcnab1	12
kcnj1	12
kctd15	12
klhl36_dup1	12
klhl36_dup2	12
lctlb	12
leo1	12
lmo2	12
LOC100004247	12
LOC100150067	12
LOC402785	12
LOC560780	12
LOC564395	12
LOC565256	12
LOC566046	12
LOC569072	12
LOC570726	12
LOC571486	12
LOC572261	12
LOC798102	12
lysmd2	12
map2k1	12
mapk12	12
mef2a	12
mmp23al	12
mthfsd	12
myo5a	12
myo7a	12
nedd4a	12
nptn	12
nr1h4	12

nr2f2	12
odf3l	12
onecut1	12
parp6a	12
plcg2	12
pllp	12
ppcdc	12
prtga_dup1	12
prtga_dup2	12
relt	12
rfx4	12
rgma	12
sema3ab	12
sema3d	12
serpini1	12
si:ch211-122c9.1	12
si:ch211-132b12.1	12
si:ch211-132b12.3	12
si:ch211-132b12.7	12
si:ch211-151h10.2	12
si:ch211-15i6.4	12
si:ch211-160d20.1	12
si:ch211-200o3.4	12
si:ch211-203b8.6	12
si:ch211-218c6.1	12
si:ch211-219a15.3	12
si:ch211-238n5.4	12
si:ch211-239j19.3	12
si:ch211-245p7.3	12
si:ch211-260p9.6	12
si:ch211-278n20.6	12
si:dkey-103i16.2	12
si:dkey-10o6.2	12
si:dkey-12e7.4	12
si:dkey-246a16.2	12
si:dkey-246a16.3	12
si:dkey-246g23.3	12
si:dkey-246g23.4	12
si:dkey-24l11.1	12
si:dkey-24l11.2	12
si:dkey-24l11.5	12
si:dkey-24l11.8	12
si:dkey-24l11.9	12
si:dkey-25f3.3	12
si:dkey-261h15.1	12
si:dkey-265o13.4	12

si:dkey-270i2.2	12
si:dkey-30c15.2	12
si:dkey-30c15.5	12
si:dkey-7l12.1	12
si:dkeyp-109h9.2	12
si:dkeyp-1h4.3	12
si:dkeyp-59c12.1	12
slc12a1	12
slc12a3	12
slc17a8	12
slc27a2	12
slc8a2b	12
slco3a1	12
smad3b	12
sparcl	12
sphkap	12
st3gal2	12
tat	12
tbxas1	12
tph2	12
tspan18b	12
veph	12
wdr69	12
wnt2	12
yap1_dup1	12
yap1_dup2	12
zfpm1	12
zgc:100824	12
zgc:101575	12
zgc:101711	12
zgc:101723	12
zgc:101815_dup1	12
zgc:101815_dup2	12
zgc:103515	12
zgc:103600	12
zgc:110373	12
zgc:110594	12
zgc:112232	12
zgc:112407	12
zgc:113258	12
zgc:114141	12
zgc:123280	12
zgc:153031	12
zgc:153247	12
zgc:153270	12
zgc:153717	12

zgc:153863	12
zgc:154083	12
zgc:158279	12
zgc:158458	12
zgc:158464	12
zgc:158671	12
zgc:158868	12
zgc:161981	12
zgc:162606	12
zgc:162756	12
zgc:162898	12
zgc:162977_dup1	12
zgc:162977_dup2	12
zgc:171509	12
zgc:172214	12
zgc:175192	12
zgc:55318	12
zgc:56622	12
zgc:63658	12
zgc:66371	12
zgc:77182	12
zgc:77752	12
zgc:77853	12
zgc:91860	12
zgc:91911	12
zgc:92354	12
abcb3l1	12
adcy2b	12
agr2	12
angpt1	12
angpt2	12
arhgef1	12
arl4l	12
asb4	12
asns	12
atp1a3a	12
atxn1b	12
bty	12
ccdc127	12
clic1_dup1	12
clic1_dup2	12
col11a2	12
col1a2	12
col9a2	12
crabp2b	12
crtap	12

csf3	12
ctgf	12
cthrc1a	12
ctssa	12
cx39.4	12
cyt1	12
cyt1l	12
dgat1	12
dlx6a	12
dtnbp1b	12
edn1	12
efna1a	12
ensa	12
ercc5	12
evx1	12
ext1c	12
fabp11a	12
fam91a1	12
fkbp9	12
ftr56	12
gatad1	12
gdf6b	12
glcci1	12
gmpr	12
gnb3	12
gngt1	12
gpd1	12
grhl2	12
grnas	12
hey1	12
hoxa13a	12
hoxa9a	12
hpca	12
irx1b	12
irx4b	12
kat2b	12
LOC100000030	12
LOC100000126	12
LOC100002560	12
LOC100003866	12
LOC100006053	12
LOC100006223	12
LOC100148025	12
LOC100151049	12
LOC555593	12
LOC566764	12

LOC566830	12
LOC566969	12
LOC569167	12
LOC572161	12
LOC792465	12
LOC799704	12
mhc1uba	12
necab1	12
neu1	12
nfatc1	12
nkd2	12
nkd2a	12
npy	12
nr1d2b	12
nxph1	12
oxnad1	12
pabpc1b	12
plekho1	12
ppt2	12
psmb10	12
psmb11	12
psmb8	12
psmb9a	12
ptk2.2	12
rab25	12
s100u	12
s100v2	12
scn1bb	12
si:ch211-133n4.4	12
si:ch211-173p18.9	12
si:ch211-191i18.2	12
si:ch211-191i18.3	12
si:ch211-194e15.5	12
si:ch211-195b13.1	12
si:ch211-195k18.2	12
si:ch211-197n10.4	12
si:ch211-199g17.1	12
si:ch211-203l9.3	12
si:ch211-204j16.1	12
si:ch211-206a7.2	12
si:ch211-222e23.6	12
si:ch211-222e23.7	12
si:ch211-232m10.4	12
si:ch211-234f14.1	12
si:ch211-239d6.1	12
si:ch211-246f9.1	12

si:ch211-250g4.1	12
si:ch211-30b16.2	12
si:ch211-39a7.1	12
si:ch211-39e15.2	12
si:ch211-51f10.1	12
si:ch211-81a5.8	12
si:dkey-12h3.1	12
si:dkey-14o18.5	12
si:dkey-151c10.1	12
si:dkey-158b13.1	12
si:dkey-181o6.1	12
si:dkey-18f7.1	12
si:dkey-190l1.2	12
si:dkey-195c14.1	12
si:dkey-204a24.5	12
si:dkey-204a24.6	12
si:dkey-218f9.5	12
si:dkey-21p19.1	12
si:dkey-222b8.1	12
si:dkey-228a15.3	12
si:dkey-231l1.6	12
si:dkey-261i16.5	12
si:dkey-263h23.2	12
si:dkey-27c15.1	12
si:dkey-284p5.3	12
si:dkey-3n22.7	12
si:dkey-81h8.1	12
si:dkeyp-113d7.4	12
si:dkeyp-113d7.7	12
si:dkeyp-114f9.4	12
si:dkeyp-24a7.7	12
si:dkeyp-89d7.2	12
si:dkeyp-92c9.2	12
si:dkeyp-92c9.3	12
si:rp71-1h3.1	12
si:rp71-45k5.3	12
si:rp71-57j15.4	12
slc44a4	12
slc9a3.1	12
slc9a3.2	12
sox4a	12
sp8	12
spag1	12
srd5a1	12
stmn2b	12
tfpi2	12

thbs3b	12
them2	12
thrap6	12
thrb	12
tmem55a	12
tnrc4	12
top2b	12
tpm3_dup1	12
tpsn	12
trpn1	12
tspan13	12
twist1a	12
wdr51bl	12
yrk	12
zgc:100906	12
zgc:101005	12
zgc:101116	12
zgc:101684	12
zgc:101785_dup2	12
zgc:103438	12
zgc:109981	12
zgc:110075	12
zgc:112024	12
zgc:114147	12
zgc:123049	12
zgc:123347	12
zgc:136713	12
zgc:136926	12
zgc:153333	12
zgc:153728	12
zgc:153912	12
zgc:153914_dup1	12
zgc:153914_dup2	12
zgc:154043	12
zgc:154044	12
zgc:158376	12
zgc:158766	12
zgc:162972	12
zgc:171454	12
zgc:172108	12
zgc:174633	12
zgc:194294	12
zgc:63606	12
zgc:63774	12
zgc:64214	12
zgc:65893	12

zgc:73061	12
zgc:73075	12
zgc:73317	12
zgc:85777	12
zgc:91944	12
zgc:92005	12
zgc:92362	12
zgc:92476	12
zgc:92533	12
zgc:92739	12
zgc:92826	12
znf384l	12
abcd3b	12
abhd4	12
acot11	12
adcyap1b	12
agfg1b	12
agxt	12
ahrrb	12
amph	12
amy2a	12
ankhb	12
apol1	12
arhgap12a	12
arhgap29	12
atoh2b	12
atp1a2a	12
atp1b3a	12
ca8	12
cables1	12
cacnb2b	12
cadm3_dup1	12
cahz	12
capsl	12
cbln2a	12
ccdc24	12
cdc14aa	12
cdh10	12
cdh18	12
cdh6	12
cdh7	12
cldn10l	12
clstn2	12
cpla2	12
crfb16	12
crispld1	12

crlf1a	12
crygn1	12
cryz	12
dhrs1	12
dmbx1a	12
dpp6	12
dusp22b	12
efna2	12
egfr	12
eif2b3	12
ek3	12
eng2b	12
epha4a	12
epha4b	12
fam102ba	12
fbxo36	12
fgf12	12
fndc3ba	12
foxc1a	12
ftr11	12
gadd45al	12
gfi1.1	12
ghrhr	12
gng12	12
golim4b	12
gpt2l	12
hbl4	12
hhatla	12
hs6st1a	12
htr5a	12
igfbp1b	12
im:7153495	12
impa1	12
itgb1b.1	12
itgb1b.2	12
jph1b	12
jund	12
kbtbd5	12
kcnh2l	12
keap1a	12
klhl6	12
LOC100000849	12
LOC100002070	12
LOC100002496	12
LOC100003595	12
LOC100004736	12

LOC100005645	12
LOC100006444	12
LOC100006660	12
LOC100148828	12
LOC100148868	12
LOC100148976	12
LOC100170660	12
LOC554843	12
LOC557261	12
LOC561247	12
LOC561308	12
LOC563352	12
LOC565395	12
LOC568508	12
loxl5b	12
lpar1	12
lrrc6	12
mbnl1_dup1	12
mbnl1_dup2	12
mc4r	12
mgc35261l	12
mib	12
mmp14b	12
mpz	12
mul1	12
myom1a	12
nrp1b	12
opn4l	12
oprk1	12
otomp	12
pax3a	12
pbx1a	12
pcp4l1	12
per2	12
pigk	12
pls1	12
prrx1a	12
ptc1	12
ptgs2a	12
pth1ra	12
rbp2b	12
rgs20	12
ripk1l	12
rxrga_dup1	12
rxrga_dup2	12
sc:d185	12

scinlb	12
scn12aa	12
sema4e	12
shhb	12
si:ch211-106n13.1	12
si:ch211-106n13.3	12
si:ch211-119o8.5	12
si:ch211-14a17.13	12
si:ch211-160i2.3	12
si:ch211-195h23.2	12
si:ch211-212m21.5	12
si:ch211-284o19.6	12
si:ch211-66b9.3	12
si:ch211-93a2.3	12
si:ch73-52e5.2	12
si:dkey-10f21.4	12
si:dkey-208k22.3	12
si:dkey-21k10.1	12
si:dkey-223d7.5	12
si:dkey-52k20.12	12
si:dkey-5i22.6	12
si:rp71-1g18.13	12
slc1a7	12
slc25a24l	12
slc25a36a	12
slc35d1b	12
slc4a2a	12
slc6a9	12
st6galnac3	12
syt11a	12
tfa	12
tfr1a	12
tgfbr1	12
tmem71	12
tmx3	12
tnr	12
tnw	12
tpo	12
trim55a	12
trpa1a	12
twsg1a	12
vmhc	12
wnt3l	12
wu:fc83f10	12
wu:fj23a05	12
wu:fk86g11	12

xirp1	12
zgc:100957	12
zgc:101525	12
zgc:101842	12
zgc:103479	12
zgc:103762	12
zgc:110130	12
zgc:110143	12
zgc:110158	12
zgc:110206	12
zgc:110366	12
zgc:112018	12
zgc:112335	12
zgc:112418	12
zgc:112421	12
zgc:112986	12
zgc:113144	12
zgc:113452	12
zgc:113518	12
zgc:113531	12
zgc:114069	12
zgc:123035	12
zgc:136336	12
zgc:136639	12
zgc:152670	12
zgc:152732	12
zgc:153394	12
zgc:153615	12
zgc:153633	12
zgc:153637	12
zgc:153925	12
zgc:154006	12
zgc:154007	12
zgc:158346	12
zgc:158620	12
zgc:158703	12
zgc:163081	12
zgc:163121	12
zgc:165601	12
zgc:171831	12
zgc:172040	12
zgc:172106	12
zgc:172298	12
zgc:175148	12
zgc:73229	12
zgc:77177	12

zgc:85976	12
zgc:91922_dup1	12
zgc:91922_dup2	12
zgc:92116	12
zgc:92124	12
zgc:92185	12
zgc:92247	12
zgc:92416	12
zgc:92789	12
actc1a	12
ahsa1	12
allc	12
angptl1	12
ankrd6	12
aqp4	12
asap2b	12
atf3	12
atp6v1d	12
bmf2	12
bon	12
ccdc85cb	12
ccnk	12
cdh2	12
chga	12
clic5	12
clock	12
clu	12
cnr1	12
crfb8	12
crsp3	12
cx23	12
cx28.8	12
cx43	12
cyp2j21	12
cyp2j22	12
cyp2j23	12
cyp2j24	12
cyp2j25	12
cyp2j27	12
cyp2j29	12
cyp2v1	12
cyp2v2	12
daam1l	12
disp1	12
dll4	12
dnmt8	12

dpf3	12
epha7	12
esr1	12
esr2a	12
esrrgb	12
fam102bb	12
fbxo16	12
fkbp3	12
flvcr1	12
fmo5	12
fos	12
foxc1b	12
foxo5	12
foxq1	12
fryl_dup1	12
fryl_dup2	12
fynb	12
fzd3	12
galnt14	12
gata4	12
gpr126	12
gpr176	12
grem1a	12
hey2	12
hlx1	12
hsdl1	12
hsp90a.1	12
hsp90a.2	12
id2b	12
ift172	12
igfbp1a	12
igfbp3	12
insm1a	12
ism2	12
itpk1	12
ivns1abpa	12
jun	12
kcnf1	12
kcnh5a	12
kdr	12
kidins220b	12
kita	12
klf7l	12
lama4	12
lin9	12
lnx1	12

LOC100149204	12
LOC100150082	12
LOC555925	12
LOC556137	12
LOC556730	12
LOC556789	12
LOC558616	12
lrp11	12
map3k5	12
matn3a	12
mcm3l	12
mep1a.2	12
moxd1l	12
mpv17	12
mycn	12
nfkbiaa	12
nkx2.1a	12
nkx2.2b	12
nr2e1	12
nt5e	12
numbl	12
ociad2	12
otof	12
pak6	12
papolb	12
paqr8	12
pcmt	12
pdc2	12
pdgfra	12
pex7	12
pnoc	12
prdx6	12
prkch	12
prrx1b	12
ptgs2b	12
ptk2bb	12
rasl11b	12
rd3	12
rgs6	12
rhag	12
rhoj	12
rtn1b	12
runx2b	12
sc:d0374	12
scg5	12
serpinb1	12

serpinb1l2	12
sfrs5b	12
sgcb	12
si:ch211-105n9.1	12
si:ch211-11m18.2	12
si:ch211-121a2.2	12
si:ch211-153j24.4	12
si:ch211-157m21.1	12
si:ch211-184m19.2	12
si:ch211-191a24.4	12
si:ch211-199i15.4	12
si:ch211-199l3.1	12
si:ch211-199m3.8	12
si:ch211-200p13.4	12
si:ch211-20b12.2	12
si:ch211-219i10.1	12
si:ch211-239d11.1	12
si:ch211-241j12.1	12
si:ch211-241p10.1	12
si:ch211-246k22.2	12
si:ch211-255d18.4	12
si:ch211-260g14.8	12
si:ch211-261f7.1	12
si:ch211-261f7.2	12
si:ch211-51n14.2	12
si:ch73-18j6.1	12
si:dkey-119m7.4	12
si:dkey-12h9.13	12
si:dkey-15j16.4	12
si:dkey-174m14.3	12
si:dkey-181i3.3	12
si:dkey-183n20.15	12
si:dkey-185l3.3	12
si:dkey-19f21.3	12
si:dkey-221h15.2	12
si:dkey-233p4.1	12
si:dkey-239i20.2	12
si:dkey-25e12.3	12
si:dkey-260n20.1	12
si:dkey-266k12.1	12
si:dkey-30j22.11	12
si:dkey-33i22.2	12
si:dkey-51e6.1	12
si:dkey-5g7.3	12
si:dkey-60a16.5	12
si:dkey-63j12.2	12

si:dkey-63j12.5	12
si:dkey-77b17.2	12
si:dkey-7c18.24	12
si:dkey-7c18.9	12
si:dkeyp-11g8.6	12
si:dkeyp-59a8.1	12
si:dkeyp-59a8.4	12
si:dkeyp-72h1.2	12
si:dkeyp-8f4.4	12
si:dkeyp-95d10.1	12
si:rp71-81o21.2	12
six4.1	12
six6b	12
slc25a27	12
snap25a	12
sox7	12
spata18	12
srd5a3	12
stx11b.1	12
stx11b.2	12
tagap	12
tatdn3	12
tbc1d7	12
tfap2b	12
tnfrsf21	12
tsta3	12
ust	12
uts1	12
xkr6	12
zfp36l1l	12
zgc:100864	12
zgc:100997	12
zgc:101058	12
zgc:101095	12
zgc:101578	12
zgc:101840	12
zgc:101847	12
zgc:103511	12
zgc:109719	12
zgc:110003	12
zgc:110348	12
zgc:111913	12
zgc:112287	12
zgc:113352	12
zgc:114014	12
zgc:114196	12

zgc:123261	12
zgc:136808	12
zgc:136888	12
zgc:153157	12
zgc:153896	12
zgc:153989	12
zgc:158643	12
zgc:162175	12
zgc:171670_dup1	12
zgc:171670_dup2	12
zgc:172112_dup1	12
zgc:172112_dup2	12
zgc:173683	12
zgc:174972	12
zgc:174985	12
zgc:56288	12
zgc:63497	12
zgc:63602_dup1	12
zgc:63667	12
zgc:64076	12
zgc:77870	12
zgc:85763	12
zgc:86709	12
zgc:92183	12
zgc:92851	12
znf395_dup2	12
zp2	12
zp2.3	12
abhd11	12
actn3a	12
adora2a.2	12
agpat9	12
alg8	12
arl3l1	12
bbs1	12
bhmt	12
birc2	12
btc	12
c1qc	12
cadm1a	12
cbr1l	12
cel.1	12
cfb	12
chl	12
cldn2	12
cldnc	12

cldnh	12
col27a1a	12
crb2	12
crhbp_dup1	12
dacha	12
dub	12
esrra	12
fbln4	12
fibpl	12
fstl4	12
gabra1	12
galnt10	12
gfpt2	12
ghrb	12
gria1b	12
gria4b	12
gsdf	12
gsna	12
gstt1b	12
hif1al2	12
htr1ab	12
iclp2	12
il7r	12
irf11	12
jak2a_dup1	12
lhx2a	12
lhx5	12
LOC100150691	12
LOC553495	12
LOC559127	12
LOC792137	12
msxd	12
myo5b	12
ncam1	12
neu3.1	12
nkx6.1	12
nr5a1b	12
nr6a1b	12
nrg2a	12
nrxn2a	12
olfm1b	12
pdlim4	12
pgam2	12
pitx1	12
plrdgb	12
ppp2r2b	12

prdx5	12
prlra	12
ptprm	12
rbm41	12
rbmx2	12
rgs7bpb	12
rhogb	12
ripply1	12
rtn2b	12
rx3	12
rxraa	12
sc5d	12
sgcd	12
si:ch211-208m1.2	12
si:ch211-257i19.2	12
si:rp71-1h20.5	12
slc13a2	12
slc26a1	12
slc45a2	12
slc8a4a	12
smad7	12
spry4	12
st14b	12
st6gal1	12
st6galnac4	12
stxbp1	12
sumf2	12
tcf7	12
thbs4b	12
tlcd1	12
tmem45b	12
tnfrsfa	12
tnfsf10l3	12
trpc2	12
trpc6	12
ubtd2	12
vtnb	12
wu:fc23f06	12
zbtb16	12
zgc:100921	12
zgc:101024	12
zgc:101111	12
zgc:101774	12
zgc:101809	12
zgc:103645	12
zgc:109947	12

zgc:109965	12
zgc:110116	12
zgc:110222	12
zgc:110688	12
zgc:110782	12
zgc:111958	12
zgc:112359	12
zgc:112437	12
zgc:113343	12
zgc:113348	12
zgc:113362	12
zgc:114104	12
zgc:114195	12
zgc:122979	12
zgc:136354	12
zgc:136851_dup1	12
zgc:136851_dup2	12
zgc:136885	12
zgc:136892	12
zgc:152741	12
zgc:152777	12
zgc:152928	12
zgc:153096	12
zgc:153208	12
zgc:153253	12
zgc:153674	12
zgc:153926	12
zgc:158225	12
zgc:158228	12
zgc:158231	12
zgc:158291	12
zgc:158666	12
zgc:162284	12
zgc:162971	12
zgc:165464	12
zgc:165490	12
zgc:165521	12
zgc:165608	12
zgc:171220	12
zgc:171740	12
zgc:171891	12
zgc:171965	12
zgc:172158	12
zgc:172250	12
zgc:172294	12
zgc:175142	12

zgc:175145	12
zgc:55420	12
zgc:56585	12
zgc:63700	12
zgc:64106	12
zgc:73142_dup1	12
zgc:73142_dup2	12
zgc:77262	12
zgc:77377_dup1	12
zgc:77377_dup2	12
zgc:77495	12
zgc:85869	12
zgc:91821	12
zgc:91960	12
zgc:92275	12
zgc:92761	12
accn2a_dup2	12
adarb1	12
adra2a	12
aes	12
ahr1b	12
ahr2	12
amh	12
ankrd29	12
b3gnt7	12
brunol5	12
cacna1sa	12
capn2b	12
capn2l	12
celsr2	12
cenpp	12
cep70	12
crb1	12
crebbpa	12
crlf1b	12
cxcl12b	12
cyb561d2	12
dnajc19	12
dph5	12
dpp4	12
dusp5	12
fbn2b	12
fkbp8	12
foxq2	12
frem1b	12
glrx2	12

glsl	12
gpd1b	12
hsh2d	12
hspb7	12
im:6805837	12
inadl	12
ing5	12
ing5a	12
irf6	12
kal1b	12
kng1	12
lhx9	12
LOC100003898	12
LOC100004942	12
LOC100005958	12
LOC100006793	12
LOC100189617	12
LOC556597	12
LOC556629	12
LOC559473	12
LOC559969	12
LOC563864	12
LOC565106	12
LOC566050	12
LOC568664	12
LOC570228	12
LOC570443	12
LOC571364	12
LOC792190	12
LOC792509	12
LOC793439	12
LOC796378	12
LOC799142	12
lpl	12
malt1	12
mfap2	12
mfng	12
MGC172218	12
ncf4	12
nfia	12
npb	12
nphs2	12
nr5a2	12
nrd1	12
ogn	12
osbpl1a	12

pdgfaa	12
pdzk1ip1l	12
pvalb7	12
rc3h1	12
rdh5	12
rfxank	12
rgs2	12
rx1	12
s1pr1	12
serpinc1	12
sgsh	12
sh3bp4a	12
si:ch211-140f21.1	12
si:ch211-151i8.2	12
si:ch211-154a22.8	12
si:ch211-197g15.10	12
si:ch211-212d10.2	12
si:ch211-212d10.3	12
si:ch211-222k6.1	12
si:ch211-226h8.4	12
si:ch211-262h13.5	12
si:ch73-138e16.8	12
si:ch73-189n23.1	12
si:dkey-110c1.1	12
si:dkey-110c1.4	12
si:dkey-110c1.7	12
si:dkey-15h8.11	12
si:dkey-15h8.7	12
si:dkey-169i5.4	12
si:dkey-171o17.5	12
si:dkey-172o19.1	12
si:dkey-179j5.5	12
si:dkey-188p4.2	12
si:dkey-19a16.4	12
si:dkey-1b17.2	12
si:dkey-1b17.7	12
si:dkey-205o12.6	12
si:dkey-20i20.2	12
si:dkey-20i20.5	12
si:dkey-21e7.2	12
si:dkey-222p3.1	12
si:dkey-236e20.7	12
si:dkey-253d23.3	12
si:dkey-253d23.4	12
si:dkey-253d23.9	12
si:dkey-42i9.7	12

si:dkey-4c15.14	12
si:dkey-4c23.7	12
si:dkey-72g22.1	12
si:dkey-78l4.14	12
si:dkey-94n12.2	12
si:dkey-94n12.3	12
si:dkeyp-20e4.1	12
si:dkeyp-31e2.1	12
si:dkeyp-34c12.1	12
si:dkeyp-34c12.2	12
si:dkeyp-53d3.3	12
si:dkeyp-53d3.6	12
si:dkeyp-84a8.1	12
si:dkeyp-87e7.3	12
si:dkeyp-98a7.10	12
si:dkeyp-98a7.5	12
si:rp71-1i20.2	12
slc12a10.2	12
slc12a10.3	12
sox2	12
srf	12
stat1a	12
stx6	12
tal1	12
tbc1d20	12
ticam1	12
tmcc2	12
tpm4_dup1	12
tpm4_dup2	12
trh1	12
vasn	12
vcam1	12
wu:fe18c06	12
zbtb37	12
zgc:101049	12
zgc:101130	12
zgc:103502	12
zgc:103672	12
zgc:110821	12
zgc:112279	12
zgc:112958	12
zgc:113220	12
zgc:113298	12
zgc:113317	12
zgc:113324	12
zgc:113336	12

zgc:113418	12
zgc:114076	12
zgc:114143	12
zgc:114170	12
zgc:122977	12
zgc:123008	12
zgc:123116	12
zgc:123349	12
zgc:136839	12
zgc:152969	12
zgc:162255	12
zgc:162358	12
zgc:162724	12
zgc:171318	12
zgc:171500	12
zgc:171601	12
zgc:171679	12
zgc:171686	12
zgc:171762	12
zgc:171887	12
zgc:172133	12
zgc:173480	12
zgc:173486	12
zgc:173693	12
zgc:174224	12
zgc:174563	12
zgc:174564	12
zgc:174712	12
zgc:174919	12
zgc:175175	12
zgc:175284	12
zgc:55605	12
zgc:65857	12
zgc:73223	12
zgc:77082	12
zgc:77816	12
zgc:86754	12
zgc:92511	12
zgc:92747	12
acvrl1	12
ada	12
aldh8a1	12
ap4b1l	12
ard1a	12
arf3a	12
arhgef3l	12

atf7a	12
atp1b2a	12
atp2b3b	12
b4galt5	12
bhlhe23	12
bin2a	12
bmp7b	12
c20orf14_dup1	12
cacnb3a	12
calcoco1	12
casp7	12
cbln4	12
cd63	12
cdc42l2	12
chl1	12
cmyb	12
cyp17a2	12
cyp2aa3v1	12
dhrs3a	12
dnase1l3	12
erbb3b_dup2	12
eya4	12
fam132a	12
gata5	12
gdi1	12
gpr182	12
her12	12
her9	12
hspg2	12
icat	12
idh3g	12
igsf21b	12
irf10	12
kctd6	12
klhl21	12
lama5	12
LOC100149258	12
LOC497072	12
LOC553461	12
mafba	12
mapre1	12
masp2	12
MGC171407	12
mip1	12
mip2	12
mitfb	12

mlck2	12
mxra8b	12
nadl1.1	12
nadl1.2	12
nfe2	12
ntf7	12
oprl	12
pa2g4b_dup1	12
pa2g4b_dup2	12
pard6b	12
pax7b	12
pfkma	12
phlda3	12
plagx	12
pou6f1	12
ptk6b	12
rarga	12
rnd1	12
rnd1l	12
sc:d0205	12
scn8aa	12
sgk2_dup1	12
sgk2_dup2	12
si:dkey-78k11.1	12
si:dkeyp-84g9.1	12
silvb	12
slc2a1	12
slc48a1a	12
snai1b	12
sp5l	12
spo11	12
stat6	12
stat7	12
syn2a	12
tas1r1	12
taz	12
tcf21	12
tfr2	12
thap3	12
tnnt2	12
trappc1	12
tuba1	12
twist3	12
vdra_dup1	12
vdra_dup2	12
wnt10b	12

zgc:100832	12
zgc:100836	12
zgc:101601	12
zgc:101673	12
zgc:101782	12
zgc:103467	12
zgc:103752	12
zgc:103759	12
zgc:109968	12
zgc:111859	12
zgc:112175	12
zgc:112293	12
zgc:112355	12
zgc:114081	12
zgc:114127	12
zgc:114174	12
zgc:123037	12
zgc:123207	12
zgc:123248	12
zgc:136569	12
zgc:136759	12
zgc:136935	12
zgc:152916	12
zgc:153125_dup1	12
zgc:153125_dup2	12
zgc:153284	12
zgc:153597	12
zgc:153722	12
zgc:153779	12
zgc:153784	12
zgc:154035	12
zgc:158254	12
zgc:158263	12
zgc:158791	12
zgc:162289	12
zgc:162431	12
zgc:162502	12
zgc:162623	12
zgc:162858	12
zgc:162889	12
zgc:165605	12
zgc:171453	12
zgc:171590	12
zgc:171755	12
zgc:171775	12
zgc:171837	12

zgc:171857	12
zgc:171945	12
zgc:171947	12
zgc:172103	12
zgc:172129	12
zgc:172315	12
zgc:173570	12
zgc:173927	12
zgc:175128	12
zgc:175180	12
zgc:175287_dup2	12
zgc:193598	12
zgc:194189	12
zgc:194283	12
zgc:194314	12
zgc:195056	12
zgc:63546	12
zgc:63553	12
zgc:65788	12
zgc:66097	12
zgc:73155	12
zgc:73350	12
zgc:73355	12
zgc:77123	12
zgc:77714	12
zgc:85858	12
zgc:85866	12
zgc:86755	12
zgc:86915	12
zgc:91852	12
zgc:91895	12
zgc:91986	12
zgc:92085	12
zgc:92113	12
zgc:92355	12
abi1	12
acaa1	12
acad11	12
acp5b	12
ahrra	12
alg14	12
apbb1ip	12
apoob	12
arx	12
atoh2a	12
bcl2	12

bmp6	12
cebpd	12
cldn11	12
cluap1	12
crh	12
crygn2	12
drd3_dup2	12
dtna	12
ek1	12
elovl2	12
eya1	12
fbxo45	12
flj11011l	12
fzd8a	12
gad2	12
gbx1	12
gcm2	12
gdap1	12
gli3	12
gygl	12
hgd	12
hhatlb	12
hnf4g	12
im:7158043	12
itgb1a	12
lama1	12
lnx2a	12
LOC100141478	12
LOC100141479	12
LOC553509	12
LOC556621	12
LOC792613	12
lrrc33	12
lrrc67	12
lyrm1	12
mbtps2	12
metrn	12
myca	12
myo3a	12
napgl	12
nog2	12
nrp1a	12
olfm3	12
otpa	12
pcolce2b	12
pcyt1ba	12

pdx1	12
pi15a	12
plcd1a	12
prdm14	12
psme1	12
ptgds	12
ptrf	12
rnpc3	12
sat1	12
si:ch211-153c20.4	12
si:ch211-234p6.12	12
si:rp71-1f1.7	12
smpx	12
smyhc1	12
smyhc2_dup2	12
tnfsf10l2	12
trim55b	12
trpa1b	12
vipr2	12
wu:fi12b10	12
zdhhc23	12
zfand1	12
zgc:101053	12
zgc:101685	12
zgc:103519	12
zgc:110069	12
zgc:110761	12
zgc:110852	12
zgc:111976	12
zgc:112054	12
zgc:112185	12
zgc:112214	12
zgc:112332	12
zgc:112496	12
zgc:112515	12
zgc:113385	12
zgc:113390	12
zgc:114120	12
zgc:123262	12
zgc:123339	12
zgc:136586	12
zgc:152652	12
zgc:153038	12
zgc:153154	12
zgc:153503	12
zgc:153639	12

zgc:153824	12
zgc:154125	12
zgc:158236	12
zgc:158335	12
zgc:158623_dup2	12
zgc:158823	12
zgc:158872	12
zgc:162895	12
zgc:163053	12
zgc:165526	12
zgc:171516	12
zgc:171629	12
zgc:171660	12
zgc:171674	12
zgc:171788	12
zgc:172142	12
zgc:173439	12
zgc:173714	12
zgc:174877	12
zgc:193698	12
zgc:194621	12
zgc:194780	12
zgc:64095	12
zgc:64119	12
zgc:64177	12
zgc:65861	12
zgc:77708	12
zgc:77867	12
zgc:91823	12
zgc:91999	12
zgc:92004_dup1	12
zgc:92004_dup2	12
zgc:92111	12
zgc:92218	12
zgc:92631	12
zic1	12
zic4	12
adal	12
adam10b	12
adat1	12
anxa2a	12
atp2b1b	12
bbs4	12
bpgm	12
camk1d	12
cav1	12

cav2	12
ccdc113	12
cib2	12
ckmt1	12
cntn1a_dup1	12
cntn1a_dup2	12
cyb5r2	12
cyp19a1b	12
dhtkd1	12
dkk3	12
e2f4	12
fa2h	12
fads2	12
fanca	12
fbxl22	12
fezf1	12
fgf6a	12
foxb1.2	12
furinb	12
glcea	12
hic1l	12
hsd17b2	12
igf2b	12
irak4_dup1	12
irak4_dup2	12
irf7	12
isl2a	12
islr2	12
kcnj11	12
kcnj11l	12
kctd15l	12
klf13l	12
klhdc4	12
kras	12
lamb1	12
LOC100190888	12
LOC567595	12
LOC797250	12
met	12
mfge8	12
mxg	12
myod1	12
ndrg4	12
ndufa9	12
nfat5	12
pax6a	12

pkm2b	12
pparab	12
pthrp2	12
rag1	12
rag2	12
rhcg	12
rlbp1b	12
roraa	12
sc:d148	12
scaper	12
setd6	12
si:ch211-51l3.1	12
slc13a1	12
slc17a6l	12
slc1a2	12
slc35b4	12
snrk1	12
snx33	12
spon1b	12
sqrdl	12
sycp3l	12
syt9b	12
tdg	12
tigara	12
tnni2a.1	12
tnni2a.2	12
tnnt3a	12
tph1a	12
ush1c	12
wasla	12
wwox_dup2	12
zgc:101757	12
zgc:103506	12
zgc:103631	12
zgc:109782	12
zgc:110459	12
zgc:110749	12
zgc:110773	12
zgc:112242	12
zgc:112297	12
zgc:113516	12
zgc:113571	12
zgc:114162	12
zgc:136338	12
zgc:136656	12
zgc:136771	12

zgc:136844	12
zgc:136967	12
zgc:136980	12
zgc:152941	12
zgc:153678	12
zgc:153718	12
zgc:153923	12
zgc:158222	12
zgc:158626	12
zgc:158678	12
zgc:162127	12
zgc:162267	12
zgc:162329	12
zgc:162618	12
zgc:163141	12
zgc:172145	12
zgc:175154	12
zgc:194153	12
zgc:194665	12
zgc:194786	12
zgc:55574	12
zgc:77060	12
zgc:77593	12
zgc:85947	12
zgc:86895	12
zgc:91896	12
zgc:92245	12
zgc:92294	12
zgc:92481	12
zgc:92489	12
zgc:92873	12
znrf1	12
zte25	12
aanat2	12
abhd3	12
accn1	12
arl16	12
arpc1b	12
asb16	12
asf1b	12
atad4a	12
atp1a3b	12
atp2a1	12
b9d1	12
cacnb1	12
casp6	12

casp6l1	12
cbx4	12
cbx8a	12
cdc42ep4	12
cldni	12
cldnk	12
col1a1	12
coro1a	12
csht12	12
cyp2k6	12
cyp2k8	12
cyp3c1l2	12
dexi	12
epor_dup1	12
eps8l1	12
fahd1	12
fam86a	12
fn3krp	12
foxj1a	12
foxred2	12
ftr49_dup2	12
fzd2	12
gdpd3	12
gfap	12
gh1	12
gna12	12
gpr146	12
grap	12
grna	12
gsg1l	12
hagh	12
hcrt	12
hmox1	12
hoxb10a	12
hoxb13a	12
hoxb2a	12
hoxb7a	12
hs3st3b1a	12
hs3st4	12
hsp70_dup1	12
hsp70_dup2	12
ifn1	12
il21r	12
il2rb	12
itga2b	12
itgb3a	12

junbl	12
kcnh2	12
kctd13	12
lat	12
ldlr	12
lgals1l1	12
lgals1l2	12
lgals3bpa	12
lgals3bpb	12
lin7b	12
litaf	12
LOC100000648	12
LOC100001340	12
LOC100002730	12
LOC100004428	12
LOC100007373	12
LOC100007703	12
LOC100302390	12
LOC559742	12
LOC562205	12
LOC568402	12
LOC571647	12
LOC791519	12
LOC793880	12
LOC797202	12
LOC798075	12
mafg1	12
mafk	12
mgat3	12
mpp2a	12
ndpkz3	12
ndufa4l	12
nfe2l1	12
nme4	12
nog1	12
notch3	12
npas2	12
nr5a5	12
nsf	12
olfm2_dup1	12
olfm2_dup2	12
pdgfab	12
phospho1	12
plbd1	12
plcd3a	12
pmp22a	12

pppde2a	12
prl	12
pvalb5	12
pvalb6	12
pycr1	12
rab11fip4a	12
rac3	12
ramp2	12
rln3	12
rnd2	12
rprml	12
s1pr2	12
sc:d0139	12
sc:d158	12
scn4ab	12
sepw2b	12
si:ch211-284p22.1	12
si:dkey-121j17.2	12
si:dkey-204f11.59	12
si:dkey-56d12.4	12
si:xx-by187g17.8	12
slc17a7	12
slc35b1	12
slc4a1	12
slc5a11	12
smyd2b	12
snn_dup2	12
snx8a	12
socs1	12
socs3a	12
sox8	12
sox9b	12
stat5.1	12
stxbp2	12
syce2	12
syngr1	12
syngr2a	12
syngr3	12
timp2b	12
tpbgl	12
trap1_dup1	12
trap1_dup2	12
ttc25	12
wipi1	12
wnt9b	12
wu:fb01b03	12

wu:fc79b03	12
wu:fj24c01	12
zgc:100838	12
zgc:100868	12
zgc:100935	12
zgc:101633	12
zgc:109934	12
zgc:110152	12
zgc:112038	12
zgc:112091	12
zgc:112146	12
zgc:112484	12
zgc:112492_dup1	12
zgc:113006	12
zgc:113030	12
zgc:113090	12
zgc:113102	12
zgc:113113	12
zgc:113250	12
zgc:113265	12
zgc:113295	12
zgc:113333	12
zgc:113407	12
zgc:114118	12
zgc:123295	12
zgc:123297	12
zgc:136308	12
zgc:136545	12
zgc:136778	12
zgc:136820	12
zgc:136878	12
zgc:152997	12
zgc:153036	12
zgc:153136	12
zgc:153269	12
zgc:153426	12
zgc:153733	12
zgc:153739	12
zgc:153787	12
zgc:153968	12
zgc:154169	12
zgc:158309	12
zgc:158371	12
zgc:158649	12
zgc:158679	12
zgc:158687	12

zgc:162331	12
zgc:162495	12
zgc:162612	12
zgc:162641	12
zgc:162874	12
zgc:162914	12
zgc:163023	12
zgc:165467	12
zgc:165627	12
zgc:171506	12
zgc:171702	12
zgc:172090	12
zgc:172194_dup1	12
zgc:172194_dup2	12
zgc:172253	12
zgc:173517	12
zgc:173573	12
zgc:173575	12
zgc:173577	12
zgc:173593	12
zgc:173615	12
zgc:173617	12
zgc:173638_dup2	12
zgc:174006	12
zgc:174234	12
zgc:174268	12
zgc:174288	12
zgc:174623	12
zgc:174624	12
zgc:174646	12
zgc:175008	12
zgc:193811	12
zgc:194125	12
zgc:194562	12
zgc:194733	12
zgc:195001	12
zgc:195139_dup2	12
zgc:55876	12
zgc:63577	12
zgc:63831	12
zgc:64085	12
zgc:66268	12
zgc:73293_dup1	12
zgc:73293_dup2	12
zgc:73324	12
zgc:77390_dup1	12

zgc:77390_dup2	12
zgc:77407	12
zgc:77415	12
zgc:86896	12
zgc:91874	12
zgc:91984	12
zgc:92162	12
zgc:92296	12
zgc:92419	12
zgc:92870	12
znf750	12
zwi	12
abcc9	12
adm2	12
alg12	12
ankrd16	12
anks1b	12
asb13	12
ascl1a	12
atxn10	12
b2m	12
bcat1	12
bik	12
braf	12
btbd11a	12
cacna1c	12
cacna2d1a	12
ccdc146	12
ccdc3	12
cd36	12
cdc123	12
cdkn1bl	12
chpt1	12
chst11	12
cntn1b	12
cradd	12
cry1a	12
cugbp2	12
dclre1c	12
dcn	12
dpysl5b	12
drd4-rs	12
elk3	12
epyc	12
etv6	12
fam60a	12

fbxl14	12
fgl2	12
fhit	12
foxp2	12
gata3_dup2	12
glt8d3	12
gnai1	12
gnptab	12
gpr19	12
grip1	12
guca1a	12
gys2	12
hgfa	12
itih2	12
kcnd2	12
kiss2	12
lhfpl3	12
lin7a	12
LOC100002785	12
LOC568176	12
LOC568385	12
lrig3	12
lrp6	12
lrrc8d	12
magi2	12
mcat	12
mdm1	12
meig1	12
mest	12
metap2l	12
mgst1	12
mpp1	12
ms4a17a.17	12
msgn1	12
msrb3	12
mybpc1	12
myf6	12
napepld	12
nav3	12
ndufa12	12
net1	12
nots	12
nr2c1	12
opn1sw1	12
optn	12
osgep	12

pah	12
pfkfb3	12
phyh	12
pkp2	12
plxna4	12
pparaa	12
prickle1b	12
prmt8	12
pthlh	12
ptpn12	12
pus7l	12
rassf8	12
recql	12
ripply2	12
sco2	12
sema3aa	12
si:ch211-107n23.1	12
si:ch211-127b11.1	12
si:ch211-132p20.4	12
si:ch211-152c2.1	12
si:ch211-161n3.1	12
si:ch211-198d18.2	12
si:ch211-214j24.7	12
si:ch211-222h17.4	12
si:ch211-234f20.7	12
si:ch211-238e6.5	12
si:ch211-240j22.2	12
si:ch211-244b2.1	12
si:ch211-244b2.3	12
si:ch211-266a5.1	12
si:ch211-51e12.5	12
si:dkey-108d22.2	12
si:dkey-14d8.2	12
si:dkey-14d8.5	12
si:dkey-14d8.6	12
si:dkey-14d8.7	12
si:dkey-14k9.3	12
si:dkey-153k10.9	12
si:dkey-180p18.8	12
si:dkey-207j16.6	12
si:dkey-217k21.2	12
si:dkey-21h14.1	12
si:dkey-21h14.7	12
si:dkey-21o22.1	12
si:dkey-240a12.1	12
si:dkey-240a12.3	12

si:dkey-264k15.2	12
si:dkey-31f5.1	12
si:dkey-31f5.2	12
si:dkey-39a18.1	12
si:dkey-3n7.3	12
si:dkey-61p9.7	12
si:dkey-6a5.3	12
si:dkey-97m3.1	12
si:dkey-9a20.6	12
si:dkeyp-27e10.3	12
si:dkeyp-89c11.2	12
si:dkeyp-89c11.3	12
slc25a3l	12
slc38a4	12
slco1c1	12
socs2	12
sox5	12
sreb2	12
st8sia1	12
syt1	12
tbk1	12
tigarb	12
tmem110l	12
tnnt1	12
trim24	12
vegfab	12
wif1	12
wnt5b	12
wu:fb11h05	12
yaf2	12
zgc:101811	12
zgc:101896	12
zgc:103443	12
zgc:103749	12
zgc:110731	12
zgc:112186	12
zgc:113098	12
zgc:113381	12
zgc:114066_dup1	12
zgc:114066_dup2	12
zgc:136804	12
zgc:136889	12
zgc:153746	12
zgc:153948	12
zgc:158179_dup1	12
zgc:158179_dup2	12

zgc:158374	12
zgc:162209	12
zgc:162958	12
zgc:163027	12
zgc:171551	12
zgc:171553	12
zgc:171604	12
zgc:171727	12
zgc:172139	12
zgc:173624	12
zgc:173706	12
zgc:173708	12
zgc:173713	12
zgc:173720	12
zgc:174273	12
zgc:174315	12
zgc:174700	12
zgc:174708	12
zgc:175035_dup1	12
zgc:175107	12
zgc:194186	12
zgc:194215	12
zgc:194223	12
zgc:194336	12
zgc:55343	12
zgc:55888	12
zgc:56041	12
zgc:73347	12
zgc:76871	12
zgc:77033	12
zgc:77247	12
zgc:77740	12
zgc:85676	12
zgc:85944	12
zgc:85975	12
zgc:86661	12
zgc:86905	12
zgc:91880	12
zgc:91952	12
zgc:92022	12
zgc:92266	12
zgc:92357	12
zgc:92407	12
zgc:92791	12
zgc:92849	12
adam28	12

adora2b	12
agpat9l	12
ak5l	12
aldh2a	12
alg9	12
alkbh6	12
antxr2a	12
anxa1b	12
anxa1c	12
anxa3a	12
ap3m2	12
apoa1	12
aqp3	12
ar	12
arl15	12
arrdc3	12
asb12a	12
ass1	12
atp1b2b	12
barhl1.2	12
bcdo2l	12
c2cd2l	12
c9	12
camk4	12
camkk1	12
cdh11	12
chrne	12
ckma	12
cmklr1	12
cnot6l	12
crfb12	12
cx27.5	12
cyp1d1	12
dab1	12
dab2ip	12
dao.2	12
dao.3	12
dcc_dup1	12
dcc_dup2	12
dhrs11a	12
dlb	12
dlg4	12
dmrt1	12
dmrt2a	12
dmrt3a	12
dpp7	12

eda	12
efnb1	12
ela3l	12
entpd2a.1	12
fbp1a	12
fgf10b	12
flot2a	12
foxd1l	12
foxn4	12
fras1	12
freqa	12
fsta	12
gas1a	12
gas1b	12
gcnt4	12
gna14	12
gnb2	12
gpr98	12
gria3a	12
grin1b	12
grk1b	12
gtf2h3	12
gtf2h3-2	12
hapln1a	12
her1	12
hexa	12
hmgcra	12
hnrpkl	12
hs3st1l2	12
hspb15	12
hspb8	12
isl1	12
kiss1rb	12
lhx1b	12
lifra	12
lman2la	12
lmx1b.2_dup1	12
lmx1b.2_dup2	12
LOC100000539	12
LOC100000978	12
LOC100001127	12
LOC100001344	12
LOC100002237	12
LOC100004647	12
LOC100004874	12
LOC100005195	12

LOC100148058	12
LOC555941	12
LOC557185	12
LOC558007	12
LOC558957	12
LOC559483	12
LOC559803	12
LOC560145_dup1	12
LOC560145_dup2	12
LOC561091	12
LOC561797	12
LOC561820	12
LOC562066	12
LOC562135	12
LOC562345	12
LOC562468	12
LOC564114	12
LOC564545	12
LOC564882	12
LOC565335	12
LOC565548	12
LOC566022	12
LOC566423	12
LOC566487	12
LOC566600	12
LOC566993	12
LOC567417	12
LOC567472	12
LOC567525	12
LOC567536	12
LOC567599	12
LOC567653	12
LOC568767	12
LOC568862	12
LOC569045_dup2	12
LOC569210	12
LOC569320	12
LOC569444	12
LOC569466	12
LOC569571	12
LOC570108	12
LOC570148	12
LOC793774	12
LOC794173	12
LOC794865	12
LOC796505	12

LOC797046	12
LOC797317	12
loxl2b	12
lrrn1	12
man2a1	12
map1b	12
mbd2_dup2	12
mboat4	12
me2	12
mef2cb	12
mpdu1a	12
mthfd2	12
mtx3	12
naaladl1	12
ncf1	12
nelf	12
nipsnap1	12
nos1	12
nos2a	12
notch1b	12
npr3	12
nrarpb	12
og9x	12
olfm1a	12
ophn1	12
ostf1_dup1	12
ostf1_dup2	12
otpb	12
p2rx1	12
p2rx5	12
par1	12
pdlim5	12
pik3ip1	12
pisd	12
plp1b	12
prdm8b	12
prkg2	12
prlrb	12
ptger4b	12
ptges	12
ptgs1	12
rab14l	12
rasgrf2	12
rgmb	12
rhobtb2a	12
rtn4r	12

rufy3	12
rxrab	12
sc:d411	12
scn4bb	12
sdhdb	12
sh2d3cb	12
si:ch211-102c2.4	12
si:ch211-102c2.6	12
si:ch211-117m20.9	12
si:ch211-130m23.3	12
si:ch211-139a5.3	12
si:ch211-157p10.1	12
si:ch211-157p22.10	12
si:ch211-225b11.1	12
si:ch211-236k19.4	12
si:ch211-255a21.1	12
si:ch211-62b4.3	12
si:ch211-89f7.1	12
si:ch211-89p1.1	12
si:dkey-13n15.5	12
si:dkey-174n20.1	12
si:dkey-175c11.1	12
si:dkey-189p24.5	12
si:dkey-193c22.1	12
si:dkey-202n14.1	12
si:dkey-204l11.1	12
si:dkey-267i17.5	12
si:dkey-46g23.3	12
si:dkey-48f17.1	12
si:dkey-71l1.1	12
si:dkeyp-36f5.3	12
si:dkeyp-38h2.4	12
si:dkeyp-7c9.1	12
si:dkeyp-86g2.2	12
si:rp71-10d23.3	12
si:rp71-14i8.2	12
sik2a	12
slc1a3	12
slc30a5	12
slc4a4	12
slc8a2a	12
snx19a	12
spaw	12
spef2	12
spns2	12
spns3	12

spra	12
surf1	12
tagln2	12
tbx2a	12
tbx6	12
tchp	12
thbs4a	12
thnsl2	12
tie2	12
tmem150	12
tnc	12
upk3l	12
vps33a	12
wu:fj48e07	12
zgc:100930	12
zgc:100933	12
zgc:101606	12
zgc:101780	12
zgc:101858	12
zgc:103510	12
zgc:103780	12
zgc:110089	12
zgc:110185	12
zgc:110283	12
zgc:110380	12
zgc:110626	12
zgc:110645	12
zgc:111974	12
zgc:112030	12
zgc:112042	12
zgc:112060	12
zgc:112282	12
zgc:112966	12
zgc:112970	12
zgc:113196	12
zgc:113208	12
zgc:113389	12
zgc:113436	12
zgc:113442	12
zgc:113858	12
zgc:114142	12
zgc:123314	12
zgc:136377	12
zgc:136538_dup1	12
zgc:136538_dup2	12
zgc:136597	12

zgc:136632	12
zgc:152897	12
zgc:153019	12
zgc:153044	12
zgc:153059	12
zgc:153346	12
zgc:153381	12
zgc:153539	12
zgc:153677	12
zgc:153753	12
zgc:153845	12
zgc:153924	12
zgc:153939	12
zgc:158317	12
zgc:158412	12
zgc:158417	12
zgc:158432	12
zgc:158437	12
zgc:158640	12
zgc:162228	12
zgc:162245	12
zgc:162270	12
zgc:162277	12
zgc:162600	12
zgc:162952	12
zgc:171445	12
zgc:171446	12
zgc:171507_dup1	12
zgc:171507_dup2	12
zgc:171581	12
zgc:171734	12
zgc:172187	12
zgc:172206	12
zgc:173737	12
zgc:173738	12
zgc:174650	12
zgc:175187	12
zgc:194112	12
zgc:194158	12
zgc:194812	12
zgc:63972	12
zgc:66350	12
zgc:73226	12
zgc:73336	12
zgc:77336	12
zgc:77869	12

zgc:77891	12
zgc:86927	12
zgc:91813	12
zgc:91957_dup1	12
zgc:91957_dup2	12
zgc:92052	12
zgc:92074	12
zgc:92445	12
zgc:92773	12
znf703	12
aanat1	12
abcc4	12
accn4b	12
adipoql	12
arf3b	12
atg4c	12
barhl2	12
bcl6	12
bmpr2a	12
boka	12
cacnb4b	12
caskb	12
caspxa	12
ccdc65	12
ccdc85al	12
chrna1	12
cldn10	12
crbn	12
cryba2a	12
cth	12
cthl	12
cxcr7b	12
cyp24a1l	12
cyp27c1	12
dmrt2b	12
dytn	12
dzip1	12
erbb3a	12
fkbp11	12
fkbp5	12
foxd3	12
foxp1b	12
fzd7b	12
gad1	12
gata2b	12
gbx2	12

glt8d4	12
gnat1	12
gpd2	12
gpr27	12
h1fx	12
hsd17b7	12
igfbp5b	12
ihhb	12
il12rb2	12
il23r	12
klfd	12
lepr	12
LOC100002901	12
LOC100006122	12
LOC563983	12
LOC566491	12
LOC570897	12
LOC796577	12
LOC798574	12
LOC799271	12
lpp	12
lrrc15	12
mafl	12
mif4gda	12
mlphb	12
myh11	12
myl9	12
ndpkz4	12
nop58	12
nr4a2b	12
ntn1a	12
nudt15	12
opa1	12
parp3	12
pck1	12
pfkmb	12
pglyrp2	12
pink1	12
pou4f1	12
ppp4r2b_dup1	12
ppp4r2b_dup2	12
prkag1	12
ptgisl	12
ptmaa	12
quo	12
rab11fip4b	12

rab6b	12
raly	12
rbl1	12
rbm19	12
rgs4	12
rgs5	12
rhbdl3	12
rhcg2a	12
rhcgl1	12
rhot1b	12
rprm	12
rtk6	12
rybpb	12
scinla	12
scn1lab	12
scn8ab	12
sema3fa	12
sept9b	12
shq1	12
si:ch211-10e8.4	12
si:dkey-10p5.7	12
si:dkey-91f15.1	12
si:dkey-98p3.7	12
slc15a1	12
slc5a8l	12
slc6a11	12
slc6a13l	12
slco2a1	12
sox14	12
sp7	12
speg	12
sumo1	12
sycp1	12
tfap2c	12
tgm2b_dup2	12
tp63	12
trnt1	12
tshb	12
ttc4	12
uhmk1	12
wdr16	12
wdr78	12
wnt2b	12
wu:fc61g08	12
zgc:100903	12
zgc:100915	12

zgc:101035	12
zgc:101788	12
zgc:101792	12
zgc:103466	12
zgc:103539	12
zgc:103559	12
zgc:110586	12
zgc:110647	12
zgc:112163	12
zgc:112416	12
zgc:136465	12
zgc:136817	12
zgc:136840	12
zgc:152917	12
zgc:153063	12
zgc:153083	12
zgc:153139	12
zgc:153311	12
zgc:153573	12
zgc:153648	12
zgc:153702	12
zgc:154009	12
zgc:154085	12
zgc:158252	12
zgc:158700	12
zgc:158731	12
zgc:158737	12
zgc:158824	12
zgc:158846	12
zgc:162180	12
zgc:162630	12
zgc:165344_dup1	12
zgc:165617	12
zgc:165651	12
zgc:171514	12
zgc:171603	12
zgc:171930	12
zgc:172082	12
zgc:172121	12
zgc:172271	12
zgc:174863	12
zgc:175108	12
zgc:194319	12
zgc:194469	12
zgc:55773	12
zgc:56005	12

zgc:56685	12
zgc:63850	12
zgc:73056	12
zgc:92000	12
zgc:92046_dup1	12
zgc:92046_dup2	12
zgc:92105	12
zgc:92231	12
zgc:92731	12
zgc:92836	12
5-ht2cr	12
ache	12
acox3	12
actn3b	12
adcyap1a	12
aldh1a2	12
aldh1a3	12
alox12	12
ank2	12
ankrd46	12
anxa2b	12
arntl1b	12
arrb2b	12
ascl1b	12
bbox1	12
bdnf	12
brms1	12
c1qtnf4	12
c6ast3	12
cacnb2a	12
ccdc123	12
cd81	12
cd99l2	12
cdh5	12
cebpa	12
chchd7	12
cldn15l	12
cmtm3	12
cmtm4	12
col4a5	12
coro1b	12
crabp1b	12
cyb5b	12
cyb5d1	12
dbx1a	12
dtx1	12

efnb3	12
epo	12
f13a1	12
f2	12
fah	12
fbxl8	12
fgf11	12
fgf19	12
fgf3	12
fgf4	12
foxb1.1	12
fshb	12
gc2	12
gnao1b	12
gnrhr2	12
her8a	12
hrh3	12
hsd11b2	12
hspa12b	12
htr5al	12
igf2a	12
insig1	12
irx3a	12
isl2b	12
kcnq1	12
kirrel	12
lingo1b	12
lmo1	12
LOC407719	12
LOC557722	12
LOC558088	12
LOC559167	12
LOC565419	12
LOC565422	12
LOC792476	12
LOC794943	12
lrrc50	12
lypla1	12
map4k2l	12
mdka	12
mespa	12
mespb	12
mmp14a	12
mmp2	12
mnx1	12
mpdu1b	12

mtmr1a	12
mybpc3	12
mylk3	12
nitr3a_dup2	12
nitr3d	12
nitr6a	12
nkd1	12
nom1	12
np	12
npsn	12
npsnl	12
nrxn2b_dup1	12
nrxn2b_dup2	12
nucb2a	12
oaz2l	12
oraov1	12
paqr5b	12
pax6b	12
pc	12
pde5a	12
per1b	12
plag1	12
plagl2	12
poln	12
ppp1r14b	12
prkri	12
pth2	12
ptprja	12
pygmb	12
rab39b	12
rab8b	12
rnf32	12
rorab	12
rrad	12
sall1a	12
scube2_dup1	12
sdr16c5	12
si:ch211-138a11.5	12
six7	12
slc22a18	12
slc3a2	12
slc8a4b	12
slc9a5	12
smad3a	12
smad6a	12
smarcd3b	12

snai3	12
sox17	12
sox6	12
st5	12
stk33	12
syt4	12
tbc1d19	12
tbx1	12
tcf12	12
tmem41b	12
tmem88b	12
tnfsf10l	12
tnni2b.1	12
tnni2b.2	12
tph1b	12
trappc2l	12
tspan18a	12
tubgcp4	12
vps33b	12
wu:fi05d05	12
zgc:101715	12
zgc:101810	12
zgc:103586	12
zgc:103619	12
zgc:110008	12
zgc:110179	12
zgc:112165	12
zgc:113025	12
zgc:113057	12
zgc:113305	12
zgc:113355	12
zgc:123255	12
zgc:136220	12
zgc:136763	12
zgc:136869	12
zgc:153043	12
zgc:153148	12
zgc:153151	12
zgc:153219	12
zgc:153723	12
zgc:153790	12
zgc:153941	12
zgc:153993	12
zgc:154027	12
zgc:158420	12
zgc:158463	12

zgc:158632	12
zgc:158677	12
zgc:158742	12
zgc:162140	12
zgc:162171	12
zgc:162217	12
zgc:162297	12
zgc:162648	12
zgc:162904	12
zgc:162945	12
zgc:165452	12
zgc:165481	12
zgc:165489	12
zgc:171281	12
zgc:171489	12
zgc:172079	12
zgc:172137	12
zgc:172244	12
zgc:174688	12
zgc:175099	12
zgc:175137	12
zgc:175177	12
zgc:193500	12
zgc:193684	12
zgc:194225	12
zgc:194563	12
zgc:194678	12
zgc:194930	12
zgc:195027	12
zgc:195240	12
zgc:65987	12
zgc:73292	12
zgc:73349	12
zgc:76883	12
zgc:92192	12
zgc:92429	12
zgc:92649	12
zgc:92732	12
zgc:92903	12
zplxdc2	12
acbd6	12
accn2b	12
acot7	12
adora2aa	12
adrb3a	12
agtpbp1	12

ahcyl1	12
alas2	12
ampd1	12
atoh1a	12
atp2b3a	12
bcar3	12
bgn	12
cabp1	12
cacna1s	12
cbwd	12
col2a1a	12
csf1b	12
cyr61	12
cytsaa	12
dio1	12
dmrta2	12
dusp2	12
efna5b	12
elavl4	12
eps8l3	12
exorh	12
fam101a	12
fam125bb	12
fam160b2	12
fance	12
fem1a	12
fgd	12
fgf17	12
foxe3	12
foxp3	12
frap1	12
freqb	12
fsd1	12
gck	12
ggt1	12
ggt5	12
ghra	12
glt25d2	12
gnai3	12
gpd1a	12
gpr133	12
gpr173	12
grid2	12
gstm3	12
her3	12
hnf1a	12

hsd17b3	12
hsd17b4	12
hsp70l	12
htr1aa	12
ift81	12
igf3	12
ikbkb	12
itgb4	12
itpr3	12
kazald3	12
kcnd3	12
kcnn1	12
lgi3	12
lhx4	12
lmx1b.1	12
LOC100000333	12
LOC100001681	12
LOC100002223	12
LOC100002795	12
LOC100002993	12
LOC100003386	12
LOC100003662	12
LOC100004076	12
LOC100004270	12
LOC100004557	12
LOC100006238	12
LOC100006275	12
LOC100149189	12
LOC100150997	12
LOC556480	12
LOC557793	12
LOC558400	12
LOC560078	12
LOC560226	12
LOC560297	12
LOC560966	12
LOC561637	12
LOC562144	12
LOC563309	12
LOC564121	12
LOC564423	12
LOC564675	12
LOC564734	12
LOC564840	12
LOC565282	12
LOC565874	12

LOC567059	12
LOC567061	12
LOC567064	12
LOC567726	12
LOC567953	12
LOC568003	12
LOC568129	12
LOC570672	12
LOC792835	12
LOC797209	12
LOC798846	12
LOC799483	12
map3k7ip3l	12
mapkapk2b	12
mecp2	12
mpnd	12
msi1	12
nefl	12
nefm	12
nexn	12
nkx2.7	12
notch2	12
nr2c2	12
nr5a1a	12
nudt18	12
p2rx4b	12
p2rx7	12
pbp	12
pbx3b	12
plcxd3	12
plk2	12
plod1a	12
plp2	12
ppardb	12
ppdpfa	12
pptc7	12
praf2	12
prkcz	12
prr16	12
ptger4a	12
rab3c	12
ralgps1	12
renbp	12
rgs7bpa	12
rpepb	12
sema3ga	12

sema3gb	12
sema3h	12
sepp1a	12
si:ch211-106g4.1	12
si:ch211-156j16.1	12
si:ch211-229n2.7	12
si:ch211-232d19.4	12
si:ch211-251b21.1	12
si:ch211-251j10.4	12
si:dkey-16k6.2	12
si:dkey-202c5.1	12
si:dkey-24f17.5	12
si:dkey-6n6.1	12
si:dkey-6n6.2	12
si:dkey-75a21.2	12
si:dkeyp-110g5.2	12
si:dkeyp-22b2.3	12
si:dkeyp-38g6.2	12
si:dkeyp-38g6.3	12
skia	12
slc12a10.1	12
slc20a1a	12
slc38a3	12
slc5a5	12
slc5a9	12
smyd1b	12
sort1b	12
spata6	12
st6galnac5	12
sult1st1	12
sult1st3	12
sypa	12
sypb	12
sypl2b	12
tas1r2.2	12
tas2r202	12
tfe3a	12
tp73	12
traf1	12
uckl1	12
unc45b	12
upb1	12
wasb	12
wnt2bb	12
wnt5a	12
wu:fa96d09	12

zgc:100920	12
zgc:101100	12
zgc:101530	12
zgc:101844	12
zgc:103657	12
zgc:110231	12
zgc:110299	12
zgc:110353	12
zgc:110354	12
zgc:110383	12
zgc:110609	12
zgc:110694	12
zgc:112361	12
zgc:112522	12
zgc:113060	12
zgc:113105	12
zgc:113115	12
zgc:113363	12
zgc:123115	12
zgc:123269	12
zgc:152691	12
zgc:153440	12
zgc:153507	12
zgc:153516	12
zgc:153628	12
zgc:153631_dup1	12
zgc:153631_dup2	12
zgc:153738	12
zgc:153763	12
zgc:154046	12
zgc:158390	12
zgc:158569	12
zgc:158613	12
zgc:158858	12
zgc:158862	12
zgc:162251_dup2	12
zgc:162337	12
zgc:171501	12
zgc:171538	12
zgc:171872	12
zgc:172102	12
zgc:172155	12
zgc:173710	12
zgc:173711	12
zgc:173994	12
zgc:174931	12

zgc:194839	12
zgc:194990	12
zgc:55392	12
zgc:55889	12
zgc:63721	12
zgc:63990	12
zgc:64161	12
zgc:66106	12
zgc:77806	12
zgc:85723	12
zgc:85787_dup1	12
zgc:85787_dup2	12
zgc:86586	12
zgc:91787	12
zgc:91909	12
zgc:92280	12
zgc:92316	12
ankar	12
ankrd10b	12
arl4cb	12
asb1	12
atp1a1b	12
bbs5	12
bmpr2b	12
cacnb4a	12
ccdc80	12
col18a1	12
col28a1a	12
col5a2l	12
col8a1a	12
creg2	12
crfb1	12
crfb2	12
crfb4	12
crfb5	12
cryl1	12
cx33.8	12
cx45.6	12
dcbld2	12
dlx1a	12
efhc2	12
efnb2a	12
egfl6	12
eng1a	12
epb41l5	12
evx2	12

f5	12
fev	12
fgf14	12
fign	12
frzb	12
ftr53	12
glb1l	12
gli2a	12
gpm6bb	12
gpr183	12
grtp1b	12
gsk3b	12
hao2	12
heg	12
hlxb9la	12
hnrnpa3	12
hoxd11a	12
hoxd12a	12
hoxd13a	12
hoxd9a	12
hpx	12
hs6st3	12
hspbap1	12
ift88	12
igf2bp2a	12
igfbp5a	12
igsf3	12
ikzf2	12
il1rapl1a	12
ildr2	12
im:7152538	12
itga6	12
itgbl1	12
itm2bb	12
jagn1a	12
kctd12.1	12
klf12b	12
lats2	12
lect1	12
lmo7a	12
LOC100003476	12
LOC100004456	12
LOC100004613	12
LOC100149419	12
LOC100192381	12
LOC554606	12

LOC558828	12
LOC561317	12
LOC564685	12
LOC565294	12
LOC565563	12
LOC565876	12
LOC569399	12
LOC792815	12
lss	12
lypd6	12
mao	12
map3k12	12
mettl8	12
mstn	12
mxe	12
mylk	12
nalcn	12
ncam2	12
nfe2l2	12
nhlh2	12
nostrin	12
nr1i2	12
nrp2b	12
ofd1	12
olig2	12
osbpl6	12
osgepl1	12
pcbp3	12
pikfyve	12
pla1a	12
pou12	12
pou2f1b	12
pth2r_dup1	12
pth2r_dup2	12
ptplb	12
ptpn4	12
ptprn	12
rab3gap1_dup1	12
rab3gap1_dup2	12
rab5b	12
rdh1l	12
rnd3b	12
satb2	12
scel	12
scn1a	12
scrn3	12

sec22a	12
si:ch211-125e6.14	12
si:ch211-160b11.5	12
si:ch211-194d6.1	12
si:ch211-235e18.3	12
si:ch211-93a19.1	12
si:dkey-11f4.16	12
slc25a6	12
slc40a1	12
sox1a	12
sp5	12
sprx	12
stat1b	12
stat4	12
tas2r201.1	12
tas2r3	12
tbx15	12
tfcp2l1	12
tfpia	12
tmem30c	12
tmem37	12
tnfaip6	12
trappc2	12
tslpr	12
ttc21b	12
tuba8l3	12
ugt1ab	12
vangl1	12
vil1l	12
wnt10a	12
wu:fj33d03	12
xrcc5	12
zeb2a	12
zgc:101559	12
zgc:110028	12
zgc:110187	12
zgc:110625	12
zgc:112257	12
zgc:113334	12
zgc:123096	12
zgc:136439	12
zgc:153268	12
zgc:153521	12
zgc:153901	12
zgc:154110	12
zgc:158316	12

zgc:158869	12
zgc:162126	12
zgc:162396	12
zgc:162471	12
zgc:162707	12
zgc:162780	12
zgc:163002	12
zgc:165571	12
zgc:171497	12
zgc:172182	12
zgc:172268	12
zgc:174680	12
zgc:174697	12
zgc:175274	12
zgc:56376	12
zgc:91849	12
zgc:91870	12
zgc:92380	12
zgc:92428	12
zgc:92566	12
zgc:92723	12
zic2a	12
zic5	12
znf148	12
znf385b_dup1	12
znf385b_dup2	12
znfl1	12
hnrnpa0	13
apoeb	13
zgc:85717	13
akap12	13
hmgiy	13
krt18	13
krt8	13
ddx5	13
krt4	13
zgc:163047	13
pcna	14
ccna2	14
pnrc2	14
rcc1	14
cbx3a	14
eef2l	14
rps8	14
hsp90ab1	14
pou5f1	14

rps14	14
bzw1a	14
hnrpa1	14
zgc:193933	14
zgc:56418	14
zgc:56676	14
prmt1	14
zgc:171753	14
rpl12	14
p4hb	14
eif4a1a	14
si:ch73-237c6.4	14
bzw1b	14
sumo3	14
zgc:110113	14
zgc:85811	14
alkbh5	15
arfip1	15
arhgap10_dup2	15
atic	15
cab39l	15
chic2	15
eed	15
exosc9	15
LOC563777	15
ltv1_dup2	15
melk	15
mtap	15
ndufs8	15
pcm1	15
poll	15
polr1e	15
rbpja	15
setd4	15
suclg1	15
taf5l	15
tdrd7	15
tmem192	15
tmx2	15
tubgcp3	15
zgc:101679	15
zgc:103421	15
zgc:171518	15
zgc:195037	15
zgc:77713	15
zgc:86602	15

zgc:92177	15
c11orf2	15
c13orf22l	15
c21orf59l	15
cetn3_dup2	15
gnsb	15
hmgcs1	15
ndufa8	15
nudt2	15
picalm	15
ppapdc1b	15
rab6a	15
reep5	15
slc25a1	15
zcchc9	15
zgc:110193	15
zgc:114200	15
zgc:123177	15
zgc:153126	15
zgc:158685	15
zgc:171444	15
zgc:55536	15
zgc:56259	15
zgc:63505_dup2	15
zgc:73187	15
zgc:92321	15
adipor1a	15
ap1s2	15
arpc4l	15
cxxc1	15
dia1	15
ect2	15
ipo9	15
kdm5ba	15
mcrs1	15
mdm4	15
mfsd4	15
mknk1	15
mkrn2	15
nfs1	15
park7	15
timm17a	15
traip	15
zgc:100849	15
zgc:123326	15
zgc:136409	15

zgc:154067	15
zgc:173544	15
zgc:173548	15
zgc:56708	15
zgc:63992	15
zgc:66391	15
zgc:73328	15
zgc:86903	15
arhgef7b	15
arl4d	15
atpaf2	15
cops3	15
fkbp10	15
im:7155161	15
polr3e	15
prdm9	15
ptenb	15
svil	15
tomm22	15
zgc:101654	15
zgc:112178	15
zgc:112184	15
zgc:112403	15
zgc:123307	15
zgc:152954	15
zgc:175133	15
zgc:194486	15
bag5	15
eif2ak3	15
golga5	15
hells	15
hif1an	15
inpp5f	15
LOC799312	15
lrrfip2	15
memo1	15
ppa1	15
prdx3	15
pygl	15
rufy2	15
si:ch211-67n3.7	15
snx5	15
synj2bp	15
tbp	15
tmx1	15
zgc:110677	15

zgc:110727	15
zgc:153607	15
zgc:153613	15
zgc:158660	15
zgc:162162	15
zgc:63904	15
zgc:66299	15
hdac3	15
ids	15
ndfip1	15
pcgf1	15
prelid1	15
psmd10	15
ptcd3	15
rnf103	15
rps6kal	15
rraga	15
ssrp1a	15
ubl3	15
wnt8a	15
zdhhc24	15
zgc:110741	15
zgc:113346	15
zgc:113425	15
zgc:153420	15
zgc:158245	15
zgc:171797	15
zgc:193699	15
zgc:193711	15
zgc:66282	15
brca2	15
calm3a	15
gosr1	15
mos	15
mrpl44	15
poldip2	15
sdhda	15
tmprss4a	15
wsb1	15
zgc:152863	15
zgc:153368	15
zgc:162339	15
zgc:163014	15
zgc:55363	15
zgc:56547	15
zgc:66030	15

zgc:73340	15
adnp2	15
eaf1	15
glipr2l	15
grwd1	15
gsk3a	15
josd2	15
kiaa0947l	15
m6pr	15
mios	15
plekhf2	15
rab13	15
scnm1	15
sfrs18	15
tmem147	15
trmt11	15
wu:fc17a11	15
zdhhc18	15
zgc:110182	15
zgc:114128	15
zgc:123238	15
zgc:153260	15
zgc:162590	15
zgc:66432	15
zgc:92862	15
adob	15
entpd6	15
galcb	15
gpr137ba	15
heatr5a	15
mrp63	15
p4ha1	15
plekha1	15
pole2	15
ppp1cb	15
rtkn2	15
slc39a9	15
smyd2a	15
sptlc2_dup1	15
stx11a	15
zbtb2a	15
zfp36l2	15
zgc:122989	15
zgc:158138	15
zgc:162164	15
zgc:171819	15

zgc:92212	15
arntl2	15
blvrb	15
cd151l_dup2	15
chkb	15
cirh1a	15
cry1b	15
fam107b	15
got2a	15
icln	15
LOC557125	15
mbtps1	15
n4bp1	15
pdcd10a	15
prpf18	15
ranbp10	15
si:ch211-201b11.2	15
siah1	15
usp14	15
utp15	15
zgc:103540	15
zgc:110109	15
zgc:194766	15
zgc:66441	15
znf143	15
ak2	15
ccdc106	15
ctdp1	15
dazl	15
eomesa	15
ints3	15
LOC799177	15
lpcat1	15
mrpl3	15
mrps18b	15
mycl1b	15
ndufs5	15
pygo2	15
sdha	15
serinc2	15
sesn2	15
setdb1a	15
si:ch211-129n15.3	15
si:ch211-215a10.4	15
si:dkey-204a24.7	15
si:dkey-261i16.3	15

stx12	15
tmem57a	15
zgc:101026	15
zgc:110753	15
znrd1	15
alg3	15
ankrd12	15
ap2m1a	15
bokb	15
cnpy1	15
crsp7	15
ctdsplb	15
f3b	15
ftr67	15
hig1	15
lhx8	15
mtf2	15
paxip1	15
rab8a	15
rbp1b	15
sdhc	15
sh3gl1a	15
si:ch211-105d4.5	15
si:ch211-61f14.1	15
stam	15
stk25	15
wdr48	15
yipf1	15
zgc:101602	15
zgc:101676	15
zgc:101761	15
zgc:154070	15
zgc:154086	15
zgc:154091	15
zgc:158349	15
zgc:158450	15
zgc:163067	15
zgc:163093	15
zgc:173556	15
zgc:55781	15
zgc:66414_dup1	15
zgc:92204	15
zgc:92437	15
zp3.2	15
acbd3	15
cox7a2	15

dap1b_dup1	15
eif2s1	15
gorab	15
laptm4a	15
lsm4	15
mrpl19	15
nme2l	15
nup43	15
ppp1r13b	15
rad51	15
rnaseh1	15
si:ch211-150c22.3	15
si:ch211-195d17.2	15
si:ch211-241j12.3	15
si:ch211-57i17.1	15
si:dkey-12h9.7	15
tdh	15
ubr7	15
zar1	15
zbtb1	15
zgc:110816	15
zgc:77655	15
dnajc18	15
f2rl1	15
fam175a	15
frag1	15
glrx	15
hspa4l_dup1	15
pias4l	15
ppme1	15
pus1	15
rnf180	15
sdad1	15
vps29	15
zgc:101072	15
zgc:101080	15
zgc:101748	15
zgc:158319	15
zgc:171911	15
zgc:171957	15
zgc:193801	15
zgc:194876	15
zgc:66125	15
zgc:86647	15
atad3b	15
b3galt2	15

bysl	15
ccdc76	15
enc1	15
mxi1	15
polr3f	15
shd	15
si:ch211-129c21.1	15
si:dkey-182g1.2	15
stil	15
tdrd5	15
tm2d1	15
tmem59l	15
zgc:103496	15
zgc:152965	15
zgc:153675	15
zgc:194551	15
zgc:56402	15
zgc:77285	15
zgc:91918	15
asb8	15
c20orf20	15
dip2b	15
dnmt5	15
eif4e3	15
graspl	15
ikbkg	15
lmnl3	15
LOC100007669	15
plod3	15
ptges3	15
rbm38	15
rybpa	15
sgk1	15
slc25a33	15
trpc4apb	15
zgc:101663	15
zgc:112069	15
zgc:171708	15
zgc:174896	15
zgc:174906	15
zgc:56200	15
zgc:66440	15
zgc:91802	15
chmp4c	15
cry-dash	15
qtrtd1	15

si:ch211-234p6.13	15
tfr1b	15
zgc:154015	15
zgc:77318	15
arpp19b	15
det1	15
gpia	15
idh3a	15
lactb	15
snx1	15
tpm1	15
zgc:100789	15
zgc:111826	15
zgc:113217	15
zgc:153606	15
zgc:162740	15
zgc:56361	15
znf277	15
adsl	15
alg1	15
atp6v0c	15
ccdc43	15
clpp	15
cog7	15
drg2	15
dus1l	15
gps1	15
grb2	15
mrps7	15
pdxdc1	15
ppan	15
prkcsh	15
prpsap2	15
qtrt1	15
rarab	15
slc27a1	15
smcr7l	15
spop	15
st13	15
suv420h2	15
traf7	15
ube2i2	15
wipi2	15
wwp1	15
xpo6	15
zgc:110046	15

zgc:110417	15
zgc:112372_dup1	15
zgc:112372_dup2	15
zgc:114109	15
zgc:56457	15
zgc:66419	15
arfgap3	15
fam116b	15
klhdc10	15
mcm10	15
si:ch211-117l16.3	15
si:dkey-159a18.3	15
si:dkey-180p18.2	15
stk38l	15
tnpo3	15
zgc:152938	15
zgc:154045_dup1	15
zgc:195152	15
zgc:73329	15
znf326	15
ankle2	15
arrdc1a	15
atp5l	15
atp6v1g1	15
ccnh	15
exosc2	15
fancg	15
fbxw2	15
gpr107_dup1	15
gpr107_dup2	15
gps2	15
hinfp	15
lman2lb	15
LOC562372	15
LOC569036	15
lsmd1	15
mccc2	15
mobkl1b	15
paip1	15
paqr3a	15
pgam5	15
phf23a	15
ppil2	15
prkaa1	15
sdf2l1	15
senp3a	15

serinc5	15
si:ch211-102c2.7	15
si:ch211-106a19.2	15
si:ch211-155m12.3	15
si:ch211-268m12.6	15
si:dkey-189h5.5	15
si:dkey-69h6.7	15
si:dkeyp-20g2.6	15
slc12a9	15
smn1	15
spag7	15
surf4l	15
surf6l	15
tor2a	15
vamp8	15
zfand5a	15
zgc:100937	15
zgc:103518	15
zgc:103692	15
zgc:110436	15
zgc:153352	15
zgc:193538	15
zgc:55347	15
zgc:73351	15
zgc:77488	15
zgc:86811	15
zgc:92201	15
zgc:92586	15
zmat5	15
apg3l	15
c1galt1b	15
cbx8b	15
cx44.2	15
dtymk	15
hars2	15
lrrc40	15
myh9l2	15
ndufb5	15
ndufs1	15
pde6d	15
pfdn5	15
pgm1	15
prkcd	15
sec23b	15
slc48a1b	15
ube3a	15

uqcrc1	15
zgc:103624	15
zgc:112077	15
zgc:152986	15
zgc:162082	15
zgc:162302	15
zgc:163086	15
zgc:171784	15
zgc:55944	15
zgc:85657	15
zgc:91836	15
zgc:92027	15
ap1g1	15
arhgap1	15
asb7	15
atp6v0d1	15
bad	15
bmp15	15
ck2a2a	15
csnk1g1	15
exosc6	15
gro1	15
grpel1	15
mtm1	15
mus81	15
ntn4	15
ogfod1	15
pfdn2	15
ptgr1	15
rbm4.1	15
rcor2	15
rgp1	15
rhpn2	15
serpine1	15
si:busm1-132m23.3	15
tinf2	15
tmem9b	15
tollip	15
tradd	15
ube3c	15
vbp1	15
vps35	15
zgc:110022	15
zgc:110256	15
zgc:153228	15
zgc:158564	15

zgc:165446	15
zgc:55995	15
zgc:56324_dup1	15
zgc:56324_dup2	15
zgc:63526	15
atp2a2a	15
atxn7l2b	15
calrl2	15
ccdc124	15
chmp7	15
ciao1	15
cpox	15
csnk1g2b	15
fxn	15
gle1l	15
gstm	15
impdh2	15
LOC560225	15
mlec	15
ptgesl	15
rhoad	15
si:dkey-39n1.2	15
si:dkey-46a10.3	15
si:dkey-72l14.4	15
spryd4	15
tjp2b	15
tmem115	15
zgc:101661	15
zgc:103536	15
zgc:152859	15
zgc:152900	15
zgc:162200	15
zgc:162244	15
zgc:171480	15
zgc:56596	15
zgc:92666	15
arl5a	15
asmtl	15
cdc16	15
cog3	15
coq10b	15
epc2	15
ildr1	15
im:6907446	15
ints6	15
LOC569865	15

med4	15
mettl5	15
sestd1	15
snx4	15
stk17b	15
xpo4	15
zgc:101814	15
zgc:101851	15
zgc:112056	15
zgc:112089	15
zgc:113169	15
zgc:85857	15
ddx39a	16
hmgb2	16
rps3a	16
npm1	16
ppia	16
zfand5b	16
zgc:153686	16
nhp2l1	16
rac1	16
zgc:64114	16
fbxo5	16
mycl1a	16
g3bp1	16
gnb2l1	16
hnrnpab	16
ran	16
rpl39	16
sfrs1l	16
s100a1	16
dvr1	16
marcks	16
ilf2	16
stmn1a	16
abcf2	16
hnrnpu	16
rps7	16
syncrip	16
atp5a1	16
setb	16
bsg	16
ncl	16
rps12	16
zgc:56699	16
zgc:56095	16

rpl19	16
rpl23	16
rps2	16
tnpo2	16
nono	16
rpl7a	16
xbp1	16
eef1b2	16
hdlbp	16
rpsa	16
snrpb_dup1	16
snrpb_dup2	16
sumo3l	16
zgc:66168	16
ccne	16
rps20	16
magoh	16
snrpd3	16
c2orf24	17
clta	17
LOC559409	17
mettl14	17
narg1b	17
zgc:158305	17
zgc:85910	17
alg5	17
cdc37l1	17
cetn3_dup1	17
hyou1	17
scarb1	17
sigmar1	17
zgc:153345	17
zgc:63505_dup1	17
cdk2	17
cse1l	17
dda1	17
kbtbd8	17
psma5	17
rnu3ip2	17
sh2d5	17
st3gal2l	17
tmem110	17
uba3	17
zgc:162272	17
zgc:171659	17
baiap2l1a	17

josd1	17
kntc2l	17
pgp	17
phf5a	17
reep3	17
timm23	17
top2a	17
uqcrc2	17
vasa	17
zgc:158331	17
zgc:171432	17
atl2	17
btaf1	17
cuedc2	17
cyp17a1	17
dnajb12	17
eif4ebp2	17
mcfd2	17
nusap1	17
pgam1a	17
pppde1	17
rnf145	17
rpia	17
supv3l1	17
vps26a	17
whsc1	17
zgc:101897	17
zgc:153332	17
zgc:55307	17
atl3	17
b4galt7	17
cab39l1	17
cpeb4	17
fam122b	17
fbxw11a	17
fnta	17
gnpda1	17
hprt1	17
immt	17
lnx2b	17
maea	17
mxd3	17
neil3	17
phf6	17
rap2c	17
st3gal5	17

stx5a	17
syvn1	17
tmed9	17
uqcrq	17
zgc:103560	17
zgc:110128	17
zgc:110188	17
zgc:56497	17
arcn1l	17
arl4a	17
atp1b3b	17
eml2	17
mrpl28	17
pafah1b1a	17
si:ch211-137a8.2	17
spsb4b	17
tnfaip1	17
zgc:64185	17
cpvl	17
fbxo32	17
fdps	17
rragc	17
zgc:153684	17
zgc:174908	17
zgc:92794	17
cdc2	17
g2e3	17
gale	17
hmgcl	17
id2a	17
peli2	17
phf17	17
timm9	17
wdhd1	17
zgc:103668	17
zgc:175195	17
zgc:55521	17
zgc:56072	17
zgc:85662	17
znf593	17
ap2m1b	17
arf5_dup1	17
asz1	17
c20orf24	17
calub	17
cd82	17

cenpn	17
cox4nb	17
dcun1d5	17
gins2	17
herpud1	17
rnf7	17
suv420h1	17
zgc:113159	17
zgc:64098	17
zgc:73237	17
zgc:77650	17
cap1	17
ldlrap1a	17
pdik1l	17
ppt1	17
ptp4a3	17
si:ch211-191i18.1	17
si:rp71-45k5.4	17
tpi1a	17
ttrap	17
zgc:55843	17
zgc:77241	17
zgc:92624	17
bmi1b	17
calrl	17
cenpl	17
cmpk	17
copa	17
csnk1g2a	17
dad1	17
dvl3	17
gyg1	17
itm2cb	17
klhl20	17
LOC100007710	17
nanos	17
rab2a	17
si:dkey-216e9.5	17
si:rp71-1g18.1	17
ssx2ip	17
tmub1	17
uck2b	17
zgc:103652	17
zgc:110343_dup1	17
zgc:113293	17
zgc:153402	17

zgc:63617	17
zgc:66427	17
zp3b	17
cpsf3	17
katna1	17
map3k7	17
ppp2r5ea	17
rab32	17
scyl3	17
si:dkey-114g7.4	17
si:dkeyp-117h8.4	17
sod2	17
zgc:101037	17
zgc:77665	17
zgc:92578	17
adssl	17
crkl	17
hspa4l_dup2	17
med31	17
pafah1b1b	17
slc35f2	17
smpd4	17
zgc:101826	17
zgc:162344	17
zgc:55870	17
zgc:66477	17
zgc:66483	17
zgc:86764	17
zw10	17
LOC100301575_du[illegible]	17
ncbp2	17
pdcd4b	17
rab11b	17
sass6	17
zgc:66434	17
cs	17
ddost	17
hsd17b10	17
osbpl2	17
rnf146	17
sars	17
slc17a9b	17
slmap	17
tbpl1	17
zgc:153910	17
zgc:194971	17

zgc:63792	17
zgc:77836	17
zgc:92287	17
acat1	17
armc1	17
b4galt6	17
bmi1	17
eif1axb	17
mastl	17
pdia4	17
slc4a2b	17
zgc:101879	17
zgc:172225	17
zgc:66340	17
zgc:73369	17
arntl1a	17
ckap5	17
cry3	17
eif3j	17
gins3	17
hprt1l	17
LOC445411	17
snupn	17
zgc:110687	17
zgc:171719	17
atp5h	17
axin1	17
cdc27	17
coil	17
csnk1da	17
gfer	17
gosr2	17
gtpbp1	17
hsd3b7	17
jmjd6	17
micall2	17
mrpl12	17
mrps34	17
ndel1b	17
raver1	17
rogdi	17
scpep1	17
sept9a	17
shmt1	17
suz12a	17
zfand2a	17

zgc:171779	17
zgc:63736	17
zgc:66443	17
zgc:85812	17
copg2	17
nrf1	17
snd1	17
ube2nl	17
zgc:136263	17
arcn1	17
atp5i	17
cdk7	17
chek2	17
dab2	17
dgcr8	17
dullard	17
eif4a1b	17
ggnbp2	17
golph3	17
lysmd3	17
mpzl2	17
ppp1r14a	17
rhogc	17
rnf128	17
rps6ka3a	17
si:ch211-124k10.2	17
si:ch73-13b6.3	17
timm8b	17
ung	17
zgc:110608	17
zgc:114041	17
zgc:92808	17
zmynd19	17
ctsz	17
eif4enif1	17
inka1b	17
7-Mar	17
orc1l	17
phf16	17
rad18	17
rplp2	17
stk24	17
wdr12	17
zgc:113070	17
arih1l	17
ca7	17

cebpg	17
cugbp1	17
dnaja3a	17
dullardl	17
dync1li2	17
fam60al_dup1	17
haus6	17
heatr3	17
mtch2	17
nedd8	17
pop4	17
scamp2l	17
slc25a44b	17
tyms	17
zgc:103558	17
zgc:110584	17
zgc:110674	17
zgc:153225	17
zgc:162592	17
zorba	17
cdc45l	17
depdc1a	17
LOC567309	17
seph	17
si:ch211-147a11.3	17
smarcb1a	17
tmco1	17
trim36	17
tuba8l	17
zgc:101136	17
zgc:113183	17
zgc:162025	17
zgc:162295	17
zgc:56280	17
zgc:92202	17
ddx3	17
ddx41	17
dnajb11	17
hat1	17
hspe1	17
idh1	17
itgb5	17
lnpa	17
me3	17
si:ch211-67e16.9	17
tfdp1l	17

uchl3	17
zgc:152882	17
zgc:162944	17
zgc:171776	17
abcb6	18
actr2	18
arglu1b	18
asna1	18
casp3a	18
creb1_dup1	18
irf2a	18
ltv1_dup1	18
mad1l1	18
mcoln1	18
pcca	18
pdcd4a	18
rab3d	18
ugdh	18
zgc:103633	18
zgc:123246	18
zgc:66331	18
zgc:77025	18
zgc:92182	18
zgc:92607	18
zgc:92638	18
apc	18
cltca	18
cxadr	18
ddx56	18
drg1	18
ehd1	18
hsdl2	18
htatsf1	18
nudcd3	18
ppp2r2d	18
prkrir	18
prrc1	18
ptpn11	18
ugcg	18
zgc:109973	18
zgc:136875	18
zgc:77449	18
zgc:92303	18
atp2b4	18
dynlrb1	18
hcfc1a	18

ptp4a1	18
rbb4l	18
rfc4	18
snrpe	18
zgc:110077	18
zgc:123327	18
zgc:162560	18
zgc:66285	18
zgc:76966	18
zgc:86798	18
adkb	18
cox7a2l	18
dlgap5	18
glud1b	18
map2k6_dup1	18
med24_dup1	18
msh2	18
mta3	18
pelo	18
ppm1a	18
tmem106a	18
ube2d1	18
zgc:158281	18
zgc:163009	18
zgc:171959	18
bmpr1a	18
c10orf119	18
chuk	18
eif3s10	18
gclc	18
gmfb	18
med6	18
npm3	18
pdlim1	18
pno1	18
rrp12	18
sufu	18
syf2	18
tmem57b	18
wdr32	18
zgc:109897	18
zgc:110547	18
zgc:153171	18
zgc:154071	18
zgc:158387	18
zgc:174910	18

brd8	18
cul4b	18
hspa4	18
mcm7	18
slc43a1a	18
slu7	18
stard10	18
thoc2	18
zgc:114040	18
zgc:136449	18
zgc:154116	18
zgc:172056	18
zgc:77049	18
akap1b	18
appbp2	18
atg16l1	18
cab39	18
capns1b	18
chchd2l	18
ebna1bp2l	18
ppm1d	18
ppp2r1b	18
rps6kb1	18
serpine2	18
tmem49	18
zgc:165618	18
akirin1	18
cdc42se1	18
derl1	18
ebag9	18
eif3h	18
gbp	18
gpd1l	18
prpf3	18
psma2	18
ptdss1	18
setdb1b	18
si:dkey-11p23.2	18
tex10	18
tpi1b	18
ttk	18
txndc4	18
u2af2a	18
usp5	18
utp11l	18
zgc:114097	18

zgc:153215	18
zgc:158838	18
zgc:63827	18
zgc:64042	18
zgc:77086	18
zgc:92790	18
arhgap11a	18
ccdc6a	18
dlst	18
hectd1	18
ktn1	18
nfkbiab	18
psma6a	18
si:busm1-142b24.1	18
sip1	18
smndc1	18
srp14	18
tpp1	18
yipf4	18
zgc:112399	18
zgc:113947	18
zgc:175220	18
zgc:55683	18
zgc:63779	18
zgc:92082	18
aars	18
akt2	18
bckdha	18
cotl1	18
dnaja2l	18
hsbp1	18
ilvbl	18
napa	18
sephs1	18
ss18	18
thoc1	18
ubl7	18
zgc:110418	18
zgc:56106	18
zgc:56258	18
zgc:86762	18
blzf1	18
ck2b	18
ctps	18
elmo1	18
erf	18

eya3	18
htatip	18
kifc1	18
kpnb1	18
lin28	18
med10	18
mfsd2b	18
ndufv1	18
nfyc	18
ptpn2l	18
rdbp	18
rxrba	18
seh1l	18
sept7a_dup1	18
sf3b4	18
sh3bgrl3	18
si:ch211-173p18.3	18
si:ch211-51m24.3	18
slc39a7	18
stk3	18
tpm3_dup2	18
twistnb	18
uqcrb	18
yars	18
ywhai	18
zgc:103632	18
zgc:85716	18
zgc:86598	18
ccdc94	18
copb2	18
cul3	18
fam49b	18
gpr177	18
gtf2b	18
gtpbp4	18
itgb1b	18
nedd8l	18
pccb	18
pdcd10b	18
prkci	18
prmt5	18
prpf38b	18
puf60a	18
ranbp9	18
rpl37	18
rpl5a	18

sec62	18
sec6l1	18
trnau1apl	18
upf1	18
yes1	18
zgc:56223	18
zgc:63523	18
zgc:92148	18
bxdc1	18
cdc40	18
gmds	18
gopc	18
gtf2h5	18
hif1ab	18
hsf2	18
paics	18
psma8	18
psmc6	18
si:ch211-181h6.2	18
snx3	18
vta1	18
zc3h14	18
zgc:112104	18
zgc:123193	18
zgc:92217	18
zgc:92765	18
arid3b	18
atp2a2b	18
cast	18
dnl2l	18
dusp4	18
hnrnph1l	18
itm1	18
LOC325449	18
LOC561184	18
lrrc8a_dup2	18
nnt	18
nop14_dup2	18
pfdn1	18
pgrmc1	18
rbm22	18
sdf2	18
smarcb1b	18
taf9	18
ube2g1	18
zgc:101768	18

zgc:110605	18
zgc:110810	18
zgc:158750	18
zgc:165523	18
zgc:56334	18
zgc:66100	18
zgc:77151	18
zgc:92775	18
zgc:92910	18
aspm	18
chaf1a	18
dstyk	18
fxr1	18
gatad2a	18
hdgfrp2	18
hiat1b	18
LOC565969	18
lsm7	18
map2k2	18
pias4	18
rtcd1	18
sec13	18
si:ch211-103f16.2	18
si:ch211-197i12.3	18
si:ch211-81i17.1	18
si:dkeyp-87e7.4	18
smc3	18
tmem161a	18
zgc:162288	18
zgc:171566	18
zgc:63733	18
zgc:92895	18
abce1	18
copz1	18
lmbr1l	18
nsfl1c	18
rprd1b	18
sdcbp	18
sps2	18
taf13	18
tpd52l2b	18
zgc:103534	18
zgc:63600	18
acvr2b	18
dap1a	18
grnb	18

hccs	18
mynn	18
ppp4r1	18
prpf4b	18
rrs1	18
tbca	18
txndc5	18
ube2v2	18
vapa	18
cops2	18
nap1l4a	18
psma4	18
saps3	18
tsg101	18
zgc:110339	18
zgc:114121	18
zgc:114188	18
zgc:158610	18
zgc:162316	18
zgc:77675	18
aldh3d1	18
arf2	18
arhgdia	18
bcat2	18
carm1	18
cdc73	18
dnajc7	18
eftud2	18
eif3d	18
eif3g	18
gna13b	18
mapk3	18
myst1	18
pold1	18
rhot1a	18
sepw2a	18
smarca4	18
tlk2	18
zc3h7	18
zgc:110766	18
zgc:136380	18
zgc:162578	18
zgc:55645	18
zgc:63569_dup1	18
zgc:63569_dup2	18
zgc:92241	18

aebp2	18
ahcyl2	18
apex1	18
cct2_dup2	18
cpsf6	18
fam3c	18
hspa14	18
ing3	18
lemd3	18
mdm2	18
nup107	18
nup50	18
pphln1	18
psmc2	18
si:ch211-214j24.10	18
si:dkey-222f8.3	18
si:dkey-22l11.2	18
slc35e3	18
zc3hc1	18
zgc:55466	18
zgc:92883	18
arl6ip4	18
bcl7a	18
cox7c	18
dhfr	18
eif4e1b	18
elac1	18
gats	18
grsf1	18
imp4	18
oclnb	18
polr2j	18
prps1a	18
rasgef1ba	18
rdx	18
rfc5_dup2	18
sart3	18
sepw1	18
si:ch211-191d15.2	18
si:ch211-278b8.3	18
si:dkey-273o13.2	18
tp53	18
tsc1a	18
ube2l3l	18
usf2	18
usp39	18

vkorc1l1	18
zcchc8	18
zfr	18
zgc:101016	18
zgc:103697	18
zgc:152816	18
arpc4	18
capza1	18
cyfip1	18
ddi2	18
ddx27_dup1	18
ddx27_dup2	18
edem1	18
eif4bb	18
gnai2	18
itm2ba	18
mier1a	18
pomgnt1	18
prpf38a	18
psmd12	18
rp2	18
stk35l	18
sucla2	18
tarbp2	18
tardbp	18
tlk1b	18
utp6	18
zgc:109901	18
zgc:113054	18
zgc:113842	18
zgc:65802_dup1	18
zgc:66479	18
zgc:76877	18
zgc:85694	18
ap1s1	18
aph1b	18
arl8	18
brd7	18
caprin1a	18
cnpy4	18
dvl2	18
fxr2	18
ipo7	18
kars	18
lin7c	18
mta2	18

ostc	18
pofut1	18
psmc3	18
psmd7	18
serf2	18
slc7a6	18
vrk3	18
zgc:112305	18
zgc:153243	18
zgc:162265	18
zgc:171549	18
zgc:55468	18
zgc:55671	18
zgc:92818	18
adipor1b	18
aldh9a1a	18
arih2	18
cenpa	18
chmp4b	18
crsp8	18
dohh	18
dt1p1a10l	18
fzr1	18
gclm	18
LOC555288	18
LOC559707	18
nt5dc2	18
oxct1a	18
polb	18
si:dkey-72l14.8	18
sin3b	18
srpk1	18
vdac3	18
zgc:114058	18
zgc:152990	18
zgc:55572	18
zgc:56036	18
zgc:73373	18
znf367	18
actr3	18
arpc2_dup2	18
asnsd1	18
chaf1b	18
mobkl3	18
phgdh	18
pwp2h	18

si:ch211-140m22.6	18
si:dkey-234n3.1	18
u2af1	18
zgc:123170	18
zgc:153452	18
zgc:171542	18
zgc:55970	18
akap8l	19
anxa5b	19
atp1a1	19
cct4	19
gtf2e2	19
plrg1	19
smarca5	19
smc2	19
zgc:158239	19
cct8	19
dcps	19
hic2	19
hnrpdl	19
lmnb1	19
mdh1a	19
rps25	19
skiv2l2	19
ube2l3	19
zgc:110695	19
zgc:86753	19
alas1	19
mapkapk2a	19
psmd6	19
rbm39a	19
sfrs11	19
snai1a	19
trpc4apa	19
wdr18	19
zgc:154063	19
zgc:55741	19
aco2	19
foxh1	19
myst2	19
pald	19
rpl38	19
sfrs2	19
slc16a8	19
wac	19
zgc:113026	19

zgc:76940	19
cdc25	19
cnih	19
ddx21	19
idh2	19
lgmn	19
sf3b5	19
si:dkey-33c12.4	19
tm9sf3	19
tmem50a	19
zgc:110154	19
zgc:113383	19
cstf2	19
ctbp1	19
lman2	19
polr2gl	19
rpl26	19
smad5	19
tspan7	19
crk	19
dnl2	19
prpf8	19
sae1	19
smc4	19
spcs2	19
zgc:123178	19
zgc:77282	19
cdc42l	19
ck2a1	19
psmc4	19
stt3b	19
tomm40	19
wdsof1	19
zgc:136346	19
zgc:92656	19
ahsa1l	19
calm1a	19
cdc5l	19
odc1	19
pgrmc2	19
spint1a	19
syncripl	19
wdr43	19
yth2	19
zgc:114044	19
bcl2l10	19

cox4i1	19
cstf3	19
eif3m	19
morf4l1	19
rab11a	19
rpl35a	19
sec11a	19
sf3b3	19
ssr3	19
st14a	19
zgc:92014	19
azin1	19
brd2a	19
igf2bp3	19
ndrg1	19
nsun2	19
nudc	19
SFRS4	19
zgc:100869	19
zgc:109888	19
zgc:171646	19
zgc:55573	19
acvr1l	19
arf1	19
ccnl1	19
cnn3b	19
glula	19
homez	19
ifi30	19
ing5b	19
pfn2l	19
ppp1r7	19
psmb5	19
rnf2	19
selt1a	19
15-Sep	19
sf3a2	19
tcea1	19
tmem48	19
zgc:195633	19
akirin2	19
c6orf115	19
cad	19
eif5	19
esco2	19
lbr	19

LOC557217	19
pdip5	19
sf3b14	19
si:dkeyp-114g9.1	19
smek1	19
tmed10	19
tmed5	19
wtap	19
zgc:110155	19
zgc:92757	19
brd3a	19
calm3b	19
cdk9	19
lrrc8a_dup1	19
psmb7	19
sept8a	19
skp2	19
wdr5	19
zgc:136471	19
zgc:56064	19
zgc:56513	19
zgc:64137	19
zgc:65996	19
zmat2	19
fam32al	19
mcm2	19
smarcd1	19
zgc:112095	19
zgc:55690	19
acvr1b	19
hm13	19
pip5k2	19
ppp4r2a	19
racgap1	19
rhoac	19
tardbpl	19
tcp1	19
tm9sf4	19
zgc:55695	19
zgc:56304	19
zgc:86751	19
cct5	19
eif2s3	19
ezh2	19
mcm4	19
rpl21	19

tram1	19
fbxl14a	19
gnaia	19
lsm14a	19
slc25a3	19
uba2	19
zgc:154017	19
zgc:173652	19
aldoaa	19
arpc1a	19
casc3	19
cbx1a	19
cbx2	19
hn1l	19
kdelr2	19
llgl1	19
mcm5	19
metrnl	19
mettl2a	19
phb	19
rnps1	19
rps11	19
rras	19
spns1	19
tceb2	19
tob1a	19
wdr68	19
ypel3	19
zgc:55558	19
zgc:64163	19
adipor2	19
atp2b1a	19
cct2_dup1	19
cd9l	19
gdi2	19
rps16	19
si:dkey-159a18.7	19
tmpo	19
usp44	19
zgc:110552	19
bckdk	19
capns1a	19
coro1c	19
edf1	19
ppp2r1a	19
ppp6c	19

prpf4	19
rpl36a	19
ruvbl2	19
slc37a2	19
srrt	19
sub1	19
ube2g2	19
zgc:110137	19
zgc:158409	19
zgc:158618	19
zgc:73070	19
actl6a	19
amotl2	19
atf1	19
blcap	19
cyc1	19
eif2s2	19
npepl1	19
pa2g4a	19
psmb3	19
rps26l	19
tomm70a	19
tuba8l4	19
zgc:152914	19
zgc:65802_dup2	19
aktip	19
copb1	19
rpl13	19
rpl34	19
rps13	19
slc38a7	19
supt16h	19
zgc:112356	19
zgc:136773	19
cry2a	19
dnttip2	19
fnbp1l	19
mapk14a	19
mobkl2a	19
nras	19
rap1a	19
sap30bp	19
sf3a1	19
si:dkey-72l14.9	19
suv39h1b	19
uck2a	19

unc119.1	19
zgc:63791	19
zgc:92164	19
arpc2_dup1	19
dbr1	19
ddx18	19
hspd1	19
prpf40a	19
pttg1ip	19
si:dkey-67c22.2	19
