# Supplementary material for: The zebrafish transcriptome during early development
Source: BMC Dev Biol. 2011 May 24;11:30. doi: 10.1186/1471-213X-11-30 (PMC3118190; doi:10.1186/1471-213X-11-30)
Supplement: Additional file 5 — Significant enrichment of GO molecular functions. A) GO molecular functions significantly enriched for the four increasing clusters. B) GO molecular functions significantly enriched within the three clusters displaying a decrease in transcript abundance during development (p < 0.01). [file 1471-213X-11-30-S5.PDF]

A.

GO Molecular Function				
Gene Ontology term	Cluster frequency	Genome frequency	P-value	Genes annotated to the GO term
nucleic acid binding	34 of 93 genes, 36.6%	2094 of 22409 genes, 9.3%	$5.30E^{-11}$	zgc:110216, cebpb, vox, zgc:101846, zgc:171937, zgc:173552, zgc:77366, akap12, h3f3a, hnrnpa0, zgc:112234, sox3, hnrpl, zgc:153405, sox19a, rbmx, zic2b, h2afvl, h2afx, cirbp, ef1a, zgc:158350, tbx16, ved, zgc:153409, ddx5, zgc:163047, sfpq, hnrnpa0l, khdrbs1a, rps5, zgc:154164, ntla, ybx1
structural molecule activity	13 of 93 genes, 14.0%	319 of 22409 genes, 1.4%	$4.33E^{-08}$	krt18, rps15a, krt4, krt8, cki, zgc:92061, krt5, zgc:109868, zgc:123194, rps5, cldne, rps21, rpl27
DNA binding	21 of 93 genes, 22.6%	1112 of 22409 genes, 5.0%	$2.46E^{-07}$	zgc:110216, tbx16, cebpb, vox, zgc:101846, ved, zgc:171937, zgc:173552, akap12, h3f3a, zgc:153409, zgc:163047, sox3, zgc:112234, zgc:153405, sox19a, zgc:154164, h2afx, h2afvl, ntla, ybx1
binding	49 of 93 genes, 52.7%	7042 of 22409 genes, 31.4%	0.00097	zgc:195154, zgc:110216, zgc:55813, cebpb, hspa8, vox, zgc:101846, zgc:171937, zgc:173552, zgc:77366, zgc:123194, akap12, h3f3a, zgc:85717, hnrnpa0, anp32a, zgc:112234, sox3, hnrpl, zgc:153405, sox19a, rbmx, zic2b, bactin2, ak3, h2afx, h2afvl, cirbp, ef1a, zgc:158350, tbx16, anp32e, ved, zgc:153409, marcksl1, ddx5, zgc:163047, slc25a22, sfpq, cxcr4b, hnrnpa0l, khdrbs1a, rps5, zgc:154164, zgc:110251, apoeb, cxcr4a, ntla, ybx1

B.

GO Molecular Function				
Gene Ontology term	Cluster frequency	Genome frequency	P-value	Genes annotated to the GO term
polo kinase kinase activity	2 of 84 genes, 2.4%	3 of 22409 genes, 0.0%	0.00257	plk1, plk3
