# Supplementary material for: The zebrafish transcriptome during early development
Source: BMC Dev Biol. 2011 May 24;11:30. doi: 10.1186/1471-213X-11-30 (PMC3118190; doi:10.1186/1471-213X-11-30)
Supplement: Additional file 6 — The GO term enrichment in developmental-stage specific transcripts. The significantly enriched GO terms for transcripts found only in one out of the four studied developmental stages were investigated. A) Significantly enriched cellular component and biological processes for the 1-cell stage. Out of the 170 transcripts uniquely found in this stage 47 transcripts were unknown. B) Enrichment of GO molecular function in the 150 transcripts uniquely found in the 16-cell stage. In this set 55 transcripts were unknown. C) Among the 100 transcripts found only in the 512-cell stage 31 were unknown and the remaining 69 were enriched for 2 GO molecular functions. D) Significantly enriched GO cellular components and molecular functions among the transcripts found exclusively in the 50% epiboly stage. Out of the 440 transcripts in this group 125 were unknown. (p < 0.01). [file 1471-213X-11-30-S6.PDF]

A.

GO Cellular Component				
GO term	Cluster frequency	Genome frequency	P-value	Genes annotated to the term
extracellular region	13 of 168 genes, 7.7%	439 of 22409 genes, 2.0%	0.00116	edn1, hbl4, npy, igfbp5b, wnt2, thbs4b, vegfaa, ism2, gdf6b, si:dkeyp-59a8.1, wnt9b, igfbp1b, gnrh3

GO Biological Process				
GO term	Cluster frequency	Genome frequency	P-value	Genes annotated to the term
multicellular organismal process	34 of 168 genes, 20.2%	1741 of 22409 genes, 7.8%	5.14E-05	gcm2, tal1, otpa, atp1a1a.2, rbp4, crfb12, sox14, dub, isl2b, msxa, edn1, slit1b, nkx2.1a, musk, wnt2, ikzf1, dmbx1a, vmhc, nadl1.1, mfng, hoxc12b, crfb1, ascl1b, fezf1, vegfaa, sox1b, myod1, wnt9b, hoxa9a, olfm1a, crfb7, cnr1, acvrl1, gnrh3
Develop-mental process	33 of 168 genes, 19.6%	1681 of 22409 genes, 7.5%	7.16E-05	gcm2, tal1, otpa, atp1a1a.2, rbp4, sox14, dub, isl2b, msxa, edn1, slit1b, nkx2.1a, musk, wnt2, hspa12b, ikzf1, dmbx1a, vmhc, nadl1.1, mfng, hoxc12b, angptl1, ascl1b, vegfaa, fezf1, sox1b, myod1, wnt9b, hoxa9a, olfm1a, cnr1, acvrl1, gnrh3
multicellular organismal development	31 of 168 genes, 18.5%	1529 of 22409 genes, 6.8%	8.78E-05	gcm2, tal1, ascl1b, fezf1, vegfaa, otpa, atp1a1a.2, rbp4, sox1b, myod1, wnt9b, hoxa9a, sox14, dub, isl2b, olfm1a, msxa, edn1, slit1b, nkx2.1a, musk, wnt2, ikzf1, cnr1, acvrl1, dmbx1a, vmhc, nadl1.1, mfng, gnrh3, hoxc12b
thyroid gland development	3 of 168 genes, 1.8%	8 of 22409 genes, 0.0%	0.00572	vegfaa, tal1, nkx2.1a
anatomical structure development	25 of 168 genes, 14.9%	1354 of 22409 genes, 6.0%	0.00687	gcm2, tal1, angptl1, ascl1b, fezf1, vegfaa, otpa, atp1a1a.2, rbp4, myod1, sox14, dub, olfm1a, edn1, slit1b, nkx2.1a, musk, hspa12b, ikzf1, cnr1, acvrl1, dmbx1a, vmhc, nadl1.1, gnrh3

B.

GO Molecular Function				
GO term	Cluster frequency	Genome frequency	P-value	Genes annotated to the term
nucleic acid binding transcription factor activity	14 of 149 genes, 9.4%	653 of 22409 genes, 2.9%	0.0081	esr2b, meis2.2, zgc:162612, pax6a, gata4, prrx1a, tbx22, dmrta2, tlx3b, otx5, gata2b, barhl1.2, zgc:158291, hoxc13b
sequence-specific DNA binding transcription factor activity	14 of 149 genes, 9.4%	653 of 22409 genes, 2.9%	0.0081	esr2b, meis2.2, zgc:162612, pax6a, gata4, prrx1a, tbx22, dmrta2, tlx3b, otx5, gata2b, barhl1.2, zgc:158291, hoxc13b
transcription regulator activity	12 of 149 genes, 8.1%	497 of 22409 genes, 2.2%	0.00846	pax6a, gata4, prrx1a, myf6, tlx3b, twist3, atoh1a, otx5, barhl1.2, zgc:158291, hoxc13b, si:ch211-130m23.3

C.

GO Molecular Function				
GO term	Cluster frequency	Genome frequency	P-value	Genes annotated to the term
nucleic acid binding transcription factor activity	11 of 100 genes, 11.0%	653 of 22409 genes, 2.9%	0.00798	nr5a5, barhl2, isl1, irf9, dlx1a, nkx2.7, fev, hmx4, nr2f5, irf2, dlx2b
sequence-specific DNA binding transcription factor activity	11 of 100 genes, 11.0%	653 of 22409 genes, 2.9%	0.00798	nr5a5, barhl2, isl1, irf9, dlx1a, nkx2.7, fev, hmx4, nr2f5, irf2, dlx2b

D.

GO Cellular Component				
GO term	Cluster frequency	Genome frequency	P-value	Genes annotated to the term
cell junction	12 of 436 genes, 2.8%	161 of 22409 genes, 0.7%	0.0058	cx28.8, zgc:172282, snap25b, slc17a8, cldnf, gabra1, ada, si:ch211-251b21.1, zgc:112437, gria3b, xirp1, zgc:110625

GO Molecular Function				
GO term	Cluster frequency	Genome frequency	P-value	Genes annotated to the term
sequence-specific DNA binding	27 of 436 genes, 6.2%	528 of 22409 genes, 2.4%	0.00077	eng1b, bon, cdx1a, emx1, lhx2a, dlx3b, six7, onecutl, pnx, cebpd, foxc1a, hlxb9la, mafg1, hlx1, pax7a, dbx1a, pitx2, nr2f6b, mxtx1, lhx1b, foxb1.1, nr2e1, cebpa, nkx2.9, eng2b, msxe, msxc
receptor binding	17 of 436 genes, 3.9%	263 of 22409 genes, 1.2%	0.00237	igf3, gdf11, adcyap1a, fgf12, epo, fgf4, tnfsf10l, pth2, fgf3, cxcl12b, amh, fgl2, mdka, crh, fgf19, tnfb, adm2
nucleic acid binding transcription factor activity	29 of 436 genes, 6.7%	653 of 22409 genes, 2.9%	0.00494	eng1b, bon, cdx1a, emx1, lhx2a, dlx3b, six7, onecutl, tbx6, pnx, cebpd, foxc1a, hlxb9la, mafg1, hlx1, pax7a, dbx1a, pitx2, nr2f6b, mxtx1, lhx1b, foxb1.1, smad1, nr2e1, cebpa, nkx2.9, eng2b, msxe, msxc
sequence-specific DNA binding transcription factor activity	29 of 436 genes, 6.7%	653 of 22409 genes, 2.9%	0.00494	eng1b, bon, cdx1a, emx1, lhx2a, dlx3b, six7, onecutl, tbx6, pnx, cebpd, foxc1a, hlxb9la, mafg1, hlx1, pax7a, dbx1a, pitx2, nr2f6b, mxtx1, lhx1b, foxb1.1, smad1, nr2e1, cebpa, nkx2.9, eng2b, msxe, msxc
