# Supplementary material for: The zebrafish transcriptome during early development
Source: BMC Dev Biol. 2011 May 24;11:30. doi: 10.1186/1471-213X-11-30 (PMC3118190; doi:10.1186/1471-213X-11-30)
Supplement: Additional file 7 — Gene transcript assayed using TaqMan low density array (TLDA). The TLDA gene expression assay numbers (assay ID), the RefSeq accession numbers (RefSeq) and the official gene symbol for the assayed transcripts are given in the table. In addition, the amplicon length is given. Transcripts further assayed using semi-quantitative RT-PCR are marked with a *. [file 1471-213X-11-30-S7.PDF]

Assay ID	Gene Symbol	RefSeq	Amplicon Length
Dr03124909_g1	acvr1b	NM_130990.1	90
Dr03100459_m1	aida	NM_200790.1	81
Dr03136793_m1	bida	NM_001079826.1	82
Dr03118800_m1	bmp4	NM_131342.2	106
Dr03201341_s1	cldna	NM_131762.2	85
Dr03201356_s1	cldnb	NM_131763.2	92
Dr03125415_s1	cldnd	NM_180964.2	75
Dr03433163_s1	cldne	NM_131765.1	79
Dr03119161_m1	crabp2a	NM_182859.1	82
Dr03075744_g1	elmo1	NM_213091.1	88
Dr03093217_m1	foxa2*	NM_130949.1	77
Dr03118760_s1	foxd3	NM_131290.1	141
Dr03099333_m1	foxh1	NM_131502.1	90
Dr03080601_m1	id2b	NM_199541.1	112
Dr03138193_g1	kita	NM_131053.1	93
Dr03080183_m1	klf4*	NM_131723.1	84
Dr03106149_m1	mapk1	NM_182888.2	98
Dr03438522_gH	mapk3	NM_201507.1	85
Dr03129596_m1	nrg1	NM_001044911.1	125
Dr03092829_m1	nrg2a	NM_001099254.1	81
Dr03150181_m1	oep	NM_131092.1	117
Dr03080707_g1	pdia4	NM_199779.1	105
Dr03423121_m1	pink1	NM_001008628.1	103
Dr03150185_g1	pou5f1*	NM_131112.1	90
Dr03435368_m1	ptena	NM_200708.1	102
Dr03108160_m1	ptenb	NM_001001822.2	105
Dr03145359_m1	slc25a1	NM_200607.1	92
Dr03140047_g1	slc25a22	NM_213408.1	86
Dr03107933_g1	slc25a25	NM_213257.1	79
Dr03071128_m1	slc25a46	NM_001002558.1	83
Dr03429158_m1	slc26a1	NM_001080667.1	72
Dr03099933_m1	slc26a11	NM_199767.1	126
Dr03103605_m1	slc2a1	NM_001039808.1	106
Dr03148071_m1	slc2a15b	NM_001020494.1	92
Dr03136084_m1	slc2a2	NM_001042721.1	97
Dr03129904_g1	slc35f2	NM_001076556.1	80
Dr03120560_m1	slc39a1	NM_212583.2	79
Dr03425622_g1	slc39a6	NM_001001591.1	71
Dr03195662_s1	slc39a9*	NM_001013540.1	62
Dr03101823_mH	slc48a1a	NM_001002424.1	101
Dr03434511_m1	slc48a1b	NM_200006.1	130
Dr03148494_m1	slc6a4a	NM_001039972.1	115
Dr03121858_g1	slc6a5	NM_001009557.1	104
Dr03135920_m1	slc6a6	NM_001037661.1	101
Dr03194710_s1	slc7a3	NM_001007329.2	73
Dr03140998_m1	slc7a6os	NM_001007344.1	95
Dr03147497_m1	slc9a8	NM_001008586.1	133
Dr03203853_s1	sox2	NM_213118.1	165
Dr03425637_g1	sox3	NM_001001811.2	109
Dr03147028_g1	suv39h1a	NM_001003592.1	91
Dr03429785_m1	suv39h1b	NM_001126482.1	86
Dr03074863_m1	tbp	NM_200096.1	92
Dr03105238_m1	tdrd7	NM_001099343.1	93
Dr03112089_m1	tp53	NM_131327.1	93
Dr03120601_m1	xpo4	NM_212674.1	105
Dr03106195_g1	xpo6	NM_194374.1	112
Dr03102066_m1	zgc:100930	NM_001003630.1	91
Dr03071371_m1	zgc:101015	NM_001004002.1	101
Dr03083473_m1	zgc:112079	NM_001012384.1	60
Dr03096426_g1	zgc:113210	NM_001013514.1	91
Dr03103864_m1	zgc:136551	NM_001044848.1	92
Dr03152184_g1	zgc:55877	NM_213070.1	84
Dr03086973_g1	zic2a	NM_131558.1	68
Dr03425656_g1	zic2b	NM_001001820.1	77
