# Supplementary material for: The zebrafish transcriptome during early development
Source: BMC Dev Biol. 2011 May 24;11:30. doi: 10.1186/1471-213X-11-30 (PMC3118190; doi:10.1186/1471-213X-11-30)
Supplement: Additional file 9 — RPKM values for a selected subset of developmentally expressed genes. The table gives the RPKM values for a subset of developmentally expressed genes that are discussed in the present study. [file 1471-213X-11-30-S9.PDF]

Developmental stage gene expression level (RPKM)					
Transcript ID	RefSeq ID	1-cell stage	16-cell stage	512-cell stage	50% epiboly
cldnd	NM_180964	2290	2711	2210	188
cldnf	NM_131766	0	0	0	35
cldng	NM_180965	2318	2210	1453	157
eif1b	NM_199588	162	151	178	384
elovl2	NM_001040362	1	0	0	0
foxh1	NM_131502	78	84	92	237
ing5	NM_001100049	2	1	0	0
krt8	NM_200080	18	20	37	1505
krt18	NM_178437	0	0	10	1993
plk1	NM_001003890	588	612	574	294
plk2	NM_001099245	4	4	4	0
plk3	NM_201308	434	459	440	210
plk4	NM_001118892	60	58	52	30
sfxn2	NM_212730	0	0	0	0
slc39a7	NM_130931	70	91	97	68
tia1l	NM_200182	335	315	353	490
tra2a	NM_200416	195	185	204	497
