# Supplementary material for: The zebrafish transcriptome during early development
Source: BMC Dev Biol. 2011 May 24;11:30. doi: 10.1186/1471-213X-11-30 (PMC3118190; doi:10.1186/1471-213X-11-30)
Supplement: Additional file 13 — Slc gene superfamily members detected using RNA-Seq. Gene transcripts from 154 different members of the Slc gene superfamily were detected in the four studied developmental stages. [file 1471-213X-11-30-S13.PDF]

slc11a2	slc25a10	slc26a5	slc37a2	slc5a9
slc12a1	slc25a12	slc27a1	slc37a4	slc6a11
slc12a10.1	slc25a14	slc27a2	slc38a3	slc6a13
slc12a10.2	slc25a16	slc27a6	slc38a4	slc6a13l
slc12a10.3	slc25a20	slc2a1	slc38a6	slc6a19
slc12a2	slc25a21	slc2a12	slc38a7	slc6a3
slc12a3	slc25a22	slc2a15a	slc39a1	slc6a4a
slc12a9	slc25a24l	slc2a15b	slc39a10	slc6a6
slc13a1	slc25a25	slc2a8l	slc39a13	slc6a9
slc13a2	slc25a25b	slc30a1	slc39a3	slc7a2
slc15a1	slc25a26	slc30a10	slc39a6	slc7a3
slc15a2	slc25a27	slc30a4	slc39a7	slc7a4
slc16a1	slc25a28	slc30a5	slc39a9	slc7a6
slc16a12b	slc25a29	slc30a7	slc3a2	slc7a6os
slc16a3	slc25a3	slc30a7	slc40a1	slc8a1a
slc16a8	slc25a32a	slc31a1	slc43a1a	slc8a1b
slc16a9a	slc25a32b	slc33a1	slc43a2	slc8a2a
slc16a9b	slc25a33	slc34a2a	slc44a4	slc8a2b
slc16a9b	slc25a36a	slc34a2aas	slc45a2	slc8a3
slc17a6l	slc25a37	slc35a5	slc46a1	slc8a4a
slc17a7	slc25a3l	slc35b1	slc48a1a	slc8a4b
slc17a8	slc25a4	slc35b2	slc48a1b	slc9a2
slc17a9b	slc25a43	slc35b3_dup1	slc4a1	slc9a3.1
slc1a2	slc25a44a	slc35b3_dup2	slc4a11	slc9a3.2
slc1a3	slc25a44b	slc35b4	slc4a2a	slc9a3r2
slc1a4	slc25a46	slc35c2	slc4a2b	slc9a5
slc1a7	slc25a5_dup1	slc35d1a	slc4a4	slc9a6a
slc20a1a	slc25a5_dup2	slc35d1b	slc5a1	slc9a6b
slc20a1b	slc25a6	slc35e1	slc5a11	slc9a7
slc22a18	slc26a1	slc35e3	slc5a5	slc9a8
slc25a1	slc26a11	slc35f2	slc5a8l	
