# Supplementary material for: The zebrafish transcriptome during early development
Source: BMC Dev Biol. 2011 May 24;11:30. doi: 10.1186/1471-213X-11-30 (PMC3118190; doi:10.1186/1471-213X-11-30)
Supplement: Additional file 15 — Primers used in RT-PCR gene expression analysis. Table of RT-PCR primers used for the study of gene expression of selected genes expressed during early zebrafish development. * denotes endogenous control. [file 1471-213X-11-30-S15.PDF]

RefSeq ID	Primer name	Primer sequence (5’-3’)
NM_199588	5’-eif1b	TCGTATGTCCGCTATCCAGA
	3’-eif1b	CCTGAACCGTGGTCAGAGTC
NM_131284	5’-forkheadboxA1	CTCAGCACAGCTCCATGAAC
	3’-forkheadboxA1	GCGGGTAACTCCTCCTGAA
NM_130949	5’-forkheadboxA2	ATGCTCGGTGCTGTCAAA
	3’-forkheadboxA2	CAAGTCCAGTGTTCATGTTGC
NM_131299	5’-forkheadboxA3	TCCTTTCTACAGTGAGGCAAA
	3’-forkheadboxA3	TAGCCCATATTCATGCTGGA
NM_131723	5’-krüppel-like factor 4	TTAAGCCCAGAAGACAGCAAG
	3’-krüppel-like factor 4	GCATGTGCGCTTTCAAAT
NM_001003890	5’-polo-like kin_1	GTACTTGCACAACAACAGAGTCA
	3’-polo-like kin_1	CTCAATCTTAGTGGCTAAACCAAAG
NM_131112	5’-pou5f1	CAGTGGAGGATGCAGTGATT
	3’-pou5f1	TTGCCATACAAGTTTCCAAGC
NM_212730	5’-sfxn2	TGCAGTTGGCCTTAACCTCTAC
	3’-sfxn2	CACAGCAATGCCATGCAA
NM_130931	5’-slc39a7	TGTTACCACCATCACCATCC
	3’-slc39a7	TTCCAGCTAATGCACCAACA
NM_001013540	5’-slc39a9	ATGAGGAAATGCTGGAAGGT
	3’-slc39a9	AATCCCAATGTAAGCGTGGA
NM_200182	5’-tia1l	TCTGAACCAGTCGAGTCCTAGT
	3’-tia1l	TCTCCATTATCTGGCCGAAC
NM_200416	5’-transformer-2 a	CCGGTGTCAATGTTGTCTATG
	3’-transformer-2 a	TGCGTCTTCCATCTAGTTCC
NM_001045269	5’-zgc:136359	TCAGAGACTATTGGGAGTGGA
	3’-zgc:136359	TTGACAGGCGAGTTGTTAGAG
NM_212784*	5’-rpl13a	TCTGGAGGACTGTAAGAGGTATGC
	3’-rpl13a	AGACGCACAATCTTGAGAGCAG
NM_181601*	5’-bactin2	CGAGCTGTCTTCCCATCCA
	3’-bactin2	TCACCAACGTAGCTGTCTTTCCG
